# Inferring number of populations and changes in connectivity under the n-island model

**DOI:** 10.1038/s41437-021-00426-9

**Published:** 2021-04-12

**Authors:** Armando Arredondo, Beatriz Mourato, Khoa Nguyen, Simon Boitard, Willy Rodríguez, Olivier Mazet, Lounès Chikhi

**Affiliations:** 1 Université de Toulouse, Institut National des Sciences Appliquées, Institut de Mathématiques de Toulouse, Toulouse, France; 2 Institut de Mathématiques de Toulouse; UMR5219. Université de Toulouse, Toulouse, France; 3 Instituto Gulbenkian de Ciência, Oeiras, Portugal; 4 CBGP, Université de Montpellier, CIRAD, INRAE, Institut Agro, IRD, Montpellier, France; 5 ENAC - Ecole Nationale de l’Aviation Civile, Université de Toulouse, Toulouse, France; 6 Laboratoire Évolution & Diversité Biologique (EDB UMR 5174), CNRS, IRD, UPS, Université de Toulouse Midi-Pyrénées, Toulouse, France

**Keywords:** Population genetics, Biological models, Population genetics

## Abstract

Inferring the demographic history of species is one of the greatest challenges in populations genetics. This history is often represented as a history of size changes, ignoring population structure. Alternatively, when structure is assumed, it is defined a priori as a population tree and not inferred. Here we propose a framework based on the IICR (Inverse Instantaneous Coalescence Rate). The IICR can be estimated for a single diploid individual using the PSMC method of Li and Durbin (2011). For an isolated panmictic population, the IICR matches the population size history, and this is how the PSMC outputs are generally interpreted. However, it is increasingly acknowledged that the IICR is a function of the demographic model and sampling scheme with limited connection to population size changes. Our method fits observed IICR curves of diploid individuals with IICR curves obtained under piecewise stationary symmetrical island models. In our models we assume a fixed number of time periods during which gene flow is constant, but gene flow is allowed to change between time periods. We infer the number of islands, their sizes, the periods at which connectivity changes and the corresponding rates of connectivity. Validation with simulated data showed that the method can accurately recover most of the scenario parameters. Our application to a set of five human PSMCs yielded demographic histories that are in agreement with previous studies using similar methods and with recent research suggesting ancient human structure. They are in contrast with the view of human evolution consisting of one ancestral population branching into three large continental and panmictic populations with varying degrees of connectivity and no population structure within each continent.

## Introduction

Reconstructing the demographic history of populations from the analysis of genomic data is one of the greatest challenges of population geneticists (Beaumont, 2004, Goldstein and Chikhi, 2002, Hey and Machado, 2003, Johri et al., 2020, Li and Durbin, 2011). It is an important and challenging statistical problem, but it is also central to our understanding of the evolutionary history of species. Indeed, the demographic history that we assume or infer for a particular population or species implicitly or explicitly provides the null model against which selected loci could in theory be identified (Beaumont and Nichols, 1996, Cavalli-Sforza, 1966, Goldstein and Chikhi, 2002, Johri et al., 2020). In a period of global environmental change, the reconstructed demographic history should allow evolutionary biologists to correlate changes in population size or connectivity with past environmental changes or species association and interactions (Goossens et al., 2006, Hecht et al., 2018, 2020, Mona et al., 2014, Quéméré et al., 2012, Salmona et al., 2017).

In other words, by addressing these challenges we expect to increase our understanding of the environmental (including species interactions) and anthropogenic factors that have influenced genomic diversity in various species. Also, understanding how past climatic events or human activities have influenced genomic diversity today may become particularly illuminating for conservation biologists regarding the likely long-term consequences of ongoing climate change and human actions (Poelstra et al., 2021).

However, to understand how the past influenced the present patterns of genomic diversity one major question is whether our conclusions or inferences may fundamentally change depending on the family of models assumed and the questions asked (Beaumont, 2004, Chikhi et al., 2018, 2010, Mazet et al., 2016, Pouyet et al., 2018, Rodríguez et al., 2018, Wakeley, 1999). Currently, the solutions to this complex inferential problem have been to assume that differentiation between geographic locations can be neglected and local panmixia assumed, and then infer population size changes (Li and Durbin, 2011, Liu and Fu, 2015). Alternatively, other studies have assumed simplified tree models with a priori fixed numbers of populations (i.e., the population trees are not inferred). Additionally, in the case of human evolutionary history, the branches of the assumed tree may represent predefined continental populations. Such models thus assume panmixia over large geographic regions and long periods (Gutenkunst et al., 2009, Noskova et al., 2019). Panmictic and tree models are useful approximations, and in the last decades they have proven their utility in building stories of human expansions and population splits (Gutenkunst et al., 2009, Li and Durbin, 2011). However, the meaning of such stories is questionable (Mazet et al., 2016, Scerri et al., 2019, Wakeley, 1999). Also, most tree models assume the existence of clear splitting events similar to those separating species, and some tree models assume, as in most species trees, that there is no gene flow between branches (even when they represent populations or continents). The latter assumption may then require the inference of admixture events (e.g., Kuhlwilm et al., 2016).

Methods can also be classified by the type of data used. Currently, most genomic methods use the allele frequency spectrum (AFS) (Excoffier et al., 2013, Gutenkunst et al., 2009, Liu and Fu, 2015) or the AFS combined with other summary statistics (Boitard et al., 2016). The AFS can be computed from RAD-Seq data for many non-model species (Poelstra et al., 2021) or from full genome sequences for a still limited number of species (Lapierre et al., 2017). We stress though that this research field is very active and new methods are regularly proposed that go beyond the simplified classifications proposed here. For instance, recent methods allow to infer complex demographic histories from full genomes (Steinrücken et al., 2019, Wang et al., 2020).

Here, we propose to use a different strategy based on the IICR (Inverse Instantaneous Coalescence Rate) introduced by Mazet et al. (2016). We propose to perform demographic inference under the piecewise stationary *n*-island model (Rodríguez et al., 2018), based on the symmetrical *n*-island model (Wright, 1931), using the IICR or estimates obtained from sequence data. The IICR, as defined by Mazet et al. (2016) for a sample of size two, is equivalent to the full distribution of coalescence times for that sample (i.e., the distribution of *T*_2_). Simulations by Chikhi et al. (2018) and Rodríguez et al. (2018) under various models of population structure suggest that the IICR is sensitive to population structure or fluctuations in migration rates (i.e., changes in connectivity).

The approach presented in the present study differs from the approaches mentioned above in several ways. First, we aim at *inferring* the number of populations rather than setting it a priori. Second, we ask whether it is possible to date and quantify changes in connectivity (i.e., gene flow) rather than changes in population size. For that, we use the piecewise stationary *n*-island model in which continuous gene flow happens between populations at a constant rate during specific periods (called *components*) but is allowed to change between periods (see below and Rodríguez et al. (2018)). This model differs from tree models in that we do not estimate parameters such as splitting times which may or may not be meaningful or appropriate for various species (Scerri et al., 2019) depending on their actual (unknown) demographic history. We acknowledge the limitations of the *n*-island model as it ignores spatial distances and other complexities of real species (Chikhi et al., 2018), but our choice for the current study is also guided by simplicity and computational considerations. We focus on changing patterns of connectivity since they may have been crucial in the recent evolutionary history of many species (Fenderson et al., 2020, Goldstein and Chikhi, 2002, Mazet et al., 2016, Quéméré et al., 2012, Salmona et al., 2017, Scerri et al., 2018, Steinrücken et al., 2019), particularly in the context of Pleistocene climate change and habitat fragmentation. Also, it is important to clarify whether the IICR contains useful information for parameter estimation and model choice (Mazet et al., 2016). The work presented here may thus represent an interesting endeavor, particularly given that there is an increasing recognition of ancient human structure by researchers of different fields (Scerri et al., 2019, 2018).

The inferential method is implemented in a program called SNIF (Structured Non-stationary Inferential Framework). We validated the method with data simulated under piecewise stationary *n*-island models and inferred *connectivity graphs* which are a visual representation of the times at which gene flow changed and of the magnitude of these changes. We then applied SNIF to human genomic data using five published PSMC curves (Prado-Martinez et al., 2013), allowing in each case the number of components to vary between analyses, and compared the inferred histories and connectivity graphs between individuals and with previously inferred scenarios by Rodríguez et al. (2018) and Noskova et al. (2019).

Beyond human data we find that a crucial issue is the estimation of the IICR from genomic data. Indeed, the stochasticity generated during the estimation of the IICR in very ancient times, and possibly recent times, with humps that are difficult to interpret, may lead to the inference of events that may never have taken place.

## Methods

To use the IICR as a summary of genomic information we first assume that an IICR curve can be obtained, which we will use as the *target* for demographic inference. With simulated data (sequences or *T*_2_ values) this target curve can be obtained under any predefined coalescent model that could be expressed with a simulation tool (e.g., the ms program (Hudson, 2002)). With real genome-wide sequence data, the curve can be estimated with the PSMC method of Li and Durbin (2011). We then try to identify a piecewise stationary *n*-island model that generates an IICR that is identical or similar to the target IICR (or PSMC curve). The similarity between the two IICR curves is quantified with a distance metric defined below. We use a genetic algorithm to explore the parameter space (number of populations, migration rate within a time component, and timing of these changes assuming a fixed number of components for each independent analysis) and minimize that distance. We compute the IICR under the non-stationary structured coalescent (NSSC) of Rodríguez et al. (2018).

### The structured coalescent and the IICR

The theoretical framework we use for modeling structure is based on the finite Herbots’s model of the structured coalescent (Herbots, 1994), which is a backwards-in-time view of the gene genealogies. We have *n* populations or demes that are assumed to behave as haploid Wright–Fisher models of size *N*_*i*_ = 2*s*_*i*_*N* genes, where *s*_*i*_ is the relative deme size and *N* is large. Migration occurs between demes as in each generation a proportion *q*_*ij*_ of lineages migrates from deme *i* to deme *j*. Herbots denoted by *m*_*i*_ the proportion of the population of deme *i* that was received from other demes in any given generation, such that *m*_*i*_ = ∑_*i*≠*j*_*q*_*ji*_*s*_*j*_/*s*_*i*_. She also showed that measuring time in units of 2*N* generations and making *N* go to infinity in such a way that the number of migrants stays bounded, the model converges to a continuous-time Markov process. It is possible to construct a transition rate matrix *Q* that provides a good approximation of the gene genealogies of the discrete time model. In this transformation, *q*_*ij*_ goes to zero in such a way that the product 2*Nq*_*ji*_*s*_*j*_/*s*_*i*_ converges and has limit *M*_*ij*_/2. Thus we can express the transition rates in *Q* in terms of *n*, *s*_*i*_, and *M*_*ij*_. In the case of the symmetrical island model (Wright, 1931), all the migration rates *M*_*ij*_ between any pair of islands *i* and *j* are equal, so we use the notation *M* = (*n* − 1)*M*_*ij*_ to denote the migration rate received by any given island. In addition to this base model, we use an extension, the NSSC, presented in Rodríguez et al. (2018) which allows to introduce demographic events that change the rate *M* or relative deme size *s* at certain points in time (see section “The piecewise stationary n-island coalescent”). We note however that throughout the manuscript we will only focus on symmetrical models with constant size (see section “Discussion” for extensions to symmetrical models with population size changes).

For the demographic histories under these models, we study the IICR of a sample of size 2 (see section S1.1 of the Supplementary Materials), and we use it as a statistic for demographic inference. We do this by comparing the IICR of many hypothetical demographic scenarios to a target IICR curve. This target IICR may be simulated, or it may be obtained from diploid individuals via full genome studies (Prado-Martinez et al., 2013). In such cases, these target IICRs are themselves inferred demographies under the assumptions of a particular model. For example, the PSMC method (Li and Durbin, 2011) uses the population size change model, where a single panmictic population varies in size according to a function *N*(*t*) = *N*(0)*λ*(*t*) (see Tavaré (2004)). It was shown by Mazet et al. (2016) that the IICR of a sample of size 2 under this model is exactly the *λ*(*t*) relative size changing function, and it relates to the distribution of the time to coalescence *T*_2_ as:

1 $$\begin{array}{lll}{\mathbb{P}}({T}_{2}> t)&=&\exp \mathop{\int }\nolimits_{0}^{t}\frac{-{\rm{d}}x}{\lambda (x)},\\ {\rm{IICR}}(t)&=&\lambda (t)=\frac{{\mathbb{P}}({T}_{2} > t)}{{f}_{{T}_{2}}(t)}.\end{array}$$

The IICR is not tied to any particular model, structured or otherwise. It is defined using the distribution of the coalescent times of a sample of size two. It can be approximated to arbitrary numerical precision under the assumptions of the NSSC (Rodríguez et al., 2018); it can also be computed empirically by simulating a sample of coalescent times (Chikhi et al., 2018); or it can be read from full sequence genomic data using the appropriate methods (e.g., Li and Durbin (2011)).

### The piecewise stationary n-island coalescent

#### The parameter space

We first define the parameter space, as this directly determines the family of demographic histories that we can explore and infer from. The piecewise stationarity refers to the fact that, although migration rate is constant and identical between any pair of islands, this rate may be different between consecutive time periods (components), and there is a fixed number *γ* that represents the number of demographic events. To say that there are *γ* ⩾ 0 changes of gene flow thus means that there are *c* = *γ* + 1 components or periods of constant gene flow. Likewise, the deme size, which is the same for all islands, may in theory change through time in the general model presented in Rodríguez et al. (2018). In the present study we focus on models with constant population size but we present a more general model where deme sizes can change between components. In this more general case, the parameter space includes the number of islands *n*, the times *t*_*i*_ for the demographic events, and the values of both the migration rate *M*_*i*_ and the local deme size *s*_*i*_ at each new demographic period. Note that *n* is inferred but it does not change through time. We thus assume no extinction, no population split, and no creation of new populations.

Given a fixed integer *γ* of demographic events to consider (*γ* ⩾ 0) and a collection of bounds *B* in the form of:

2 $$\begin{array}{lll}B=\left([{n}_{\min };{n}_{\max }],[{t}_{1\min };{t}_{1\max }]\ldots [{t}_{\gamma \min };{t}_{\gamma \max }],\right.\\ \left.[{M}_{0\min };{M}_{0\max }]\ldots [{M}_{\gamma \min };{M}_{\gamma \max }],[{s}_{0\min };{s}_{0\max }]\ldots [{s}_{\gamma \min };{s}_{\gamma \max }]\right),\end{array}$$

we define the parameter space Φ_*γ*,*B*_ as:

3 $$\begin{array}{lll}{{{\upphi }}}_{\gamma ,B}=\left\{\varphi =(n,\,{t}_{1}\ldots {t}_{\gamma },{M}_{0}\ldots {M}_{\gamma },{s}_{0}\ldots {s}_{\gamma })\in {\mathbb{N}}\times {{\mathbb{R}}}^{3\gamma +3},\,\text{s.t.}\,\forall i:\right.\\ 2\leqslant {n}_{\min }\leqslant n\leqslant {n}_{\max };\quad 0<{t}_{i\min }\leqslant {t}_{i}\leqslant {t}_{i\max };\\ \left.0<{M}_{i\min }\leqslant {M}_{i}\leqslant {M}_{i\max };\quad 0<{s}_{i\min }\leqslant {s}_{i}\leqslant {s}_{i\max }\right\}.\end{array}$$

We define bounds for each variable because we use a constrained optimization algorithm, for which all parameters must be bounded (see section “Optimization framework: search algorithm and optimality criteria”). Also, since we focus on the case where there are no deme size changes, we enforce this by using *B*, as making ${s}_{i\min }={s}_{i\max }=1$ for all 0 ⩽ *i* ⩽ *γ* effectively fixes all deme sizes to 1, and reduces the number of parameters to 2*γ* + 2.

#### Computing and scaling the IICR

Given any demographic scenario from Φ_*γ*,*B*_, the associated coalescent process is an instance of the NSSC of Rodríguez et al. (2018). Our main object of interest regarding these scenarios is the IICR. In the Supplementary Materials we provide a brief overview of how to perform its computation for any given *φ* ∈ Φ_*γ*,*B*_ based on the work of Rodríguez et al. (2018).

The computation of the IICR uses functions that receive the time *t* in units of 2*N* generations, and return values in units of *N* generations per coalescence, so these IICR functions are dimensionless in the sense that they operate in a *relative* frame of reference.

In order to compare the IICR with PSMC inferences, we need to re-scale both the time and the IICR values by a reference deme of size *N* which specifies how many haploid genes correspond to a local deme size of 1 as follows:

$${\rm{sIICR}}(g)=N \cdot {\rm{IICR}}(g/2N);$$

where sIICR(*g*) refers to the *scaled* IICR, and IICR(*t*) to the *unscaled* (dimensionless) one. Note that we use *g* for generations as the variable name for sIICR to further stress the difference. The parameter space for the sIICR can be thought of as a simple one-dimensional addition to Φ_*γ*,*B*_:

$${\hat{{{\upphi }}}}_{\gamma ,B}=\left\{\hat{\varphi }=(N,\varphi )\in {\mathbb{R}}\times {{{\upphi }}}_{\gamma ,B}\,{\mathrm{such}}\, {\mathrm{that}}\,0<{N}_{\min }\leqslant N\leqslant {N}_{\max }\right\}.$$

In section S1.2 of the Supplementary Materials, we present the piecewise-continuous version of this parameter space for both the scaled and unscaled IICRs, as well as the relationship between them.

### Optimization framework: search algorithm and optimality criteria

The search algorithm explores the parameter space and uses an optimality criterion to select the structured scenario that best explains a given target IICR curve, either scaled or unscaled. We also assume that the underlying coalescence times for these target IICRs have cumulative distribution *F*_0_ and density *f*_0_.

Given a target IICR_0_ and a parameter space Φ_*γ*,*B*_, we want to find a parameter tuple *φ* in Φ_*γ*,*B*_ such that the exact IICR curve corresponding to the model defined by *φ* (denoted by IICR_*φ*_) approximates IICR_0_. We thus want to find the minimal distance:

4 $$\mathop{\min }\limits_{\varphi \in {{{\Phi }}}_{\gamma ,B}}d\left({{\rm{IICR}}}_{0},{{\rm{IICR}}}_{\varphi }\right).$$

Regarding the distance *d*, a straightforward definition would be:

5 $$d\left({{\rm{IICR}}}_{0},{{\rm{IICR}}}_{\varphi }\right)=\mathop{\int }\nolimits_{0}^{\infty }\left|{{\rm{IICR}}}_{0}(t)-{{\rm{IICR}}}_{\varphi }(t)\right|w(t){\rm{d}}t,$$

where *w*(*t*) is a weight function that should take into account the natural distribution of the information in an IICR. One reasonable solution for *w* is to take a quantity proportional to the density *f*_0_ of the coalescence times because it ensures that the integral in (5) is finite, and also because it assigns more weight to the temporal periods where the target IICR_0_ is expected to be more accurate and reliable since more coalescences are likely to have happened.

We thus consider the family of weight functions:

6 $$w(t)=\frac{{f}_{0}^{\omega }(t)}{\parallel {f}_{0}^{\omega }{\parallel }_{1}},$$

where ∥ ⋅ ∥_1_ is the *L*^1^-norm and *ω* > 0 is a *weight-shifting* parameter, with the purpose of dampening (if *ω* < 1) or exaggerating (if *ω* > 1) the effect of the weight *f*_0_. Unless otherwise noted, we take *ω* = 1, which corresponds in practice to giving more weight to recent periods of the IICR in direct proportion to the density *f*_0_ in an *n*-island model.

In practice, we need to consider that all we know about IICR_0_ is a stepwise discretization over a bounded interval of time, so a numerical approximation of the distance (5) is required. This includes approximating the density *f*_0_ of the underlying *T*_2_ distribution. Given a division of time into ${\mathcal{I}}$ intervals $\left\{[{\tau }_{j-1};{\tau }_{j})\right\}$ for $1\,{\leqslant}\, j\,{\leqslant}\, {\mathcal{I}}$, where *τ*_0_ = 0 and ${\tau }_{{\mathcal{I}}}\,<\,\infty$, we can consider a discrete representation of IICR_0_ in the form of a collection of ${\mathcal{I}}$ values {*y*_*j*_} such that:

7 $${{\rm{IICR}}}_{0}(t)={y}_{j},\quad \forall t\in [{\tau }_{j-1};{\tau }_{j}),\,1\,{\leqslant}\, j\,{\leqslant}\, {\mathcal{I}}.$$

We can use this form to compute a numerical approximation for the integral in (5). For instance, a first degree approximation would yield:

$$d\left({{\rm{IICR}}}_{0},{{\rm{IICR}}}_{\varphi }\right)=\mathop{\sum }\limits_{j=1}^{{\mathcal{I}}}\left|{y}_{j}-{{\rm{IICR}}}_{\varphi }({\tau }_{j})\right|\ w({\tau }_{j-1})\ ({\tau }_{j}-{\tau }_{j-1}).$$

As for the values of *w*(*τ*_*j*_), notice that from (1) we have the identity:

$${f}_{0}(t)=\exp \left({\int\nolimits_{0}^{t}}\frac{-{\rm{d}}\tau }{{{\rm{IICR}}}_{0}(\tau )}\right)/{{\rm{IICR}}}_{0}(t),$$

which, using the representation (7), can be discretized into:

$$\begin{array}{lll}{f}_{0}(0)=1/{y}_{0},\\ {f}_{0}({\tau }_{j})=\exp \left(\mathop{\sum }\limits_{k=1}^{j}\frac{{\tau }_{k-1}-{\tau }_{k}}{{y}_{k}}\right)/{y}_{j},\quad 1\,{\leqslant}\, j\,{\leqslant}\, {\mathcal{I}}.\end{array}$$

We then have that for any given *ω*, *w*(*τ*_*j*_) can be expressed as:

$$w({\tau }_{j})={f}_{0}^{\omega }({\tau }_{j})/\mathop{\sum }\limits_{k=1}^{{\mathcal{I}}}{f}_{0}^{\omega }({\tau }_{k}).$$

An alternative option for the definition of *d* in (4) could be one that takes into account the ultimate visual nature of the curve fitting task. Assuming that the points {*τ*_*j*_} are log-distributed and that they will be used for visualization purposes in a horizontally log-scaled plot like Fig. S37, then the definition:

8 $$d({{\rm{IICR}}}_{0},{{\rm{IICR}}}_{\varphi })=\mathop{\sum }\limits_{j=1}^{{\mathcal{I}}}\left|{y}_{j}-{{\rm{IICR}}}_{\varphi }({\tau }_{j})\right|,$$

captures the perceived visual difference between the plots of the two curves. We distinguish distance (5) from (8) by denoting them *d*_*ω*_ and *d*_visual_ respectively. We keep both definitions because we found that the weighted family of distances generally performs better than the visual distance under certain validation tests, but also that the *d*_visual_ distance can be used to choose the optimal weight parameter in *d*_*ω*_ (see Fig. S46).

Regarding the optimization problem (4) itself, we use the *Differential Evolution* method (Storn and Price, 1997). As a genetic meta-heuristics, this algorithm maintains and evolves (using mutation and recombination parameters) a population of solutions iteratively. As a global optimization algorithm, it features mechanisms for escaping local *optima* of the parameter space. In section S2.3 of the Supplementary Materials, we explore the potential effects on the inference results of tuning some of the parameters provided by this algorithms implementation. For our validations, the method runs by using multiple steps of optimization which we refer to as *rounds*. In addition, we stress that the method should be used multiple times on real data sets. We set a maximum number of allowed rounds, as well as a tolerance *ε* for the distance which controls the minimum number of rounds.

### Validation framework

**Fig. 1:**
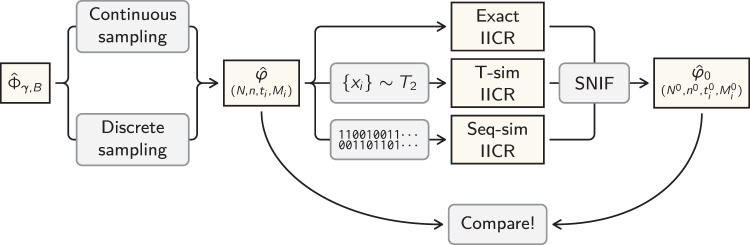
Flowchart of the validation procedures. Starting from a parameter space ${\hat{{{\Phi }}}}_{\gamma ,B}$ we use one of two sampling methods (section “Sampling the parameter space”) to generate a demographic history $\hat{\varphi }$ defined (for the scaled case) by the parameters (*N*, *n*, *t*_*i*_, *M*_*i*_). We then compute the IICR of that demographic history using one of three methods (section “The three types of target IICRs”) to obtain the target IICR. After that, we run the inference algorithm on this target IICR curve (using wider bounds than those in *B*) to obtain an estimated (or inferred) demographic history ${\hat{\varphi }}_{0}=({N}^{0},{n}^{0},{t}_{i}^{0},{M}_{i}^{0})$, which we then compare to the known $\hat{\varphi }$ in order to assess the accuracy of the inference methodology (section “Results”).

We applied the SNIF inferential method to target IICRs generated under piecewise stationary *n*-island models of increasing complexity (i.e., number of components) and with known parameter values (*N*, *n*, *t*_*i*_, *M*_*i*_) and then compared the inferred parameter values to those actually used (see Fig. 1).

In what follows we present various ways of generating random demographic scenarios and then computing appropriate IICR curves from them for use in validation.

#### Sampling the parameter space

Given a parameter space Φ_*γ*,*B*_ (we only discuss the unscaled case here for brevity, but the same principles apply to a scaled parameter space), we sample demographic scenarios from which we compute the corresponding IICRs. We used two sampling strategies which we call continuous and discrete sampling.

##### Continuous sampling

Assuming that we want to realize *L* independent tests, this sampling strategy consists in using uniform or log-uniform distributions for each of the 3*γ* + 3 random variables:

9 $$\begin{array}{lll}n& \sim &U\{{n}_{\min },{n}_{\min }+1,\ldots ,{n}_{\max }\},\\ {t}_{1}& \sim &L{U}_{10}[{t}_{1\min };{t}_{1\max }],\ldots ,{t}_{\gamma } \sim L{U}_{10}[{t}_{\gamma \min };{t}_{\gamma \max }],\\ {M}_{0}& \sim &U[{M}_{0\min };{M}_{0\max }],\ldots ,{M}_{\gamma } \sim U[{M}_{\gamma \min };{M}_{\gamma \max }],\\ {s}_{0}& \sim &U[{s}_{0\min };{s}_{0\max }],\ldots ,{s}_{\gamma } \sim U[{s}_{\gamma \min };{M}_{\gamma \max }],\end{array}$$

where *U* denotes a uniform distribution (discrete in the case of *n* and continuous for the rest) and *LU*_10_ denotes a log-uniform distribution of base 10. This distribution is used for sampling the times of changes in a logarithmic space in order to take into account the natural distribution of information in an IICR.

After sorting the times *t*_*i*_, we can define the *L* sampled scenarios by constructing, for 1 ⩽ *j* ⩽ *L*, the tuple $\left({n}^{j},{t}_{1}^{j}\ldots {t}_{\gamma }^{j},{M}_{0}^{j}\ldots {M}_{\gamma }^{j},{s}_{0}^{j}\ldots {s}_{\gamma }^{j}\right)$. This sampling strategy makes it very unlikely to sample exactly the same parameter values twice or to sample exactly the same *M*_*i*_ values in two consecutive components. However, it sometimes produces demographic scenarios in which consecutive *t*_*i*_ and/or *M*_*i*_ values may be close to each other, and thus difficult to distinguish. This makes it thus harder on our inferential framework compared to cases where we would choose contrasted scenarios with clearly separated events with major changes in migration rates. In other words, our inferential method was sometimes inferring parameters in the case of extremely difficult scenarios as we show below.

In section “Validation” we show the results obtained using this sampling method with *L* = 400 scenarios. The bounds for sampling and inferring are shown in Table S1. In particular, we note that in practice we disallow deme size changes by fixing the bounds of the *s*_*i*_ to 1, which consequently reduces the parameter space to just 2*γ* + 2 parameters.

##### Discrete sampling

Here we sampled *L* = 100 independent scenarios from the same parameter space, but using the following set of predefined values:

$$\begin{array}{lll}n\in {\boldsymbol{n}}=\{2,5,10,15,20\},\\ {t}_{i}\in {\boldsymbol{t}}=\{1/10,1/2,1,2,5,10,20,50\}\quad \forall i,\\ {M}_{i}\in {\boldsymbol{M}}=\{1/10,1/5,1/2,1,2,5,10,20,50\}\quad \forall i,\\ {s}_{i}\in {\boldsymbol{s}}=\{1\}\quad \forall i;\end{array}$$

The inference process was, however, done within the continuous space. For instance, under this validation scheme (see section “Validation using T-sim IICRs”) we only simulated data with 2, 5, 10, 15, 20 islands but the inference process always allowed *n* to take any value between 2 and 50. The choice of the *L* independent simulated data sets was done using the following procedure. We first considered the following cartesian product of dimension 2*γ* + 2:

$$K={\boldsymbol{n}}\times {{\boldsymbol{t}}}^{\gamma }\times {{\boldsymbol{M}}}^{\gamma +1}\times \{1\}.$$

and then uniformly drew *L* tuples from *K* without replacement. We then sorted the sampled event times obtaining thus a set of *L* demographic scenarios. We drew randomly (without replacement) from the set *K*, rejecting scenarios with identical *M*_*i*_ values in two consecutive components, until we reached *L* accepted scenarios.

#### The three types of target IICRs

We explored three different types of target IICRs (see Fig. S3) that could be obtained given a scenario *φ* ∈ Φ_*γ*,*B*_. All IICRs were discretized so as to be comparable to PSMC plots (see eq. (7)).

Validating SNIF on PSMC plots across the parameter space described above would be extremely time consuming as it would require simulating genomes and then running the PSMC method (or other related methods) on these genomes before applying our approach. We thus only ran the PSMC method in the case of the scenarios inferred for the human data so as to integrate the uncertainty due to the PSMC inferential process. The issue of uncertainty is crucial but our aim is not to test the PSMC or other inferential methods. To clarify this we explain below the different types of IICR that could be computed given a scenario *φ* ∈ Φ_*γ*,*B*_ (see also Fig. 1).

##### Exact IICR

We can compute the IICR for any *n*-island model at any time value *t*, but to generate input data we need a discretization as in (7), so considering that we take a log-distributed sample of size *I* in the interval $[{t}_{\min },{t}_{\max }]$, we end up with a suitable IICR_0_. Note that even though this IICR has been discretized, its values are exact within machine precision, so it is still an artificial product compared to real data.

For the validations using the exact IICR in section “Validation using exact target IICRs” we chose for the distance tolerance between a target and an inferred IICR a value of *ε* = 10^−10^ for the unscaled IICRs and an equivalent value of *ε* = 10^−7^ for the scaled IICR (since the simulated *N* was always 1000). It should be noted that this value of *ε* is quite small even for double-precision floating-point arithmetic, and thus is only a reasonable choice for validation using exact IICRs (i.e., those where the distance could theoretically be zero).

##### T-sim IICR

The T-sim IICR is obtained by simulating a finite collection of *T*_2_ realizations using ms and then building an empirical IICR as in Mazet et al. (2016), using the Kaplan–Meier estimator (Kaplan and Meier, 1958), with log-distributed times. We stress that ms scales time in units of 4*N* generations whereas our models use a scale of 2*N* generations (see the example in Fig. S3), so this must be kept in mind when writing ms commands.

##### Seq-sim IICR

We simulate genomic sequences with ms and then apply the PSMC method for obtaining the IICR_0_ to be used by the inference method. Since simulating genomes and performing PSMC analyses is significantly more time consuming than the other two methods, we limited ourselves to validating the Seq-sim IICR for the human PSMC based scenarios that we obtain after performing the demographic inference described in section “Application to humans”. The results of this step are shown in section S5.1 of the Supplementary Materials.

### Application to humans

We applied our method to the human genomes published in the great apes study by Prado-Martinez et al. (2013). Namely, we used the PSMC files of five sampled humans identified as Dai, French, Karitiana, Sardinian, and Yoruba (see Fig. S37). For each human PSMC curve we performed demographic inference independently within the following bounds:

10 $$\begin{array}{lll}n&\in &\{2,3,\ldots ,100\};\\ {t}_{i}&\in &[4\times 1{0}^{2},4\times 1{0}^{5}]\quad \forall i;\\ {M}_{i}&\in &[1/20,20]\quad \forall i;\\ {s}_{i}&=&1\quad \forall i;\\ N&\in &[1{0}^{2},1{0}^{4}].\end{array}$$

The bounds for the *t*_*i*_ are specified in generations, so given a generation time of 25 years, we effectively allowed for the inference of demographic events between 10 thousand and 10 million years ago. Regarding the number of components, we choose *c* ∈ {2, 3, 4, 5}; i.e., between one and four demographic events in agreement with Mazet et al. (2016) who suggested that a minimum of three events were necessary to explain the two humps, and in agreement with our validation simulations which suggest that inference above five components is difficult.

Some of the analyzed PSMC curves exhibit an increase in effective size in the recent past, which could be due to a genuine population growth as noted by Mazet et al. (2016). Given that we choose to specifically rule out changes in deme sizes, we account for this fact by running every inference a second time, ignoring this period of possible recent expansion. This is accomplished using an option that allows to limit the interval where the distance function is computed. In this case, we restricted both this range and the bounds for the *t*_*i*_ to be between 50 thousand and 10 million years ago, thus ignoring any population size change that may have happened in the last 50,000 years. Note that this option is also useful to ignore very ancient sections of the PSMC plots which may be difficult to trust.

Since real human PSMCs are unlikely to have been generated by an n-island model, the default value of *ω* used for simulated data may not be the most appropriate, and we thus performed inferences with *ω* ∈ {1, 0.5, 0.2}. Decreasing values of *ω* give increasing weight to the most ancient part of the PSMC (see the weighted distances (6)). The resulting inferred demographic scenarios are shown in section “Application to humans”.

To validate the inference process using PSMC outputs, we generated 10 Seq-sim IICRs corresponding to the inferred demographic scenarios for the French, Karitiana, and Yoruba individuals. We exclude the Dai and Sardinian populations from this analysis because their corresponding inferred histories are similar to the other three (see Figs. S40 through S46). For each one of the selected scenarios we simulated nreps = 30 chromosomes of length *L* = 10^8^ base pairs, using the effective size *N* inferred by SNIF, a per-base per-generation mutation rate of *μ* = 1.25 × 10^−8^ (see (Scally and Durbin, 2012) and references therein). We kept for consistency the scaled recombination rate of *ρ* = *θ*/5 as in Li and Durbin (2011), and we ran the ms command with *θ* = 4*μLN* using:

ms 2 nreps -t *θ* -r *ρ* L -p 8 -I …

where the rest of the command follows according to the inferred demography (see Fig. S3 for a reference). After that we prepared a *.psmcfa* file as input for PSMC, always choosing a bin size of *s* = 100. Then we ran the PSMC with the command:

psmc -N25 -t15 -r5 -p "4+25*2+4+6" …

following Li and Durbin (2011) on human data. We then scaled the resulting curve using the information in the generated *.psmc* file and used these PSMC curves as targets to determine whether we could indeed infer the parameters used for such complex scenarios.

We also applied SNIF to genomic data simulated under the scenarios used to describe recent human evolutionary history by Gutenkunst et al. (2009) and Noskova et al. (2019). Here, we thus ask the following two questions: if human evolution were indeed closer to such splitting models, would our method infer again an *n*-island model with similar parameters to those inferred from the humans PSMCs? additionally, do these models generate IICR plots that are similar to the human PSMCs? The results of these validations are shown in section S5.1 of the Supplementary Materials.

## Results

In this section we show the results of validating SNIF using target IICRs from known demographic histories; the application of the method to real human data; and the comparison of the obtained results with previously published demographic histories for humans.

The results of the validations are presented in Figs. 2–4 in the main manuscript and Figs. S6–S36 in the Supplementary Materials. Another set of figures (Figs. 5–8 and Figs. S40–S46) present the results of the application to human data.

**Fig. 2:**
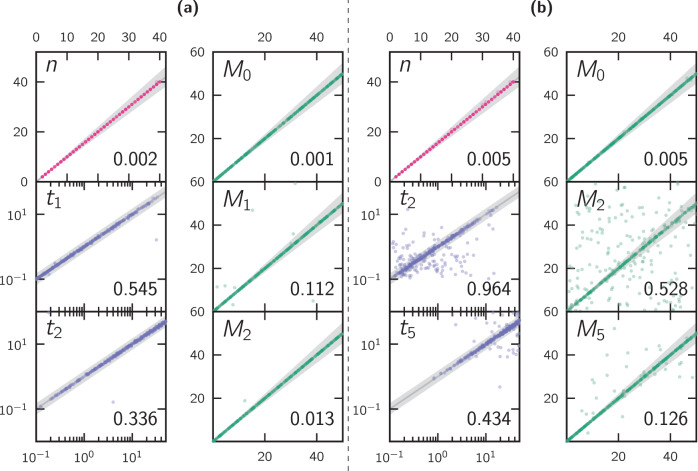
Scatter plots of simulated and inferred parameters. **a** Corresponds to scenarios with *c* = 3 components, and **b** to scenarios with *c* = 6 components. The different sub-panels represent the simulated (horizontal axis) versus inferred (vertical axis) parameter values for all the parameters (or a representative selection of parameters in the case of **b**) of *L* = 400 unscaled simulated scenarios.

**Fig. 3:**
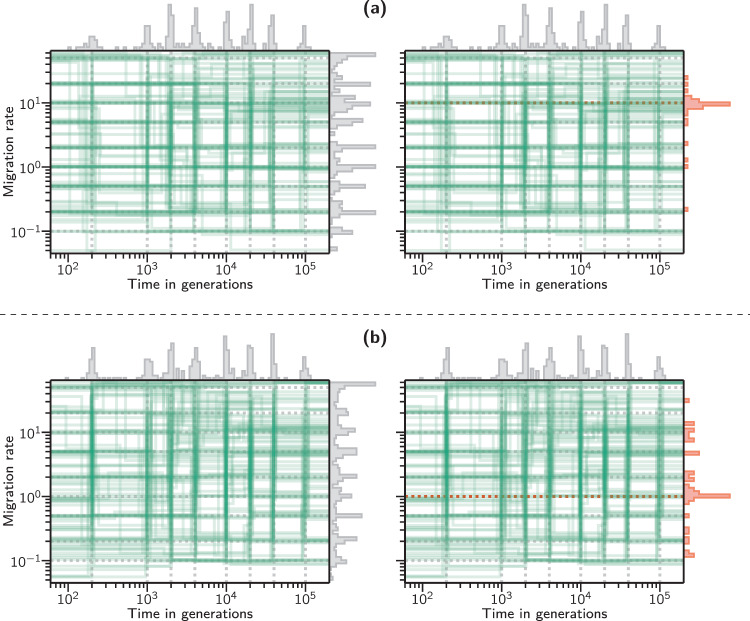
Connectivity graphs of 100 independently inferred histories obtained by sampling for each scenario from the values indicated by the dotted lines. **a** Scenarios with *c* = 3 components. **b** Scenarios with *c* = 4 components. The right sub-panels show a side histogram with only the inferred migration rates for those components with a specific simulated migration rate (10 for (**a**) and 1 for (**b**)).

**Fig. 4:**
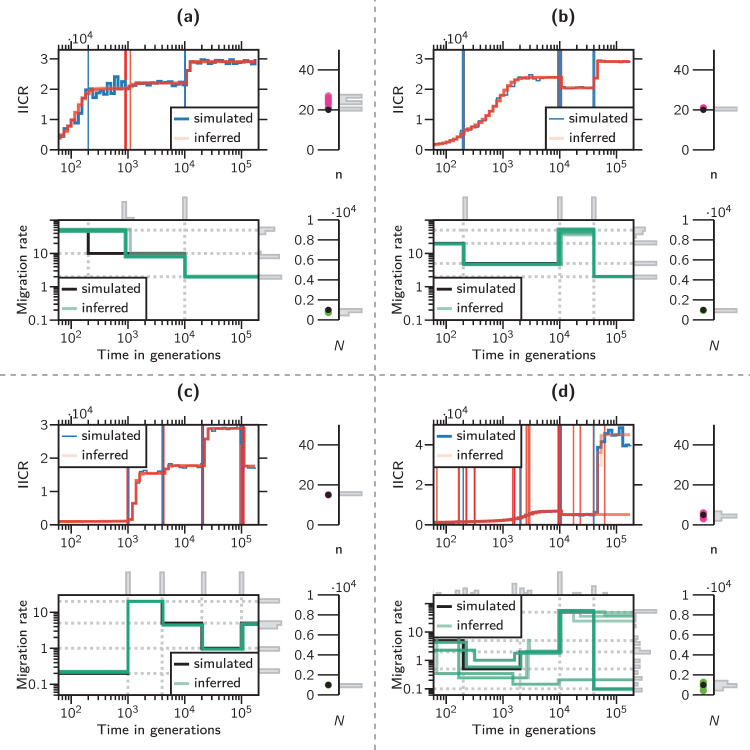
Simulated and inferred IICR plots, connectivity graphs, *N* and *n*. The four panels correspond to four different scenarios. **a** A *c* = 3 components scenario. **b** A *c* = 4 components scenario. **c** A *c* = 5 components scenario. **d** A *c* = 5 components scenario. The left part of each panel represents the target and inferred IICRs (top), and the connectivity graphs (down). The right half of each panel shows the simulated and inferred values for *n* (top) and *N* (down). In each IICR graph, the ragged blue line represents the target IICR whereas the red lines represent 10 independently inferred IICRs. The vertical blue and red lines are located at the simulated and inferred values of the event times *t*_*i*_, respectively. In the connectivity graphs, the black and green lines represent the simulated and inferred connectivity scenarios, respectively. The simulated *n* and *N* values are represented by black circles whereas the inferred values for the corresponding parameters are represented by red and green full circles and by gray histogram bars.

**Fig. 5:**
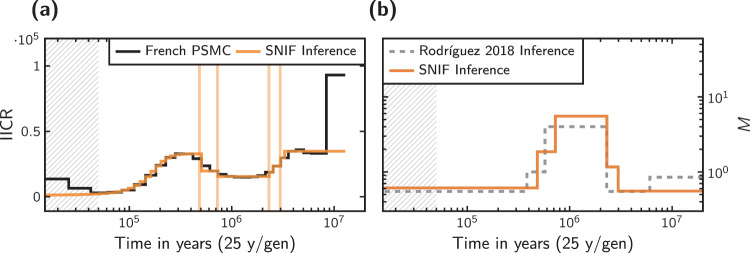
Results of performing demographic inference on the French PSMC curve. **a** Shows the IICR plot inferred for *c* = 5 components and a weight parameter of *ω* = 0.2. The vertical lines represent the inferred times of the demographic events. **b** Shows the connectivity graph for the same inferred scenario. As a reference point, the connectivity graph of the scenario proposed in Rodríguez et al. (2018) is also shown. The vertical axis in **b** represent migration rates (*M*).

**Fig. 6:**
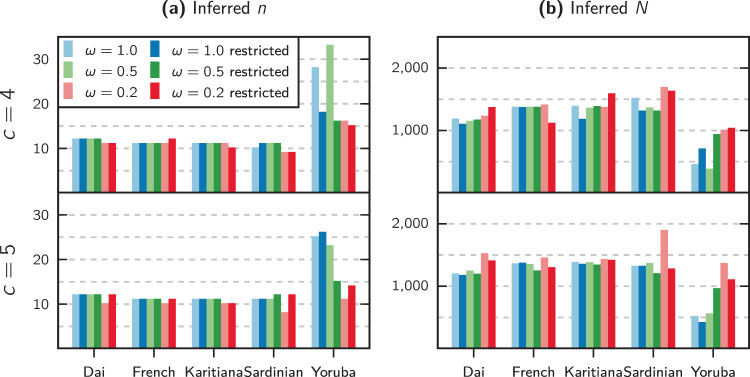
Results of performing demographic inference on the human PSMC curves. **a** Shows the inferred number of islands *n* and **b** the inferred reference sizes *N* for each human population and each used combination of the weight parameter *ω* and number of components *c* (only 4 and 5 are shown here). The bars with the darker color, marked ’restricted’ in the legend, correspond to inferences realized with the option of ignoring recent population expansion.

**Fig. 7:**
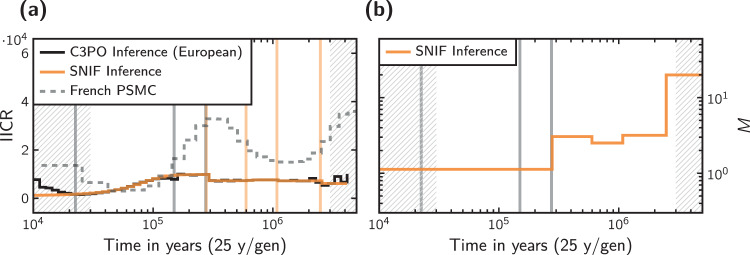
Application of our inference method to a tree-like human demographic scenario with three modern populations. **a** IICR plots showing the resulting IICR curve of the European population under this model and the inferred IICR curve obtained with our method (where the recent period of human expansion was ignored) for *c* = 5 components and a weight parameter of *ω* = 0.25. For reference purposes, we also show the real PSMC curve of the French individual. The gray vertical lines indicate the inferred event times in the C3PO model, and the colored vertical lines the inferred event times by SNIF. **b** Connectivity graph of the inferred scenario. For reference, we show the inferred event times in the C3PO model as gray vertical lines.

**Fig. 8:**
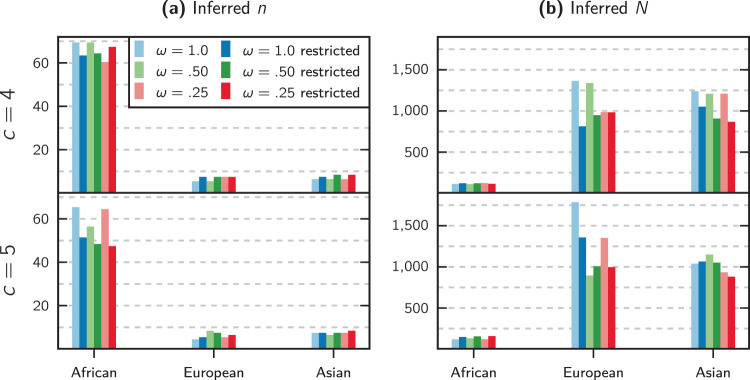
Application of our inference method to a generally accepted tree-like human demographic scenario with three modern populations. **a** Inferred number of islands for each modern population. **b** Inferred local size of each island. Shown here are the scenarios with 4 and 5 components *c*, and all three values of the weight-shifting parameter *ω*. The bars with the darker color, marked ‘restricted’ in the legend, correspond to inferences realized with the option of ignoring recent population expansion.

### Validation using exact target IICRs

A first set of figures (Fig. 2, Figs. S6–S11 and S13–S18) represents the simulated and inferred parameter values on the horizontal and vertical axes, respectively, using the continuous sampling strategy. As explained in section “Sampling the parameter space”, the range of possible values in the inference process was always wider than the range used for the simulated values (see Table S1 for the exact values). We quantified the inference error for each parameter by computing the Normalized Root-Mean-Square Deviation (nRMSD). This value is displayed in the lower-right corner of each panel of the previously mentioned figures, and summarized for all parameters in Fig. S20. For reference, we also highlight the *y* = *x* line, indicating what would be a perfect inference, and the region corresponding to 10% of relative error around this line (50% of relative error in the case of the *t*_*i*_ parameters). A summary of how many tests fall within this margin of error (and others) is displayed in Figs. S12 and S19. Altogether, these figures always show the data points near the *y* = *x*, hence suggesting that the inferred parameter was identical or very close to the simulated parameters. This is particularly obvious for all the parameters corresponding to scenarios with up to four components, where the nRMSD values stayed below 0.5 (the case of the *t*_*i*_ parameters is exceptional, since the exponential distribution of its range disproportionately affects the error measures). For instance, Fig. 2a shows the results for a model with three components, in which there is a nearly perfect match (nRMSD close to or below 0.1 for the non-*t* parameters) between the simulated and inferred values for the model parameters. For five- and six-component scenarios the results are still nearly perfect for most of the simulated scenarios but we observe an increasing number of cases (i.e., simulated scenarios) where the parameters are poorly estimated, with the exception of *n*, *M*_0_ (and *N* for scenarios with scaled IICRs) which are almost always well estimated also in such cases (nRMSD values consistently close to or below 0.1). In particular, we can appreciate a gradual degradation of the correspondence between simulated and inferred migration values when the number of components *c* increases, as the nRMSD monotonically increases to over 0.5 for *c* = 6. These cases can be identified by the dots scattered in the different panels. They start to appear in scenarios with three components, but their number grows with the number of components.

These poorly estimated parameters are surprising given the near perfect estimation obtained for most parameter combinations. This is particularly striking because these dots do not seem to be distributed in any clear area of the parameter space. We see at least two possible and nonexclusive interpretations for this result. One is that the search algorithm had not yet *converged* when the maximum number of rounds was reached.

The maximum number of rounds was set to 500 in all simulations because we had found that less than 50 rounds were more than enough in the first tests carried out with one or two components. The search algorithm might however need more than 500 rounds to reach the optimal solution for scenarios of increasing complexity. We thus asked whether the maximum the number of rounds had been reached in the scenarios analysed and whether the proportion of scenarios with 500 rounds increased with the number of components. We found indeed that the proportion of simulations for which that maximum was reached increased with the number of components. For instance, all five- and six-component scenarios stopped their parameter search at 500 rounds, hence suggesting that at least some had not yet reached an optimum solution. For the cases with one- and two-component scenarios, all 800 independent simulations reached convergence in less than 150 rounds (see Fig. S4). Again, the choice of the tolerance *ε* plays a role in these results, and selecting larger tolerances will tend to produce earlier convergence in general, but not necessarily better results.

As a test we randomly identified a couple of scenarios with six components that had bad estimates and re-ran the algorithm with 5000 rounds. We found that the distance value consistently decreases with more rounds (see Fig. S5), but the inferred parameter values may not converge to the simulated ones because with more components there is a higher probability that two consecutive simulated *M*_*i*_ values are very close, thus making the corresponding time of the event challenging to infer.

The second possible reason for the poorly estimated parameters in Fig. 2 may be related to the fact that some simulated components may have a short duration that do not leave a significant mark on the IICR curve, thus leading them to be “skipped”. We refer to this issue as component misidentification or misassignment, which could lead to a particular estimated parameter to be plotted in the wrong panel. For instance, the method may miss the first change in migration rate at *t*_1_ and identify the second change in migration at *t*_2_. In such a case the method will assign the inferred *t*_2_ value to the set of inferred *t*_1_ values and plot it in the *t*_1_ panel. This wrongly assigned *t*_2_ value will thus appear away from the diagonal in the *t*_1_ panel even if it was well-estimated. Such misassignment cases for one parameter will also have consequences for the *M*_*i*_ plots, and thus will generate several misassignments across panels. They are also expected to increase in frequency as the number of components increases and as the *t*_*i*_ values become closer to each other. This phenomenon can be observed clearly in the right panels of Fig. 3. We also present an attempt at quantifying it for the case of *c* = 5 in Fig. S21. One way to mitigate the effect of this misassignment issue in the analysis of the results is to visualize the simulated and inferred scenarios using what we call a connectivity graph. This connectivity graph represents the times at which migration changes against the values of the migration rates. Such connectivity graphs are featured in the next section.

### Validation using T-sim IICRs

The connectivity graphs and IICR plots obtained from simulated *T*_2_ values show that again the scenarios are generally very well reconstructed (Fig. 3 and Figs. S22–S36).

In Fig. 3 the connectivity graphs obtained for all the scenarios simulated with three and four components show that the inferred times at which migration rates changed (green vertical lines) and the inferred migration rates (green horizontal lines) are generally overlapping close to the simulated values (dotted vertical and horizontal gray lines). In the right panels of this figure, we show a subset of the inferred migration histogram (in red). Namely, we show the distributions of the migration values that were inferred for components with a simulated migration value of *M*_*i*_ = 10 for panel (a) and *M*_*i*_ = 1 for panel (b). This allows us to better appreciate the variance of the inferred migration values in the context of the simulated ones, as well as the component misassignment effect mentioned earlier. Indeed, we note here that the incorrectly inferred migration values are clustered around other simulated values, indicating a mismatch in a particular component assignment which does not affect the rest of the inferred demographic history (we present a quantification of this effect for a particular case in Fig. S21).

For example, consider the right sub-panel of (a). We see that most repetitions correctly inferred a value close to *M* = 10 for the components with that simulated migration rate. However, there were cases where a given component *i* was simulated with a migration rate of *M*_*i*_ = 10, but it was missed entirely (maybe because it did not generate a very different IICR or because it had a short duration), and thus the inferred migration value for component *i* ultimately reflected either *M*_*i*−1_ or *M*_*i*+1_. In panel (b) we can observe the same effect with higher intensity because with more components it is more likely for them to be misassigned or misidentified during inference. See Fig. S21 for a quantification of this effect on scenarios of *c* = 5 components.

These connectivity graphs (and the one obtained for five components shown in Fig. S34) also show that there are regions of the parameter space where the green lines are more widely distributed. For instance, in the recent past of Fig. 3b (*t*_*i*_ < 10^−3^ generations) when the simulated *M*_*i*_ value was 0.1 or 0.2 the inferred values seem to vary between 0.05 and 0.3, suggesting that the method identifies periods with low migration rates but that the exact value is difficult to estimate properly, at least in the recent times. These graphs however summarize extremely different scenarios, including scenarios in which consecutive *M*_*i*_ values may be similar. We thus stress that the quality of the inference is dependent on the timing of the changes in migration rates and on the size of the change in *M*_*i*_ values.

Figure 4 shows the results for four different scenarios. In each of the four panels, we represented the inferred and target IICR plots, connectivity graphs, *N* (the size of each the islands) and *n* (the number of islands) for the corresponding model. Panels (a) and (b) correspond to three- and four-component scenarios, whereas panels (c) and (d) show the results for two five-component scenarios, one for which we obtained very good estimates and one for which the estimates were poorer. In panels (b) and (c) the inferred and simulated *M*_*i*_ and *t*_*i*_ values are on top of each other as can be seen in the connectivity graphs. Similarly, *N* and *n* are also well estimated. Here, the IICR plots also overlap, although this does not always guarantee perfect parameter estimation, as is the case in panels (a) and (d). Interestingly, in panel (a) the first change in migration rate (at *t*_1_ = 200 generations) is estimated at around 900 generations due to the stochasticity of the IICR plot. This appears to generate some variance in the estimates of *N* and *n* but the connectivity graph shows the same trend (increasing connectivity) as in the simulations. In the case of panel (d) we can see that the method had some difficulty in estimating several of the changes in *M*_*i*_ values. This is not surprising as some of the randomly simulated changes do not seem to lead to major changes in the IICR curves. This generates again some variance in the *N* and *n* estimates. We also observe a significant variance in the connectivity graph even if several runs overlap nearly perfectly with the simulated connectivity graph.

Altogether the validation tests and figures above suggest that our framework is able to infer changes in connectivity under the *n*-island model, and that some scenarios can be extremely well inferred whereas others may be more difficult depending on their effect on the IICR plots. We also observe that for real data it may be helpful to run the analyses for a varying number of rounds, since too few rounds may negatively affect the quality of the fit. Also, once a scenario has been inferred, it is advisable, as an additional validation step, to simulate data under the inferred scenario and use SNIF to re-infer the parameters. This is what we do with the real human data in the next section.

### Application to humans

Figures 5 and 6 show the results of using SNIF on the human data.

In Fig. 5, panel (a) shows the PSMC curve of the French individual (scaled with a mutation rate of *μ* = 1.25 × 10^−8^ and a generation time of 25 years) together with the best fitting IICR for the model with *c* = 5 and *ω* = 0.2. Panel (b) shows the connectivity graphs of the same inferred demographic scenario. We note that the connectivity pattern consisting of a period of relatively high connectivity between roughly 500 kya and 2 Mya agrees with previous results published in Rodríguez et al. (2018). Note that this study used a mutation rate of *μ* = 2.5 × 10^−8^ and not 1.25 × 10^−8^ as we do here and as originally stated. The absolute timing of events and deme sizes are thus different (see corrections in Rodríguez et al., 2021).

The full set of results related to the inference of human demographies can be found in Figs. S40–S46, which were placed in the Supplementary Materials for the sake of brevity. The most striking feature of this extended set of plots is the sensitivity of the fit to the value of the weight-shifting parameter *ω*. Smaller values allow the optimizer to distribute more of the demographic events towards the ancient past and thus allows this region to be better fitted by the inferred IICR. This functionality (together with being able to ignore certain parts of the plots for the computation of the distance function) can be used to make explicit the knowledge (or beliefs) of the researcher regarding the accuracy of the PSMC curve. We notice that the Yoruba individual cannot be well fitted in the recent past for any value of *ω*, even outside of the designated period of recent population expansion.

Figure 6 shows in panel (a) the number of demes *n* and in panel (b) the reference size *N* that were inferred from each of the five fitted human PSMCs. Of note here is that all individuals except the Yoruba show a consistent value for these inferred parameters across both number of components and value of *ω*. The larger variance of the estimated values for the Yoruba individual suggests that a symmetrical island model may not be enough to explain the patterns of diversity in all five sampled human IICRs.

Figures 7 and 8, and S38 show the results of applying our method to the IICR curves associated with the demographic model for human expansion published by Noskova et al. (2019), which we will refer to as the Classical 3-Populations model—C3PO for short. The C3PO model is a tree-like model with three modern populations that exchange gene flow asymmetrically. It is based on the model of Gutenkunst et al. (2009) and has the same structure but with a higher likelihood and thus can be seen as an improved model with a better fit to the data. The model stipulates the existence of an ancestral population that experienced an increase in size around 275 thousand years ago (Kya), and then a splitting event at about 150 Kya. This split resulted in two populations that exchanged gene flow asymmetrically: a large one that eventually became the modern African population, and a smaller one ancestral to the modern Eurasian population, whatever this terminology may mean. This ancestral lineage split about 22 Kya into the precursors of the European and Asian populations, which at this point began an exponential increase in size that continued to present day. During this period, all three lineages continued to exchange gene flow asymmetrically. The times for these resize and splitting events are represented as dotted vertical gray lines in Fig. 7.

It is clear that the nature of this model does not lend itself to be exactly modeled by a symmetrical *n*-island model, but the piecewise stationarity of our family of models should still be able to pick up the main demographic events. For example, from an *n*-island perspective, a merger or joining of two populations (going backwards in time) may be represented by an increase in gene flow, although this effect may be confounded by the actual changes in both the sizes of the populations and migration rates taking place during these events. Also of note is the fact that the first merger event is not visible to our method because it marks the start of the recent population expansion and is thus excluded from the distance computation.

As can be seen in panel (a) of Fig. 7, these IICRs do not exhibit any major features past the 300 Kya mark, so they do not agree with the human PSMCs of Fig. S37 (of which the representative ones are again shown in Fig. 7 in dashed trace for reference), and they also do not generate significant events in the inferred demographic histories. Particularly, varying the value of the weight-shifting parameter *ω* did not make a great effect in this set of inferences (which is in contrast with the results shown in Fig. 5). This inferred demographic history can be roughly summarized from panel (b) as having a period of relative high gene flow followed by a sharp decrease near the 300 Kya mark, which can be very clearly attributed to the size increase of the ancestral population in the C3PO model.

The inferred number of demes and their relatives sizes for each population can be observed in Fig. 8. The numbers for the African population is in sharp contrast with the other two populations. We can also observe that for the three populations there is more variance (compared to the results from Fig. 6) in the inferred values of *n* and *N* across the different values of *c* and *ω*. This may indicate a weaker link to an underlying *n*-island model.

In general, there is little agreement between the demographic histories inferred by our method from the PSMC data and the simulated IICRs from the C3PO model. This is expected because of how the two models have fundamentally incompatible structures, not only regarding the island versus tree aspect, but also due to the size changes in the C3PO model that affect the IICR potentially as much as gene flow does. However, we do identify the approximate timings of the two visible demographic events when using *c* = 5 components and the more recent-weighted value of *ω* = 1. These results also serve as additional validation that our method will not return the same parameter values regardless of the source of the data. They also suggest that the C3PO model is unlikely to be a good model to understand questions about ancient human structure and evolution.

## Discussion

Our validations show that the inference framework presented here is able to accurately infer structure parameters (number of islands and their sizes) within a symmetrical island model given an IICR estimate like the PSMC. It is also able to date up to five events of changes in migration rate (i.e., six components) with good precision and consistency, as long as the underlying model is compatible with a symmetrical island model. The nRMSD (Fig. S20) of the simulated vs. inferred scenario parameters is zero for stationary scenarios (*c* = 1), and increases linearly with the number of components. For the *M* parameters it reaches a value of about 0.5 at six components, and we see that the first and last components are better estimated than the middle ones. It is likely that the component misidentification phenomenon is contributing to this effect. The number of islands *n* and the reference effective size *N* are consistently well estimated, reaching an nRMSD of about 0.1 in the worst cases. The *t*_*i*_ parameters exhibit the worst nRMSD values, varying between 1 and 2 in the worst cases. Although in these cases, the fact that time is log-spaced and spans several orders of magnitude causes outliers to have a disproportionate contribution to this statistic.

### Human evolution

An application of our method to five publicly available human PSMCs suggests that the backwards long term history of the sampled individuals, when accounting for possible recent expansions and the noise introduced by the PSMC method, can be accurately modeled in the framework of a symmetrical island model of ~10–12 demes with varying levels of connectivity through time. Only one of the five samples (Yoruba) displayed less consistent evidence of this finding, which may indicate that more complex models (possibly including asymmetric gene flow, spatial modeling of the environment, or changes in deme sizes) could be needed to explain the full complexity of the data.

These findings regarding changes in connectivity and number of islands are in agreement with the results of Rodríguez et al. (2018), in which a hand-fitting approach of the IICRs was used to arrive at an estimate of 10 islands with a similar value of *N* and a comparable period featuring a significant increased of gene flow between 600 Kya and 2 Mya. Note that the timing in years and the deme sizes in Rodríguez et al. (2018) differ due to the change in mutation rate (Rodríguez et al., 2021).

We also compared our results with the tree model for human evolution published by Noskova et al. (2019) (the C3PO model), which is a revision of the model from Gutenkunst et al. (2009) and represents a simplified model of human evolution (Jouganous et al., 2017, Kamm et al., 2019). The C3PO model proposes an ancestral human population that experiences two splits: an old one that resulted in the current African “population” and another more recent one that resulted in the current European and Asian “populations”. The parameters of this model include the times of these events, the population size history of these populations and their ancestral branches, and the migration rates between them. The summary statistic targeted by these methods is the AFS, and we see that a fitting AFS does not guarantee a fitting IICR and vice versa (Beichman et al., 2017, Chikhi et al., 2018). Indeed, the IICRs of the populations from the C3PO model do not resemble those of the real humans. Likewise, when we use the C3PO model to generate IICR curves, and infer the corresponding demographic history using SNIF, we find results that do not resemble those obtained from the human IICRs, and are less consistent across different runs than when inferring directly from the human IICRs.

These findings suggest that tree models fitted with the AFS like those considered here do not offer a definitive answer to the question of human evolution and other families of models should be explored (Goldstein and Chikhi, 2002, Scerri et al., 2019, 2018). It remains to be seen however how well models inferred with our method fit the real AFS of their respective human populations. A general treatment of this question is beyond the work presented here. However, in section S5.2 of the Supplementary Materials we compare the AFS of a sample of 216 humans from the Yoruba population (Lapierre et al., 2017) to the one inferred by the GADMA method from Noskova et al. (2019) and the one corresponding to three variations of the inferred demographic model by our method (see Fig. S47). These simulations suggest that existing AFSs could be easily fitted with a structured model similar to those inferred by SNIF, but in which we would allow for a recent population size change.

### Future work

One novel aspect of our approach is that the number of demes gets inferred as one of the model parameters, and it is in fact the best estimated parameter, which is in agreement with Mazet et al. (2015) that used information from the distribution of *T*_2_ values and a likelihood approach. These authors however, only analysed stationary models. Here we found that other parameters were also well estimated when the number of components was low, but we also observed that the estimated value of *n* scaled well with increasing model complexity. A similar consistency can be observed with the deme size parameter *N* (see Fig. S20). We give up some flexibility in the model by keeping the number of demes constant throughout the history of the population, so the timed demographic events cannot represent splits or joining of populations even though such events are likely to have taken place in the history of species. Additionally, in the *n*-island model we do not account for possible asymmetrical gene flow or different deme sizes, even when the theoretical framework does allow for such representations. However, it is a more challenging problem to validate due to the fact that during any given component, changing both the migration rate and the deme size have confounding effects on the IICR curve which can be hard to separate. This requires a dedicated study with a different methodology which we will explore in a future work.

Another potential direction is to use multiple IICR curves simultaneously during the inference process. These multiple IICRs may come in the form of more than one IICR sampled from an asymmetrical demographic model (for which the initial sampling deme *does* result in different curves (Chikhi et al., 2018), as opposed to the *n*-island model where demes are by definition indistinguishable). They may also be in the form of multiple IICR_*k*_ curves where *k* is the number of sampled haploid genomes. Indeed, the IICR of Mazet et al. (2016) was defined for *k* = 2, and this is the IICR that we have been studying in our previous works. However, the concept can be extended to more haploid genomes in the same way that the MSMC method (Schiffels and Durbin, 2013) is an extension of the PSMC to multiple genomes, which takes into consideration the distribution of the coalescent time *T*_*k*_. The precise concept of the IICR_*k*_ is currently being developed in a separate study. These approaches may prove beneficial in choosing between structured and non-structured models. Indeed, Grusea et al. (2018) shows that using more than one IICR curve can help discriminate between structured and nonstructured scenarios in the *n*-island model. Finally, the incorporation of larger samples not only enables exploring more complex scenarios, but it also allows using other summary statistics to complement the IICR, most notably among them the AFS, which is widely used for the purposes of demographic inference.

### Conclusion

In summary, we have presented here an inference method for automatically estimating demographic parameters under a piecewise stationary symmetrical island model that uses the IICR as its summary statistic. The underlying methodology consists in quantifying the discrepancy between a target IICR and many simulated IICR curves for a large number of candidate scenarios, and using this metric to drive a global optimization process. With a large number of validations we have shown that the method works accurately and consistently for a diverse range of parameter values, and we additionally showed an application to human data that agrees with and improves upon previously published results using similar approaches.

We believe that despite its current scope, our method can be of great value during the initial exploration of the parameter space for simple models, and thus can also provide a starting point for manually fitting the IICR with models that could express spatial structure and varying *N*(Rodríguez et al., 2018).

## Supplementary information

Article supplementary materials

## Data Availability

The implementation of SNIF its documentation, and the data and scripts required to reproduce our results can be found in github.com/arredondos/snif.
