# Correction: Inferring number of populations and changes in connectivity under the n-island model

**DOI:** 10.1038/s41437-022-00511-7

**Published:** 2022-03-17

**Authors:** Armando Arredondo, Beatriz Mourato, Khoa Nguyen, Simon Boitard, Willy Rodríguez, Olivier Mazet, Lounès Chikhi

**Affiliations:** 1 Université de Toulouse, Institut National des Sciences Appliquées, Institut de Mathématiques de Toulouse, Toulouse, France; 2 Institut de Mathématiques de Toulouse; UMR5219. Université de Toulouse, Toulouse, France; 3 Instituto Gulbenkian de Ciência, Oeiras, Portugal; 4 CBGP, Université de Montpellier, CIRAD, INRAE, Institut Agro, IRD, Montpellier, France; 5 ENAC - Ecole Nationale de l’Aviation Civile, Université de Toulouse, Toulouse, France; 6 Laboratoire Évolution & Diversité Biologique (EDB UMR 5174), CNRS, IRD, UPS, Université de Toulouse Midi-Pyrénées, Toulouse, France

**Keywords:** Population genetics, Biological models, Population genetics

Correction to: *Heredity* 10.1038/s41437-021-00426-9, published online 12 April 2021

The author originally listed as Camille Noûs on this article [1] is fictitious (http://www.cogitamus.fr/indexen.html) and as such does not fulfil the requirements for authorship. The correct authorship list is: Armando Arredondo, Beatriz Mourato, Khoa Nguyen, Simon Boitard, Willy Rodríguez, Olivier Mazet & Lounès Chikhi. This has been corrected.

The authors have provided the following addition to their Acknowledgements statement:

“The original version of this article [1] included Camille Noûs as a co-author. Camille Noûs is not a real person but a symbolic author who embodies the collegial nature of our work (https://www.cogitamus.fr/camilleen.html). As many other scientists before us, we wished to include Camille Noûs as a co-author to acknowledge the contribution of our academic community and emphasize that the construction and dissemination of scientific knowledge should be intrinsically selfless, collaborative and open. Springer Nature expressed disagreement with this co-authorship, arguing that Camille Noûs does not comply to the authorship rules set forth by ICMJE. These rules were created because some humans behave in non-ethical ways and authorship rules thus provide important safeguards against such behaviors, but we believe that they should only apply to humans. We also believe in the importance of publishing in historical journals edited in part at least by a scientific society, like Heredity. For these reasons we regret this publisher’s decision. Despite the disagreement, we thank Springer Nature for allowing us to write this text, which we hope will contribute to open debates on (and improve) a publication and evaluation system that needs change.”
