# Supplementary material for: Inferring number of populations and changes in connectivity under the n-island model
Source: Heredity (Edinb). 2021 Apr 12;126(6):896–912. doi: 10.1038/s41437-021-00426-9 (PMC8178352; doi:10.1038/s41437-021-00426-9)
Supplement: Supplementary file 1 — Article supplementary materials [file 41437_2021_426_MOESM1_ESM.pdf]

Supplementary Materials for: Inferring number of populations and changes in connectivity under the n-island model

Armando Arredondo, Beatriz Mourato, Khoa Nguyen,
Simon Boitard, Willy Rodríguez, Camille Noûs,
Olivier Mazet, Lounès Chikhi

Contents

S1 Preliminary results on the IICR **2**
S1.1 Computing the IICR 2
S1.2 Scaling the IICR 4
S1.3 Proof of Lemma 1 5

S2 Additional notes on the optimization framework **6**
S2.1 Parameter bounds 6
S2.2 Types of target IICRs 6
S2.3 Comparison of optimization parameters 7

S3 Results of validation using exact simulated IICRs **9**
S3.1 On the number of inference rounds 9
S3.2 Unscaled IICR 10
S3.3 Scaled IICR 17
S3.4 Quantifying the inference error 23

S4 Results of validation using T-sim IICRs **24**
S4.1 Scenarios with 1 component 24
S4.2 Scenarios with 2 components 27
S4.3 Scenarios with 3 components 30
S4.4 Scenarios with 4 components 33
S4.5 Scenarios with 5 components 36

S5 Results of application to human data **39**
S5.1 Seq-Sim validations 39
S5.2 AFS comparisons 49

S6 A note on implementation and use cases **50**

References **51**

S1. Preliminary results on the IICR

Interpreting the IICR as a change in population size could be misleading in the presence of demographic structure. In figure S1, we present an example from Mazet et al. (2016) where a spurious bottleneck effect due to population structure is so strong that it hides the increase in size of all demes in the recent past. This example also stresses how structure can profoundly impact genomic patterns of diversity.

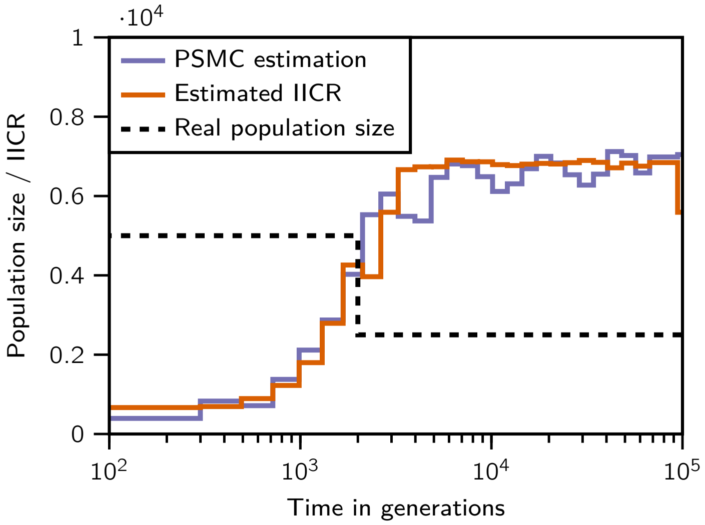

Figure S1: A reading of this IICR curve as a size change function would indicate that the population has decreased in size in the recent past, where in fact the opposite is true and the population has experienced a doubling of size in the recent past.

S1.1. Computing the IICR

Considering the c components which constitute the model, we note that during component i, taking $0 \leqslant i \leqslant \gamma = c - 1$, the underlying coalescent process X_t is being governed by an n-island model which has transition rate (see Rodríguez et al. (2018) for details and a more general approach):

$$Q_i = \begin{pmatrix} -M_i - \frac{1}{s_i} & M_i & \frac{1}{s_i} \\ \frac{M_i}{n-1} & -\frac{M_i}{n-1} & 0 \\ 0 & 0 & 0 \end{pmatrix},$$

where the three states in the matrix Q_i are sorted, both by row and column, as:

1. the two sampled lineages are in the same deme (configuration 'same');
2. the two sampled lineages are in different demes (configuration 'diff.');
3. the sampled lineages have coalesced (absorption).

Additionally, the rates in this matrix are active during the time interval $[t_i; t_{i+1})$, where we understand t_0 and $t_{\gamma+1}$ to be 0 and $+\infty$ respectively.

We know from that the probability distribution of T_2 is given by the cumulative effects of the exponential functions e^{tQ_i} (see Hobolth et al. (2019) for an extensive review). More formally, given any $t > 0$, let i be the largest index such that $t_i \leqslant t$, for which case we have:

$$P(t) = \left(\prod_{k=1}^{i} e^{(t_k - t_{k-1})Q_{k-1}}\right) e^{(t-t_i)Q_i},$$

$$D(t) = \frac{dP(t)}{dt} = \left(\prod_{k=1}^{i} e^{(t_k - t_{k-1})Q_{k-1}}\right) Q_i \, e^{(t-t_i)Q_i}. \quad (1)$$

Since component (p, q) in matrix $P(t)$ gives the probability $\mathbb{P}(X_t = q \mid X_0 = p)$, then the random variable $T_{2,\text{same}}$ of the time until coalescence of two lineages sampled in the *same* deme (initial state 1) has distribution $F_{\text{same}}(t) = P(t)_{(1,3)}$ and density $f_{\text{same}}(t) = D(t)_{(1,3)}$, and for the case where we sample in two *different* demes (initial state 2), then $T_{2,\text{diff.}}$ would have its distribution given by $F_{\text{diff.}}(t) = P(t)_{(2,3)}$ and its density by $f_{\text{diff.}}(t) = D(t)_{(2,3)}$.

The factor matrices in (1) can be computed in several ways in the general case (see Herbots (1994) or Hobolth et al. (2019)), but considering this particular instance of size 3×3, they

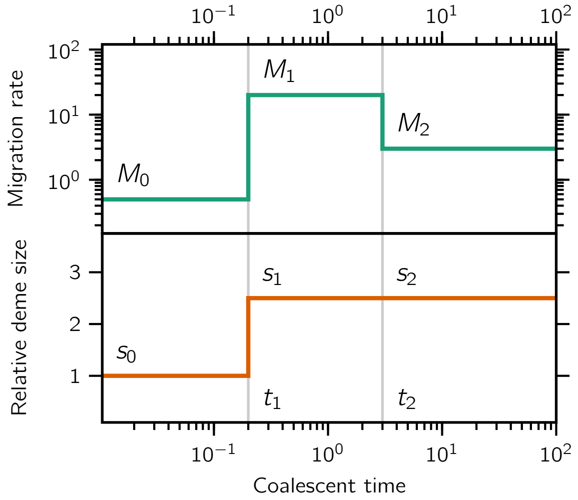

Figure S2: Connectivity and population size graphs. Visual representation of the t_i, M_i and s_i of a demographic model with $c = 3$ components. We refer to the upper part of the figure as a connectivity graph. The lower part represents population size changes of the general model. In the present study the s_i are identical and equal to 1 and will not be represented with connectivity graphs. The number of islands is inferred but constant, and it is not shown in this figure (although it is usually shown in a separate figure or panel).

may also be computed directly given an arbitrary Δt and rate matrix Q:

$$e^{\Delta t\ Q} = \begin{pmatrix} F_{11} & F_{12} & 1 - F_{11} - F_{12} \\ F_{21} & F_{22} & 1 - F_{21} - F_{22} \\ 0 & 0 & 1 \end{pmatrix}, \tag{2}$$

where:

$$\begin{aligned} F_{11} &= \frac{(\delta + \alpha - 2Ms)exp_2 + (\delta - \alpha + 2Ms)exp_1}{2\delta}, & exp_1 &= \exp\Big(\frac{\Delta t(\delta - \alpha)}{2s(n-1)}\Big), \\ F_{12} &= (n-1)F_{21}, & exp_2 &= \exp\Big(\frac{\Delta t(-\delta - \alpha)}{2s(n-1)}\Big), \\ F_{21} &= \frac{Ms(exp_1 - exp_2)}{\delta}, & \delta &= \sqrt{\alpha^2 - 4Ms(n-1)}, \\ F_{22} &= \frac{(\delta + \alpha - 2Ms)exp_1 + (\delta - \alpha + 2Ms)exp_2}{2\delta}, & \alpha &= Mns + n - 1. \end{aligned} \tag{3}$$

With this we can efficiently compute either IICR functions:

$$\text{IICR}_{\text{same}}(t) = \frac{1 - F_{\text{same}}(t)}{f_{\text{same}}(t)}, \qquad \text{IICR}_{\text{diff.}}(t) = \frac{1 - F_{\text{diff.}}(t)}{f_{\text{diff.}}(t)}. \tag{4}$$

S1.2. Scaling the IICR

In contrast with the parameter space for the unscaled IICR ($\Phi_{\gamma,B}$) in which there is a one-to-one correspondence between a parameter tuple and the corresponding IICR curve, there are only $3\gamma + 3$ independent degrees of freedom in $\hat{\Phi}_{\gamma,B}$, even though there are $3\gamma + 4$ parameters. This notion is formalized by the following lemma.

Lemma 1. *Given any $\hat{\varphi} = (N, n, t_1 \dots t_\gamma, M_0 \dots M_\gamma, s_0 \dots s_\gamma) \in \hat{\Phi}_{\gamma,B}$, then the parameter tuple:*

$$\hat{\varphi}_0 = \Big(\frac{N}{C}, n, Ct_1 \dots Ct_\gamma, \frac{M_0}{C} \cdots \frac{M_\gamma}{C}, Cs_0 \dots Cs_\gamma\Big)$$

is such that:

$$\text{sIICR}_{\hat{\varphi}}(g) = \text{sIICR}_{\hat{\varphi}_0}(g) \qquad \forall g > 0,$$

where C is any rescaling factor for which the coordinates of $\hat{\varphi}_0$ are within the bounds B.

The implication of Lemma 1 is that when trying to infer all the parameters of $\hat{\varphi}$ simultaneously, the only parameter for which we may get an absolute estimate is n, as the rest of them can only be distinguished up to an unknown re-scaling factor C. Note that this un-identifiability issue is different from the one identified in Mazet et al. (2016) regarding the inability to discriminate between panmictic and non-panmictic demographies with a single IICR. However, we stress here that in practice this is not necessarily an issue because it suffices to fix one of the model parameters (for instance, $s_0 = 1$) to be able to uniquely map any sIICR curve to its parameters. In the case of constant size this is even less of an issue, since *all* deme sizes are fixed to $s_i = 1$ and thus no further considerations are necessary.

S1.3. Proof of Lemma 1

Lemma 2. *Given any* $\hat{\varphi} = (N, n, t_1 \dots t_\gamma, M_0 \dots M_\gamma, s_0 \dots s_\gamma) \in \hat{\Phi}_{\gamma,B}$*, then the parameter tuple:*

$$\hat{\varphi}_0 = \Big(\frac{N}{C}, n, Ct_1 \dots Ct_\gamma, \frac{M_0}{C} \cdots \frac{M_\gamma}{C}, Cs_0 \dots Cs_\gamma\Big)$$

is such that:

$$\text{sIICR}_{\hat{\varphi}}(g) = \text{sIICR}_{\hat{\varphi}_0}(g) \qquad \forall g > 0,$$

where C *is any rescaling factor for which the coordinates of* $\hat{\varphi}_0$ *are within the bounds* B*.*

Proof. Let us denote by $\pi_{n,M,s}(t)$ the factors e^{tQ} that appear in the definition of $P(t)$ (equations 1 and 2). It is easy to verify that for any $C > 0$, we have:

$$\pi_{n,M,s}(t) = \pi_{n,\frac{M}{C},Cs}(Ct). \tag{5}$$

Indeed, from the expressions in (3) we can see that the parameter M always appears in the factor Ms, and the parameter t always appears in the factor t/s, which are invariant under the transformation $(M, s, t) \mapsto (\frac{M}{C}, Cs, Ct)$.

Next, given any:

$$\varphi = (n, t_1 \dots t_\gamma, M_0 \dots M_\gamma, s_0 \dots s_\gamma),$$
$$\varphi_0 = (n, Ct_1 \dots Ct_\gamma, \frac{M_0}{C} \cdots \frac{M_\gamma}{C}, Cs_0 \dots Cs_\gamma),$$

and any $t > 0$, we can write $P(t)$ from (1) as:

$$\begin{aligned} P_\varphi(t) &= \Big(\prod_{k=1}^{i} \pi_{n,M_{k-1},s_{k-1}}(t_k - t_{k-1})\Big)\pi_{n,M_i,s_i}(t - t_i) \\ &= \Big(\prod_{k=1}^{i} \pi_{n,\frac{M_{k-1}}{C},Cs_{k-1}}(Ct_k - Ct_{k-1})\Big)\pi_{n,\frac{M_i}{C},Cs_i}(Ct - Ct_i) \\ &= P_{\varphi_0}(Ct). \end{aligned} \tag{6}$$

where i is the largest index such that $t_i \leqslant t$ and subsequently $Ct_i \leqslant Ct$. We denote now by $F_\varphi(t)$ any of $F_{\text{same}}(t) = P_\varphi(t)_{(1,3)}$ or $F_{\text{diff.}}(t) = P_\varphi(t)_{(1,2)}$. From (6) we have $F_\varphi(t) = F_{\varphi_0}(Ct)$. In order to introduce scaling, we consider an arbitrary effective size N and perform the corresponding change of variable $t = g/2N$. Thus, by having $\hat{\varphi} = (N, \varphi)$ and $\hat{\varphi}_0 = (N/C, \varphi_0)$, we get:

$$\begin{aligned} & F_\varphi\Big(\frac{g}{2N}\Big) = F_{\varphi_0}\Big(\frac{Cg}{2N}\Big) \\ \Rightarrow\quad & \frac{1}{2N} f_\varphi\Big(\frac{g}{2N}\Big) = \frac{C}{2N} f_{\varphi_0}\Big(\frac{Cg}{2N}\Big) \\ \Rightarrow\quad & N\,\frac{1 - F_\varphi(g/2N)}{f_\varphi(g/2N)} = \frac{N}{C}\,\frac{1 - F_{\varphi_0}(Cg/2N)}{f_{\varphi_0}(Cg/2N)} \\ \Rightarrow\quad & \text{sIICR}_{\hat{\varphi}}(g) = \text{sIICR}_{\hat{\varphi}_0}(g) \end{aligned}$$

■

S2. Additional notes on the optimization framework

S2.1. Parameter bounds

During the continuous-sampling validation phase (section Sampling the parameter space of the main), we generated 400 scenarios by randomly sampling their parameter values. The continuous distribution for each parameter was bounded by the values under the *simulation* columns in Table S1. When inferring the parameters with SNIF, we supplied wider ranges for all the parameters (*inference* columns).

	**simulation**		**inference**	
	min	max	min	max
n	2	40	2	50
t_i	0.1	50	0.01	100
M_i	0.1	50	0.05	60
s_i	1	1	1	1
N	10^3	10^3	10^2	10^4

Table S1: Parameter bounds used during the generation of demographic scenarios for validation.

S2.2. Types of target IICRs

In this paper we used three different methods when generating simulated IICR curves for validation (see section The three types of target IICRs in the main text). Figure S3 summarizes the differences between them.

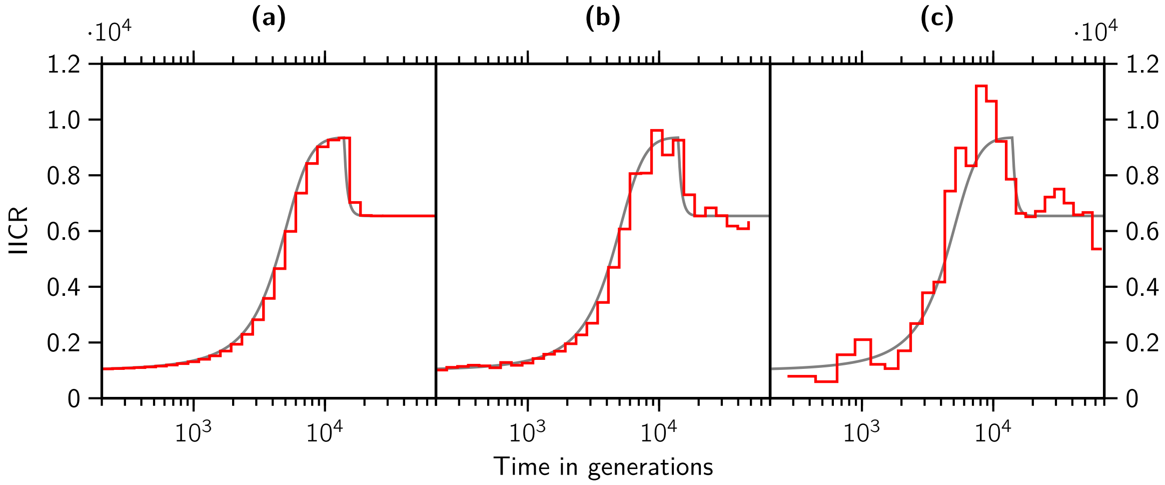

Figure S3: The three ways of obtaining a target IICR. The panels represent different types of discretised target IICRs for the same two-components demographic history with $N = 10^3$, $n = 4$, $t_1 = 7$, $(M_i) = (0.5, 1.0)$ and $(s_i) = (1.0, 1.0)$. **(a)** Exact IICR, computed as per section S1.1 and discretized to 32 intervals. In gray we show a smoother discretization and keep it in the other panels for reference. This is obtained using the approach of Rodríguez et al. (2018). **(b)** T-sim IICR, obtained by simulating 2×10^4 T_2 samples using the `ms` command `ms 2 20000 -T -L -I 4 2 0 0 0 0.5 -eM 3.5 1 -eN 3.5 1` and later scaling the IICR using the value of N. This is the approach used in Chikhi et al. (2018) **(c)** Seq-sim IICR, obtained by running PSMC on a genome simulated using the `ms` command `ms 2 100 -t 100 -r 20 2000000 -p 8 -I 4 2 0 0 0 0.5 -eM 3.5 1 -eN 3.5 1` and later scaling with mutation rate $\mu = 1.25 \times 10^{-8}$. This is obtained by running the PSMC method of Li and Durbin (2011)

S2.3. Comparison of optimization parameters

Here we explore the effect of various parameters of the optimization algorithm on the speed of convergence of the inference process.

The most important parameters that affect the search process and convergence criteria are:

- `strategy`: it can be one of 12 possible values (see Table 1) and controls how each new generation of solutions are computed from the previous one. Default is '`best1bin`'.
- `maxiter`: maximum number of iterations the algorithm will perform before forcing convergence. Default is 5000.
- `popsize`: number of simultaneous solutions during any given generation. Default is 15.
- `tol`: relative tolerance for search convergence. The convergence criteria is met when the standard deviation of the solutions within a generation is smaller than `tol` times the average energy (in our case, distance) within that generation. Default is 10^{-2}.
- `mutation`: per-generation mutation rate of the solutions. The default behaviour is to draw a random value from a uniform distribution in $[0.5, 1]$ each generation.
- `recombination`: per-generation recombination rate of the solutions. Default is 0.7.

We selected 10 random simulated scenarios with unscaled IICR and $c = 4$ components from the set of exact IICR validations that had not converged before 500 rounds (these would correspond to scenarios off the diagonals in Figure S9). For each one of them, and for each of the 12 possible values for the `strategy` parameter, we attempted another 100 rounds. Table 1 shows the number of rounds it took for each of the 10 scenarios to converge in each case (a value of 100 means that convergence was not reached).

Strategy	#1	#2	#3	#4	#5	#6	#7	#8	#9	#10	Total
best2exp	17	1	100	2	100	22	3	100	57	22	424
best2bin	6	35	100	2	11	93	100	4	33	100	484
currenttobest1exp	2	80	100	5	100	69	12	100	8	26	502
best1exp	3	76	100	21	4	100	10	100	62	100	576
rand1exp	4	100	100	10	100	14	100	100	100	38	666
randtobest1exp	32	100	100	9	100	78	100	30	100	100	749
currenttobest1bin	21	100	100	27	100	100	100	94	63	88	793
best1bin	27	100	100	28	54	100	100	100	100	100	809
rand2exp	40	100	100	100	100	24	100	100	100	100	864
rand1bin	100	100	100	3	100	100	100	100	100	100	903
rand2bin	96	100	100	23	100	100	100	100	100	100	919
randtobest1bin	92	100	100	100	100	100	100	100	100	100	992

Table S2: Results of varying the `strategy` parameter of the differential evolution algorithm on the speed of convergence of 10 difficult demographic scenarios with $c = 4$ components.

As can be seen, strategy '`best2exp`' was best with a combined number of 424 rounds for the 10 scenarios.

Next, using this optimal strategy parameter, we tried one alternative value for the rest of the optimization parameters at a time, again allowing a maximum of 100 rounds. The alternative values were as follows:

- `maxiter` was changed from 5000 to 10000.
- `popsize` was changed from 15 to 50.
- `mutation` was changed from random sample in $[0.5, 1]$ to random sample in $[0.5, 1.7]$.
- `recombination` was changed from 0.7 to 0.9.

The results, shown in Table 2, suggest that these parameters should be left at their default values.

Parameter	no. 1	no. 2	no. 3	no. 4	no. 5	no. 6	no. 7	no. 8	no. 9	no. 10	Total
popsize	100	100	100	100	100	100	100	100	100	100	1,000
recombination	100	100	100	100	100	100	100	100	100	100	1,000
mutation	100	100	100	100	100	100	100	100	100	100	1,000
max-iter	100	100	100	100	100	100	100	100	100	100	1,000

Table S3: Results of varying the `popsize`, `recombination`, `mutation` and `max-iter` parameters of the differential evolution algorithm on the speed of convergence of 10 difficult demographic scenarios with $c = 4$ components.

S3. Results of validation using exact simulated IICRs

S3.1. On the number of inference rounds

In figures S4 and S5 we explore the question of how many rounds of inference are needed in order to achieve optimal distance. These results are only of theoretical interest since in real applications, the target IICR is not known exactly (just approximated) therefore the optimal distance of 0 cannot be achieved in any meaningful way.

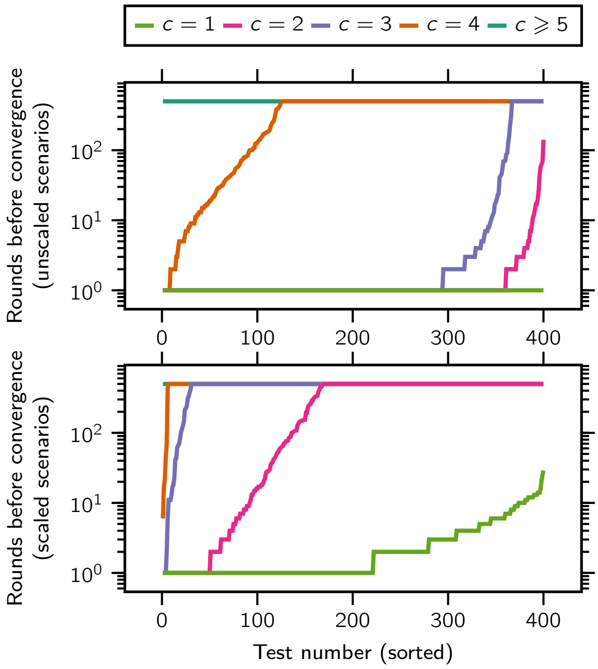

Figure S4: Number of performed rounds during validations with exact IICRs. The top panel shows the number of rounds (up to a maximum of 500) that the method required before converging to a scenario with a distance value smaller than the tolerance $\varepsilon = 10^{-10}$ for the unscaled case. The bottom panel shows the same information for scaled scenarios and a tolerance of $\varepsilon = 10^{-7}$. We represent in different colors the curves corresponding to scenarios with different simulated (and inferred) number of components, ranging from $c = 2$ up to $c = 6$; the same data-set represented in figures S6 to S11 (top panel) and figures S13 to S18 (bottom panel). Higher number of rounds is not correlated with worse fit when the maximum number of rounds is not reached; and when it is reached it is not necessarily an indication of bad fit either, although all instances of bad fit stem from inferences that reached the maximum allowed number of rounds.

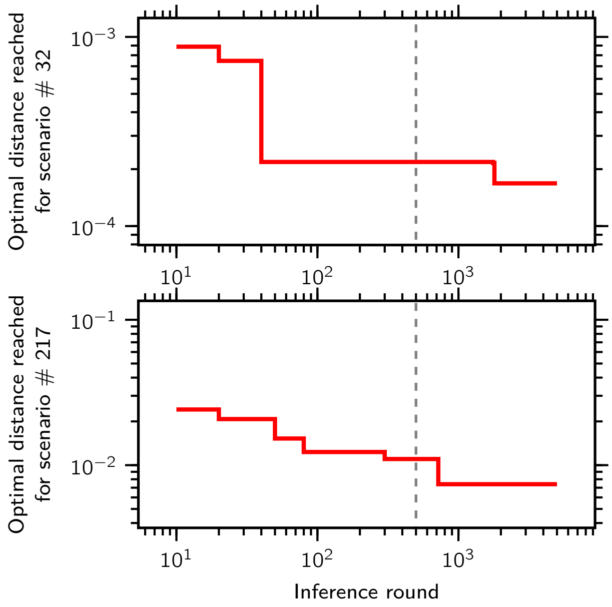

Figure S5: Convergence of the inferred IICR with the progression of the inference rounds. We measured the best distance achieved ($\omega = 1$) as a function of the completed rounds for up to 5000 rounds. The two curves correspond to two $c = 6$ component scenarios that did not previously converge ($\varepsilon = 10^{-10}$) in 500 rounds (marked with a vertical dashed line). We note that there is a clear trend for the distance between the IICR curves to decrease with more inference rounds, but there is also diminishing returns to performing a very large number of rounds, since the issue of component misidentification may be an insurmountable difficulty for some scenarios (see main text for a more detailed discussion).

S3.2. Unscaled IICR

In this section we show in figures S6 to S11 the validation results of simulating and then inferring from 400 randomly generated demographic scenarios with unscaled IICRs and varying number of components c. Each figure consists of as many sub-panels as there are free parameters for that model, and in each one the simulated values are in the horizontal axis and the inferred ones in the vertical axis.

In the lower-right corner of each sub-panel we display the normalized root-mean-square deviation (nRMSD) of the simulated versus inferred vectors. Additionally, we indicate in grey the region of 10% relative error (50% for the t_i parameters). The percentage of tests that fall within this and other margins of error is summarised in Figure S12.

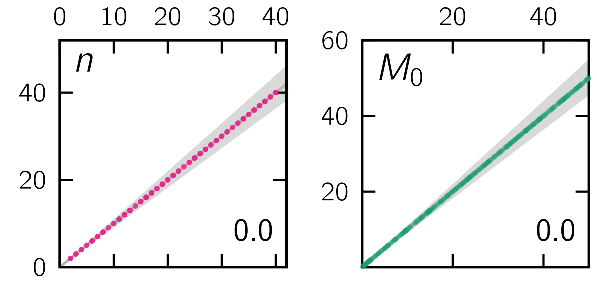

Figure S6: Scatter plots of all the simulated (horizontal axis) versus inferred (vertical axis) parameter values for scenarios of $c = 1$ component.

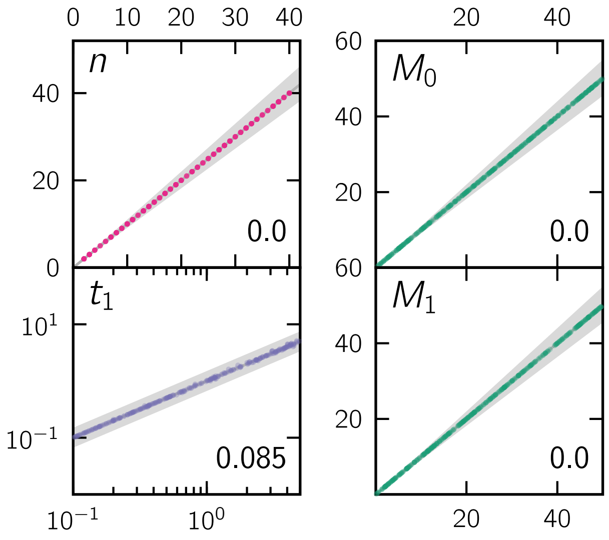

Figure S7: Scatter plots of all the simulated (horizontal axis) versus inferred (vertical axis) parameter values for scenarios of $c = 2$ components.

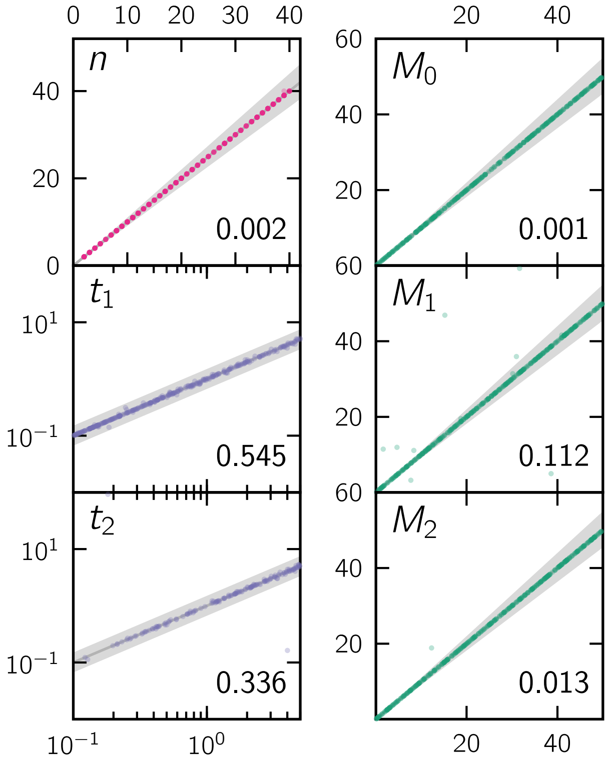

Figure S8: Scatter plots of all the simulated (horizontal axis) versus inferred (vertical axis) parameter values for scenarios of $c = 3$ components.

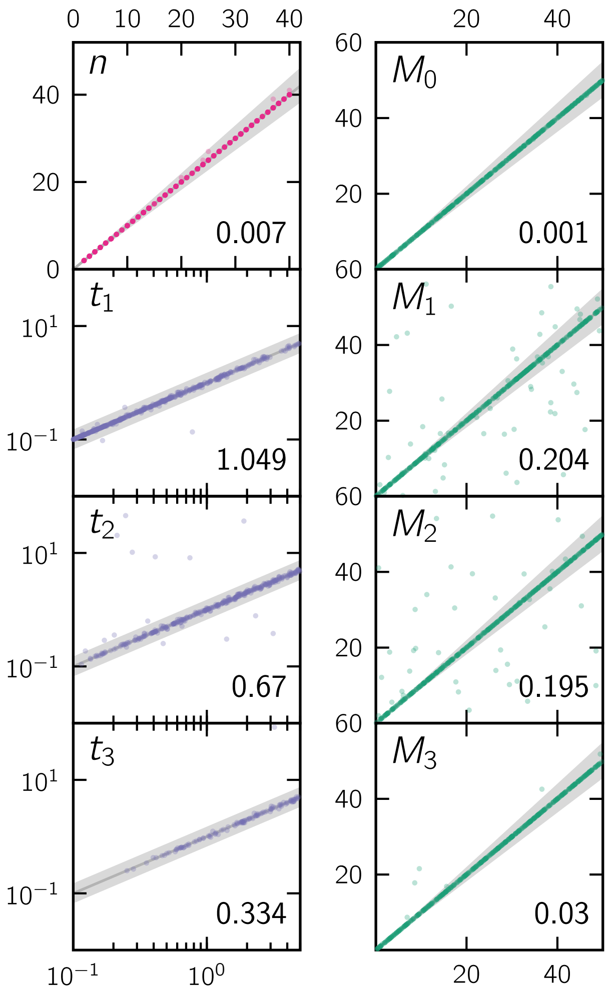

Figure S9: Scatter plots of all the simulated (horizontal axis) versus inferred (vertical axis) parameter values for scenarios of $c = 4$ components.

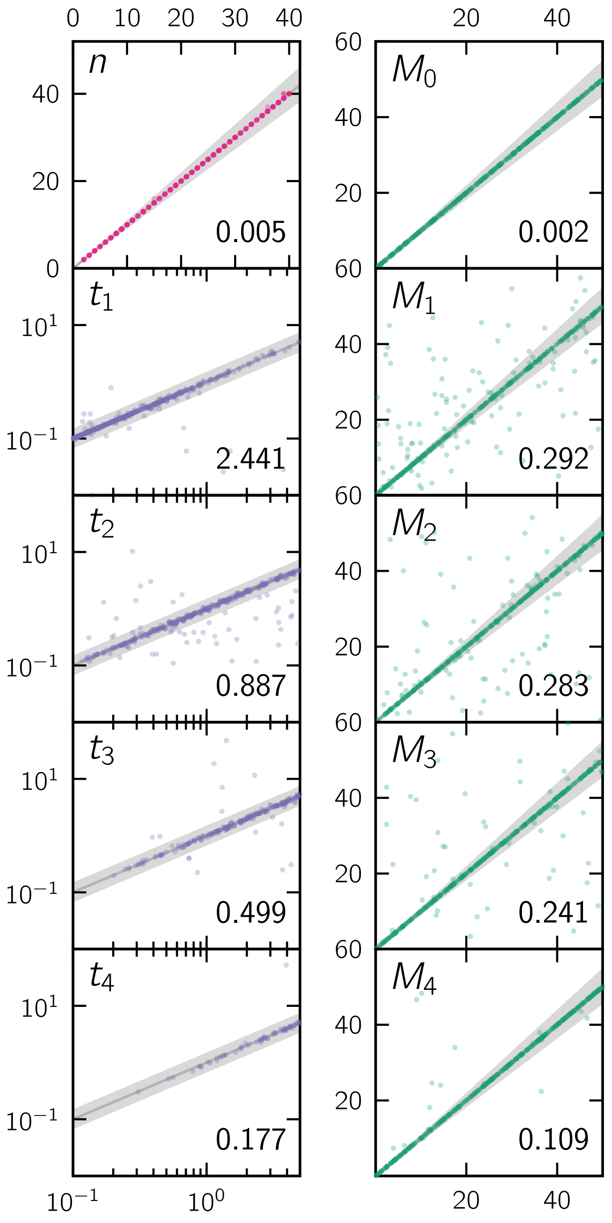

Figure S10: Scatter plots of all the simulated (horizontal axis) versus inferred (vertical axis) parameter values for scenarios of $c = 5$ components.

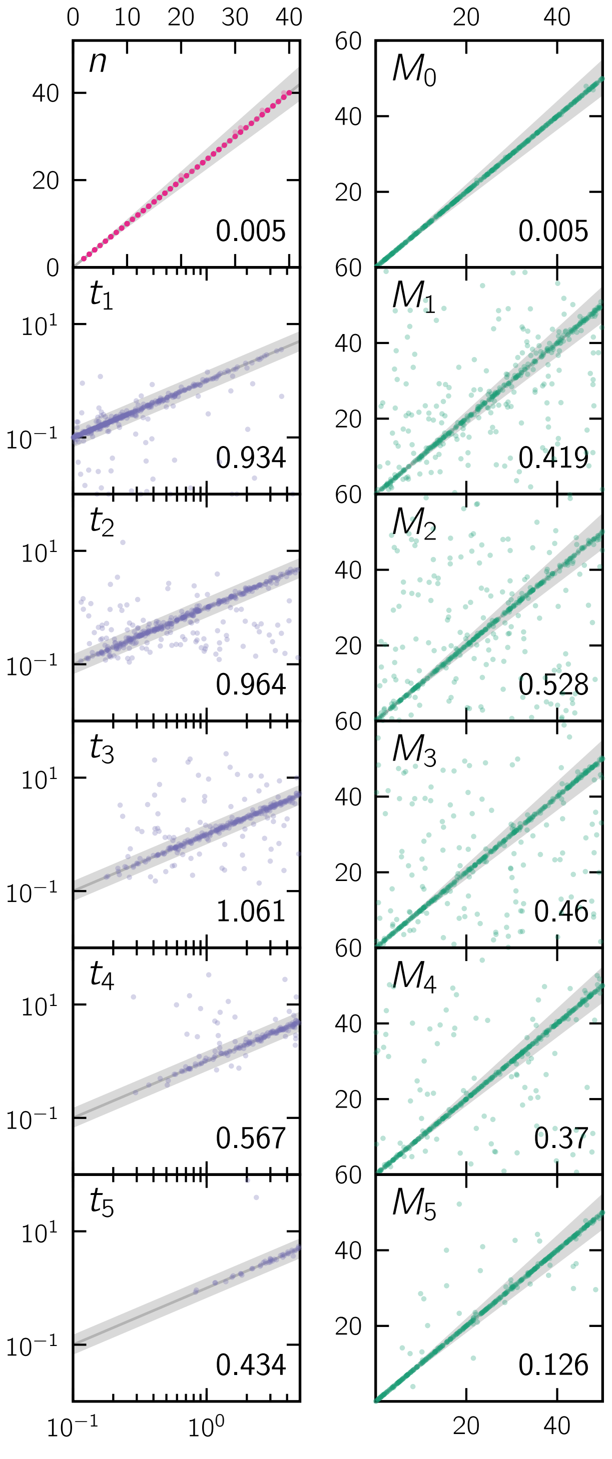

Figure S11: Scatter plots of all the simulated (horizontal axis) versus inferred (vertical axis) parameter values for scenarios of $c = 6$ components.

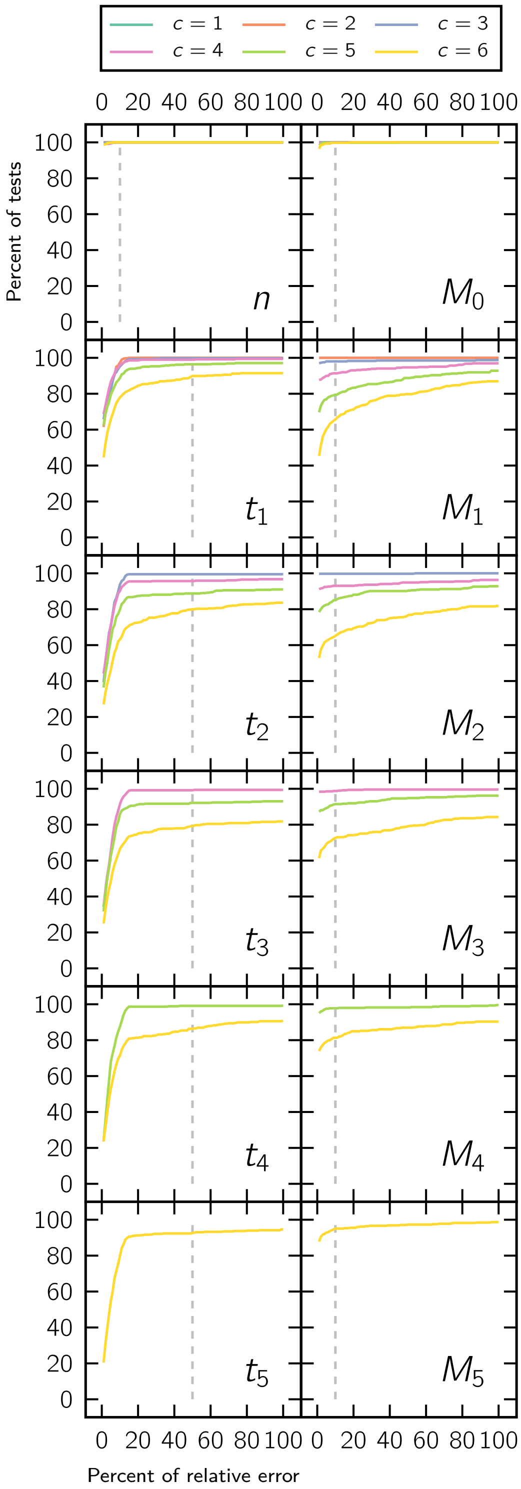

Figure S12: Percent of tests within a given relative error in unscaled scenarios. For each parameter of the demographic scenarios, we counted the instances where the inferred value was within a certain percent of the simulated one. We say that an inferred parameter $\bar{p}$ is within x% of the simulated value p if $-x\hat{p} < 100(\hat{p} - p) < xp$. Note that not all parameters are present in all scenarios (for instance, the parameter M_4 only appears in scenarios with $c \geqslant 5$ components).

S3.3. Scaled IICR

In this section we show in figures S13 to S18 the validation results of simulating and then inferring from 400 randomly generated demographic scenarios with scaled IICRs and varying number of components c. Each figure consists of as many sub-panels as there are free parameters for that model, and in each one the simulated values are in the horizontal axis and the inferred ones in the vertical axis. The deme size panel (N) is different because the simulated values for N was always $N = 1000$, therefore the horizontal axis indicates the test number (1 to 400) and the vertical axis the inferred value.

In the lower-right corner of each sub-panel we display the normalized root-mean-square deviation (nRMSD) of the simulated versus inferred vectors. Additionally, we indicate in grey the region of 10% relative error (50% for the t_i parameters). The percentage of tests that fall within this and other margins of error is summarised in Figure S19.

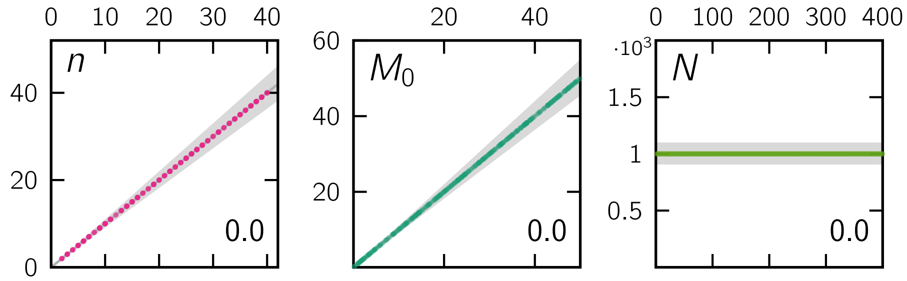

Figure S13: Scatter plots of all the simulated (horizontal axis) versus inferred (vertical axis) parameter values for scenarios of $c = 1$ component.

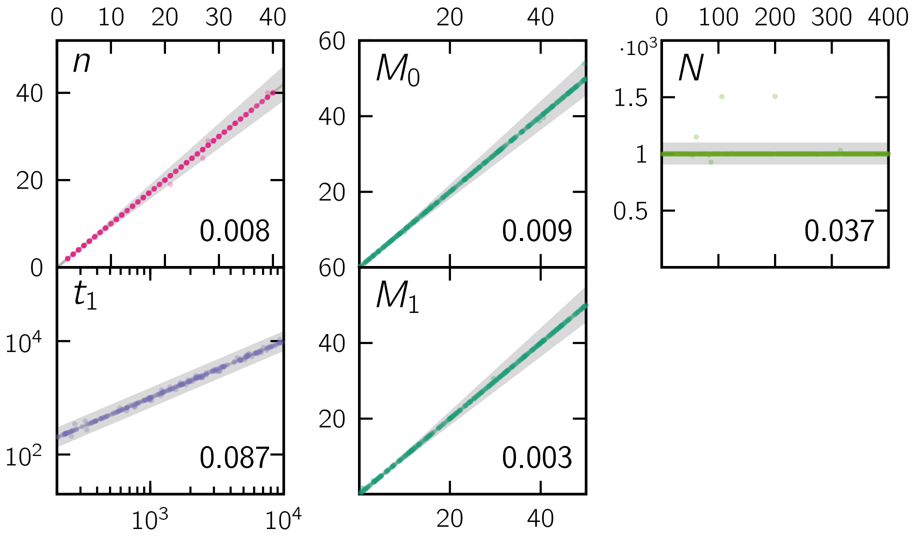

Figure S14: Scatter plots of all the simulated (horizontal axis) versus inferred (vertical axis) parameter values for scenarios of $c = 2$ components.

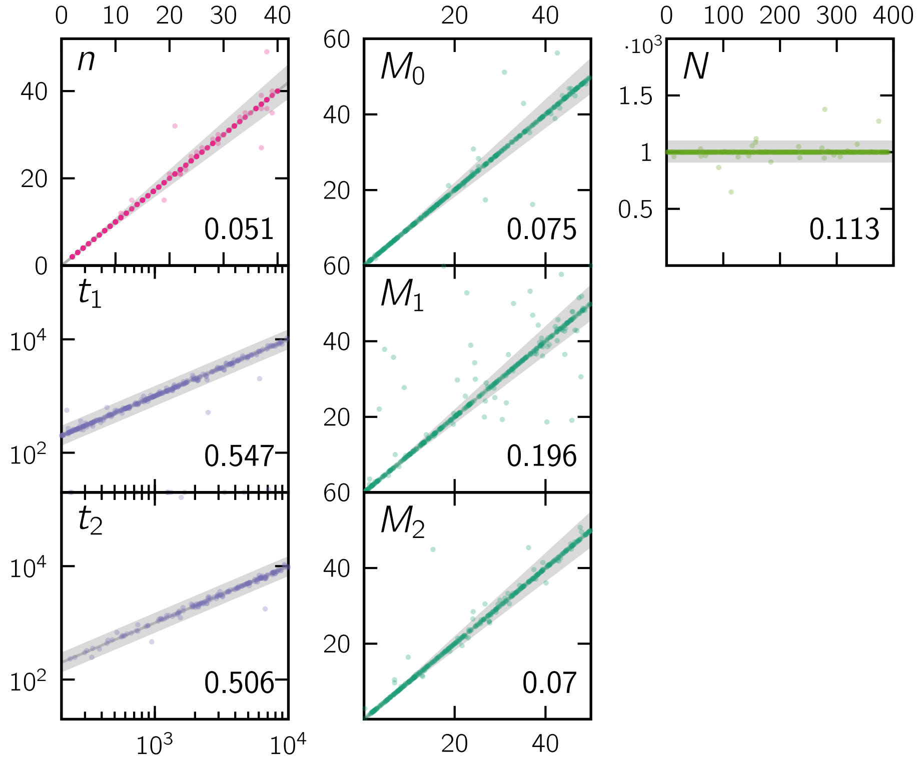

Figure S15: Scatter plots of all the simulated (horizontal axis) versus inferred (vertical axis) parameter values for scenarios of $c = 3$ components.

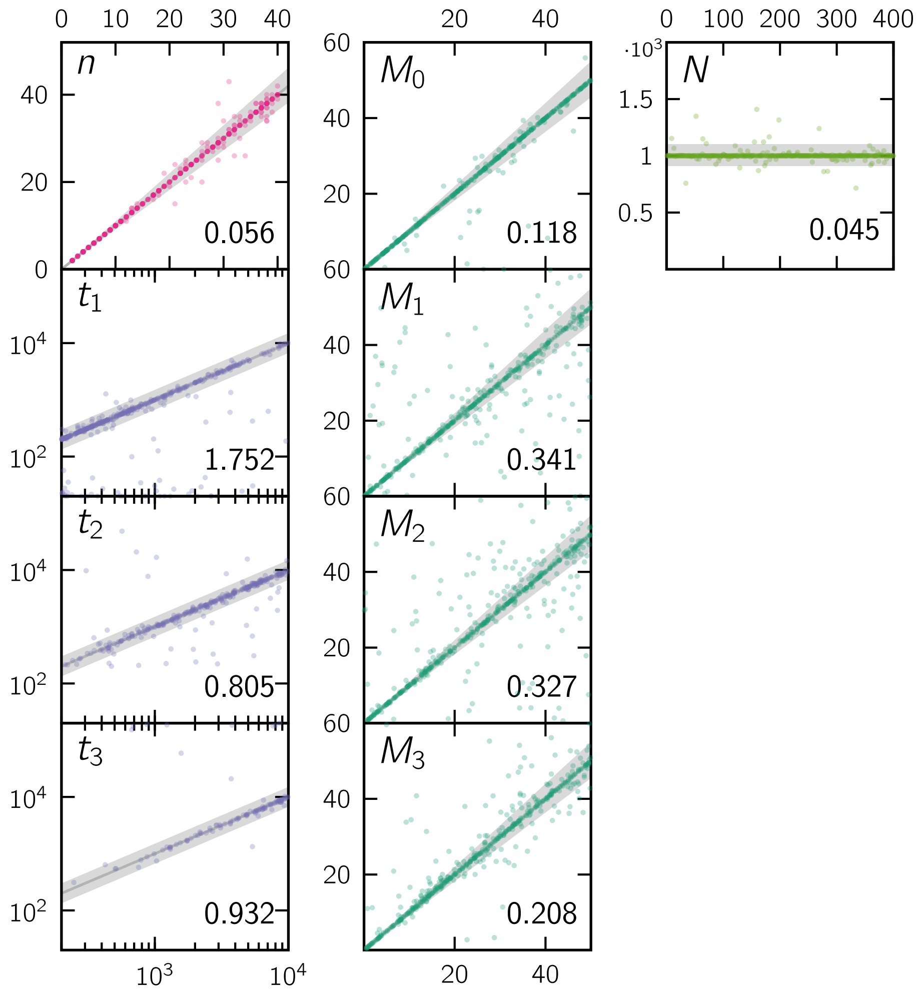

Figure S16: Scatter plots of all the simulated (horizontal axis) versus inferred (vertical axis) parameter values for scenarios of $c = 4$ components.

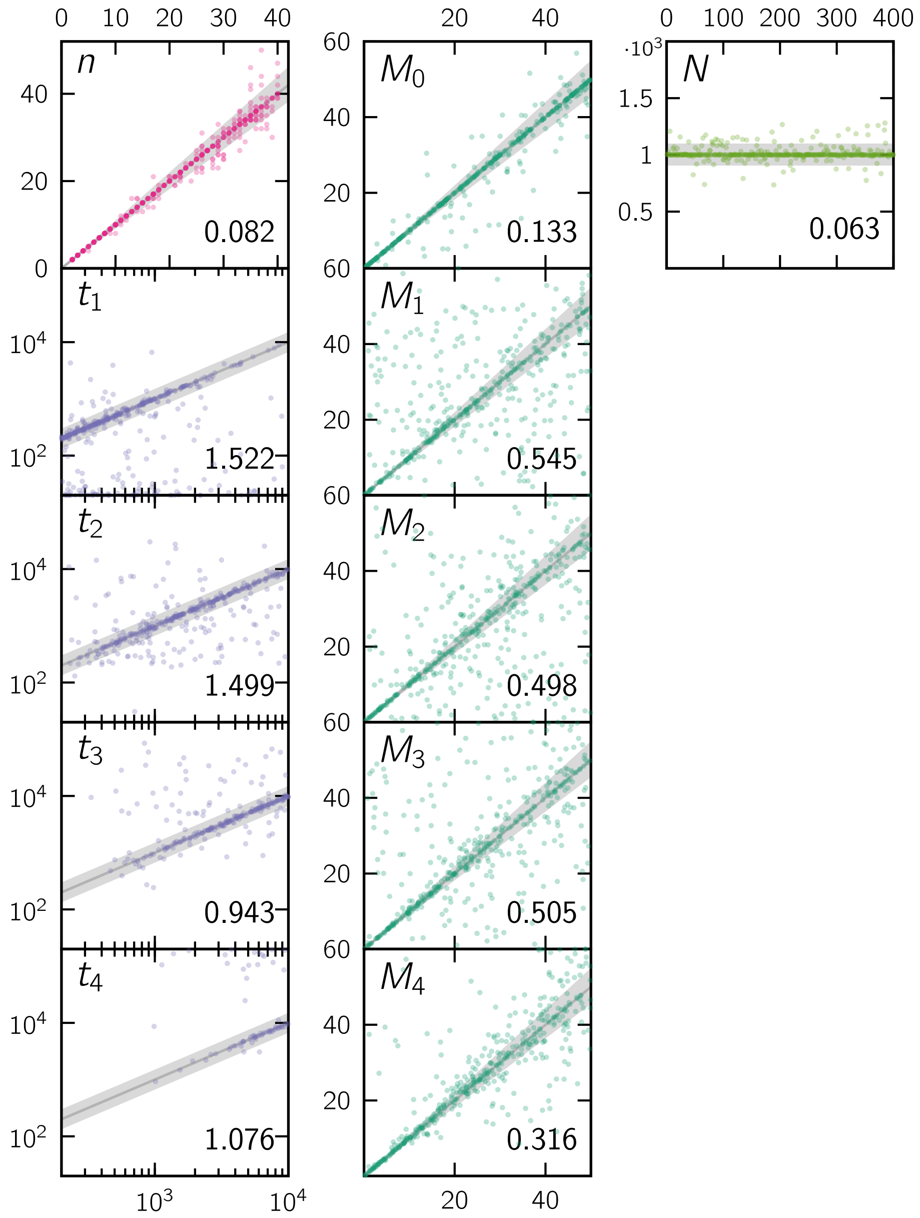

Figure S17: Scatter plots of all the simulated (horizontal axis) versus inferred (vertical axis) parameter values for scenarios of $c = 5$ components.

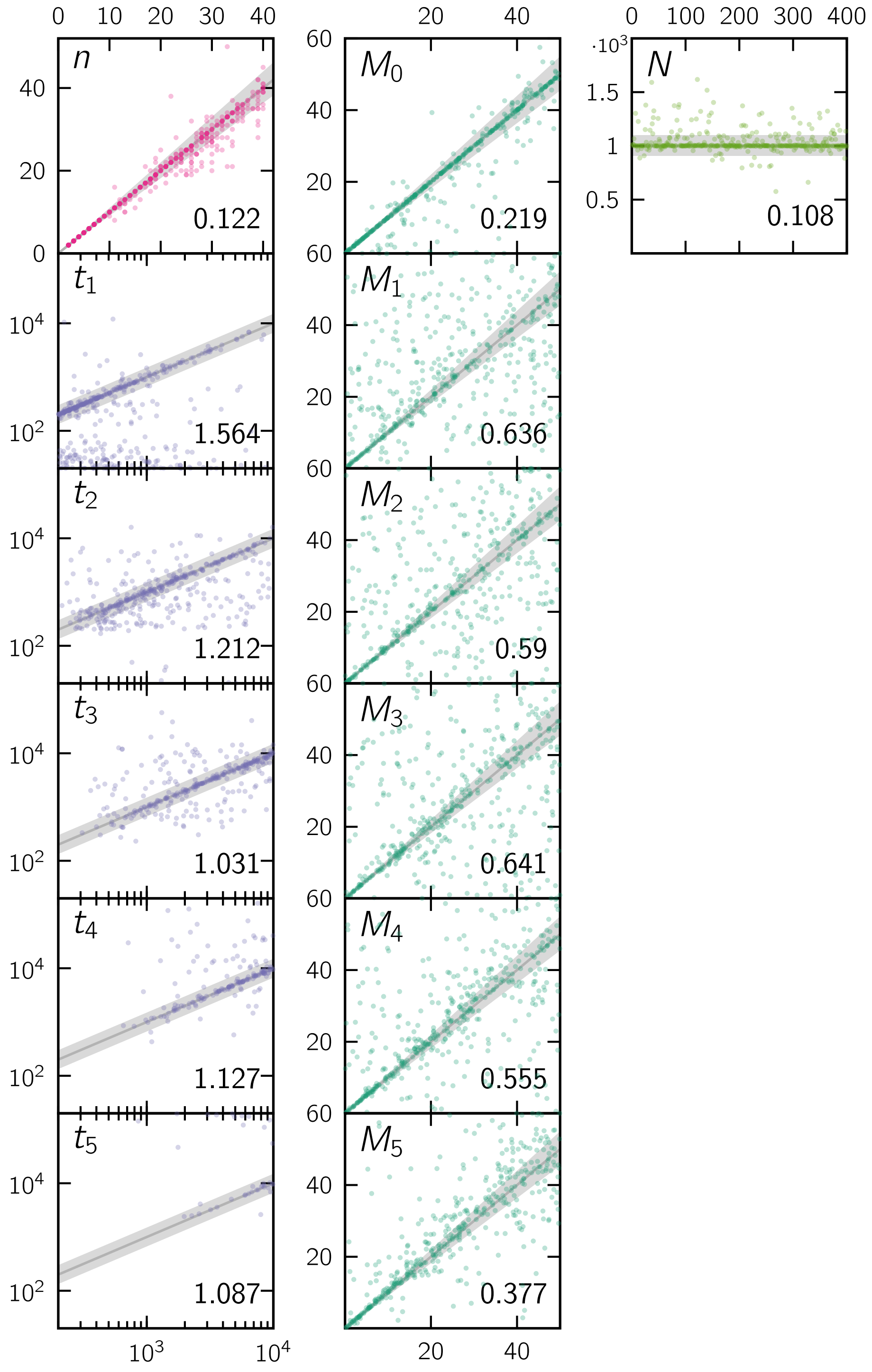

Figure S18: Scatter plots of all the simulated (horizontal axis) versus inferred (vertical axis) parameter values for scenarios of $c = 6$ components.

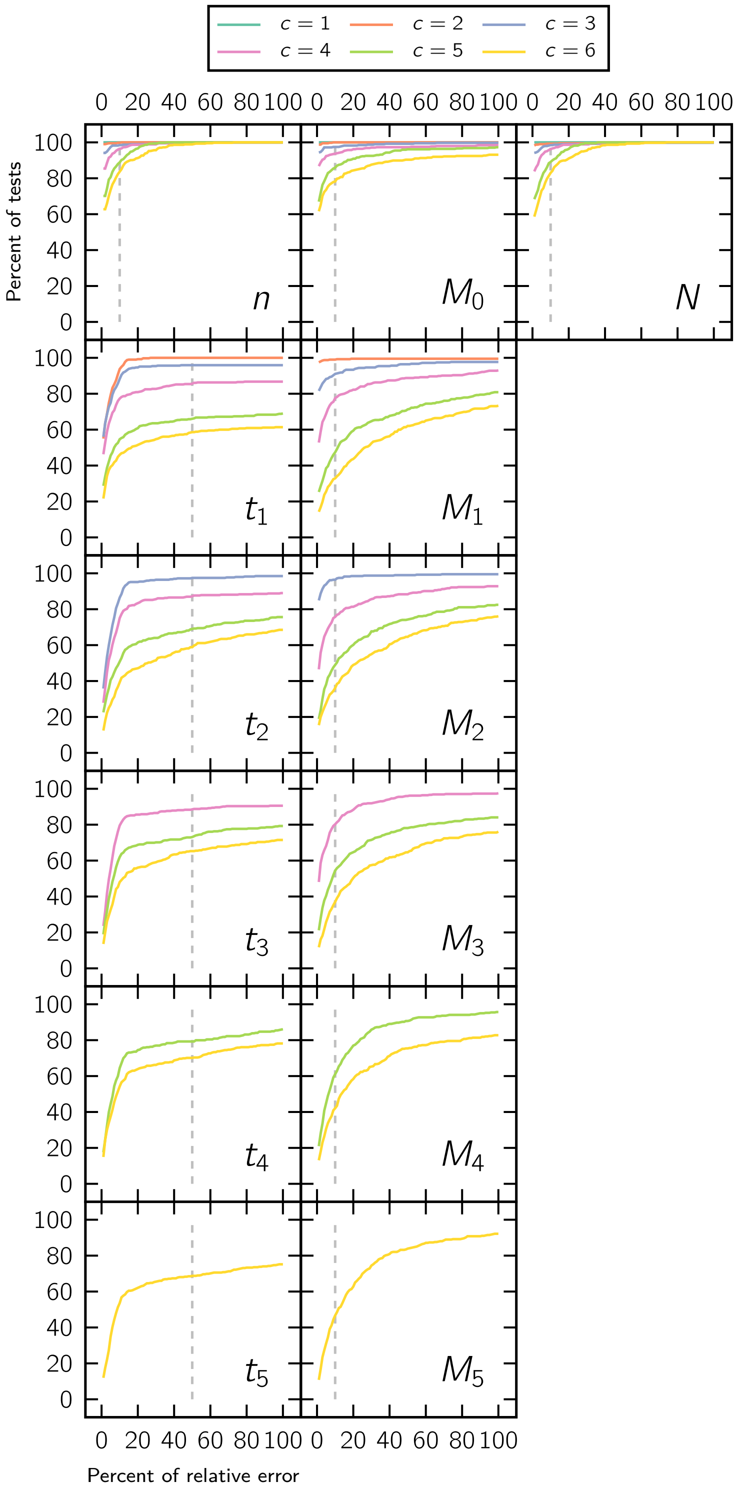

Figure S19: Percent of tests within a given relative error in scaled scenarios. For each parameter of the demographic scenarios, we counted the instances where the inferred value was within a certain percent of the simulated one. We say that an inferred parameter $\bar{p}$ is within x% of the simulated value p if $-x\hat{p} < 100(\hat{p} - p) < xp$. Note that not all parameters are present in all scenarios (for instance, the parameter M_4 only appears in scenarios with $c \geqslant 5$ components).

S3.4. Quantifying the inference error

In order to quantify the inference error incurred during the continuous-sampling validation phase, we measured the normalised root-mean-square deviation (nRMSD) between the simulated and inferred parameter values for each parameter of the demographic models ($c = 1$ to $c = 6$ components). These values can be seen in the lower-right corner of every sub-panel in figures S6 to S11 and S13 to S18. They are also summarised in Figure S20. We note that the number of islands n and the effective size N (in scaled scenarios) is very well inferred regardless of the number of components c. On the other hand, the inference accuracy of the connectivity rates M_i does get gradually worse when increasing the number of parameters (see section Validation using exact target IICRs in the main text).

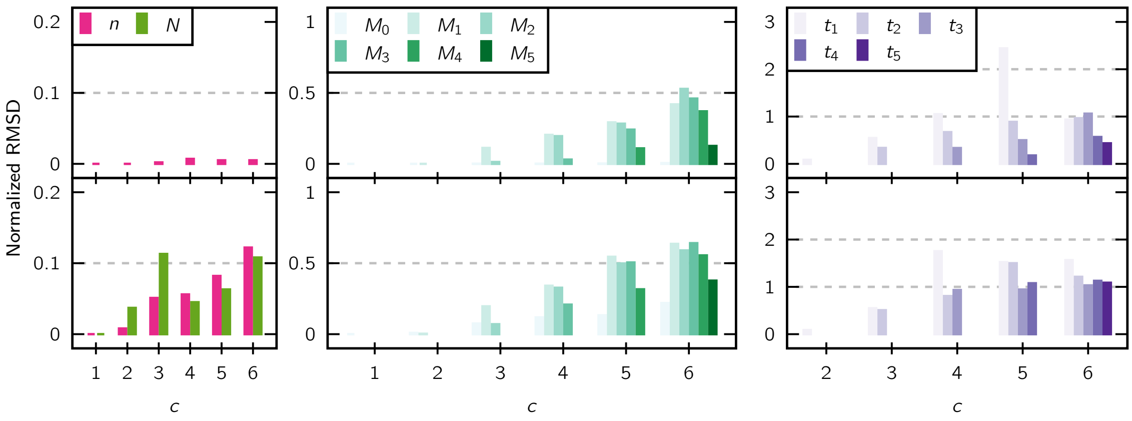

Figure S20: Normalised root-mean-square deviation of the inferred parameters during validation. The top part of each sub-panel corresponds to the parameters of unscaled scenarios, and the bottom part to scaled scenarios. The number of components c of the scenarios is in the horizontal axis in every case.

In an effort to quantify the component miss-identification phenomenon, we computed the correlation between 100 randomly sampled pairwise values of simulated and estimated parameters (in this case, the simulated M_2 parameter in unscaled scenarios of $c = 5$ components with the inferred M_j parameters of the same scenarios). The key insight underlying this test is that when a parameter is badly estimated, it may be due to the fact that the method estimated the value from the component that is either just before or just after it. Conversely, the value of M from other components should be non-correlated, since values are taken at random within the range of allowed values. Now, we do not know if the method used the component just before or just after, as this may change from simulation to simulation. To solve this problem we computed the correlation (r^2) by using either both values or just the value that was closest. We did exactly the same for the values taken from other components that are not expected to be correlated. We find indeed that when no correlation is expected there is no correlation, but when we take the best value the correlation increases, and it increases much more when it is a neighbouring value. Additionally, we found that this effect is significantly amplified (r^2 increased from 0.37 to 0.82) when we exclude from our sample the tests were the inferred rates were within 10% of the simulated values,

further indicating that this effect is present mostly when there is a large mismatch between the simulated and inferred M values. These results are displayed in Figure S21.

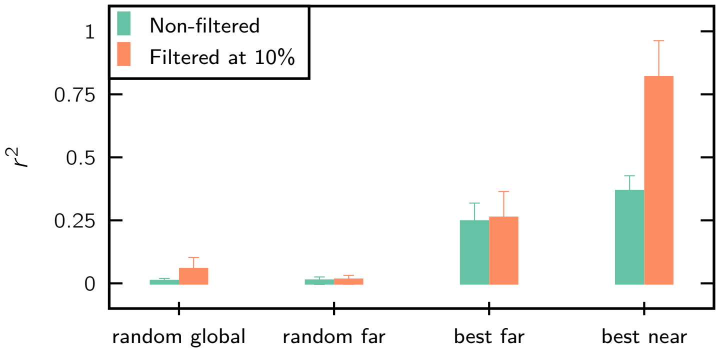

Figure S21: Parameter miss-identification. We measure the r^2 correlation between M_2 and $\hat{M}_j$ in a sample of 100 scenarios with $c = 5$ components. In 'random global' we draw M_j from components 0, 1, 3, and 4. In 'random far' we draw M_j from components 0 and 4. In 'best far' we draw M_j from components 0 and 4 again, but always keep the best fitting value of the two. Finally, in 'best near' we draw M_j from components 1 and 3 and always keep the best fitting value. 'Filtered at 10%' indicates that the scenarios where the inferred value was within 10% of the simulated value were excluded.

S4. Results of validation using T-sim IICRs

In this section we show the validation results of simulating and then inferring from 100 randomly generated demographic scenarios with scaled IICRs and varying number of components c. In all cases, the scenario parameters were drawn from the following finite sets:

$$\begin{aligned} n &\in \{2, 5, 10, 15, 20\}, \\ t_i &\in \{0.1, 0.5, 1, 2, 5, 10, 20, 50\} \quad \forall i, \\ M_i &\in \{0.1, 0.2, 0.5, 1, 2, 5, 10, 20, 50\} \quad \forall i, \\ s_i &= 1 \quad \forall i, \\ N &= 1000. \end{aligned} \tag{7}$$

Unlike in the previous sections, the simulated IICRs are T-sim IICRs, meaning that the values are not exact due to the stochastic nature of the underlying ms simulation. For each value of c from $c = 1$ to $c = 5$ we show the aggregate connectivity graph for all the simulations as well as the IICR and parameters of two individual scenarios from the set.

S4.1. Scenarios with 1 component

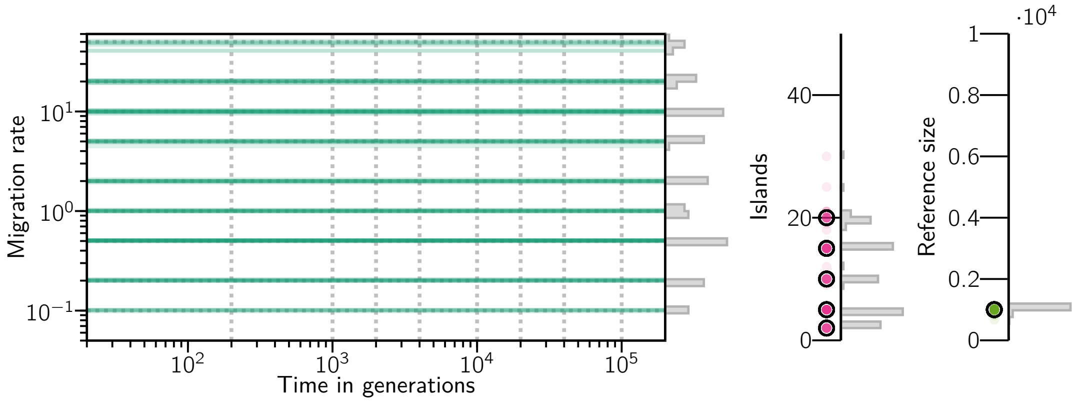

Figure S22: Connectivity graph of 100 inferred demographic histories using simulated T-sim IICRs of $c = 1$ components with simulated parameters randomly drawn from (7) and represented here by the dashed gray lines in the connectivity graph and the bold black circles in the islands and reference size plots.

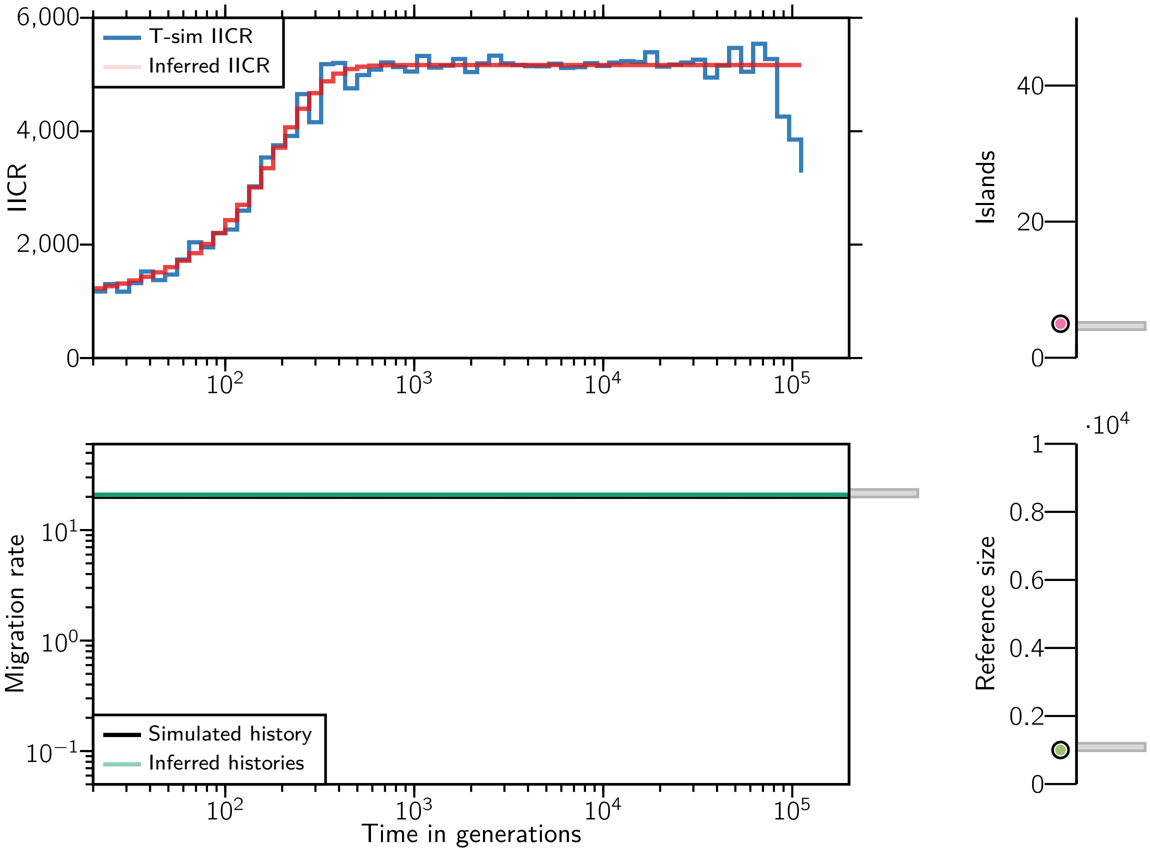

Figure S23: IICRs, connectivity graph, number of islands and reference size for one of the 100 simulated scenarios with $c = 1$ component and 10 independent inferences. The inferred scenario corresponds to $n = 5$, $M = 20$ and $N = 1000$.

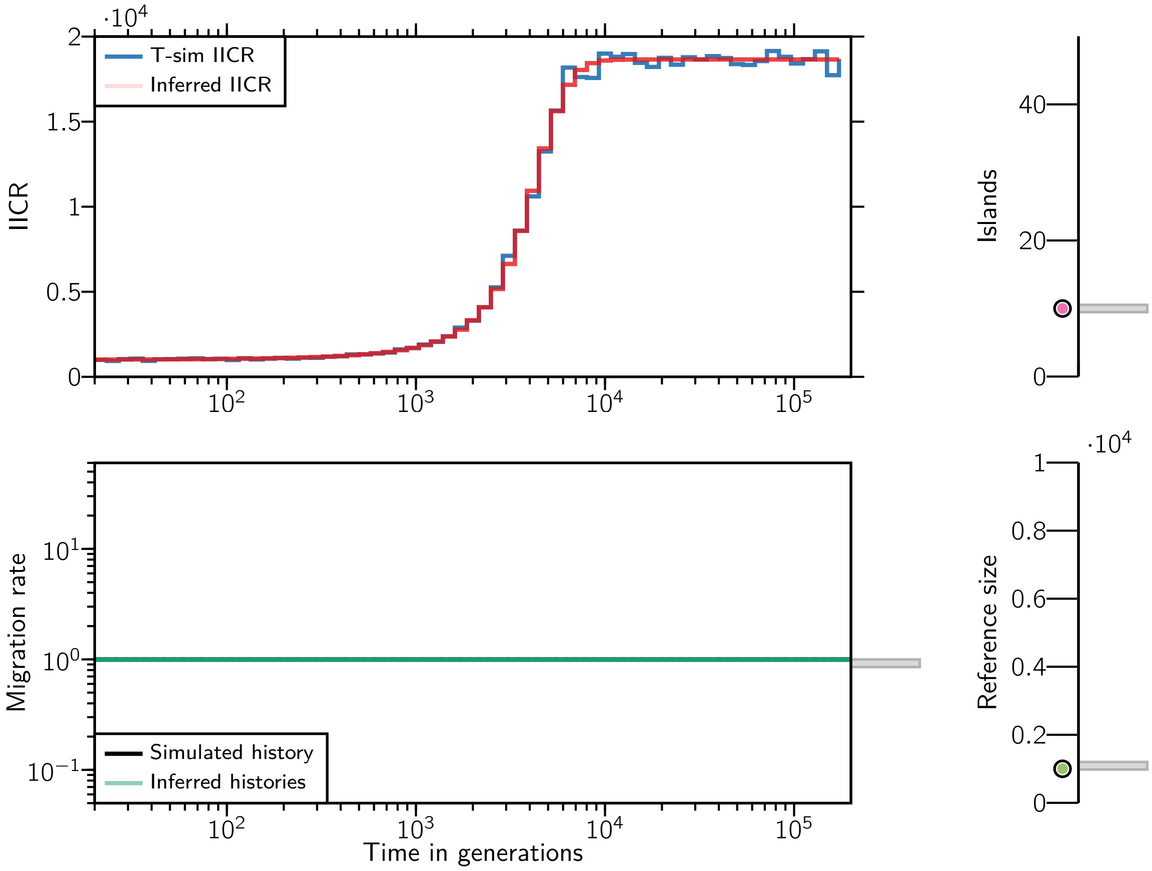

Figure S24: IICRs, connectivity graph, number of islands and reference size for one of the 100 simulated scenarios with $c = 1$ component and 10 independent inferences. The inferred scenario corresponds to $n = 10$, $M = 1$ and $N = 1000$.

S4.2. Scenarios with 2 components

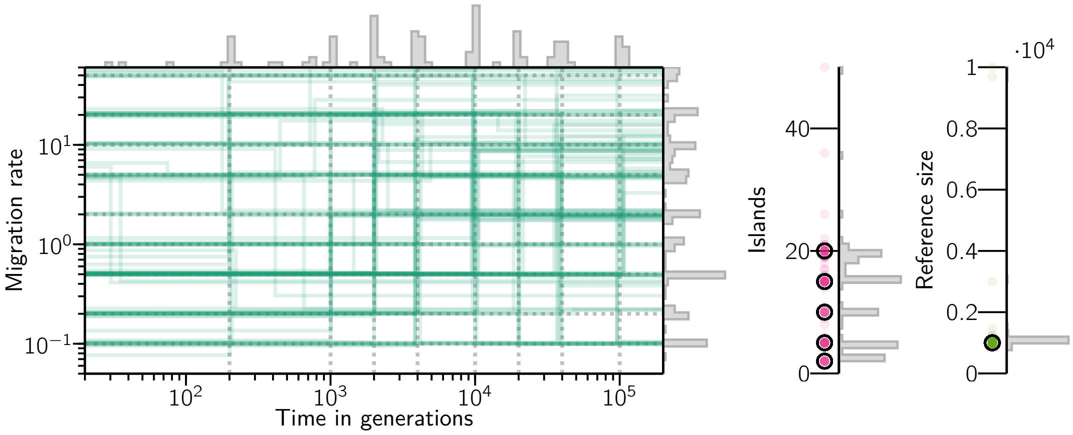

Figure S25: Connectivity graph of 100 inferred demographic histories using simulated T-sim IICRs of $c = 2$ components with simulated parameters randomly drawn from (7) and represented here by the dashed gray lines in the connectivity graph and the bold black circles in the islands and reference size plots.

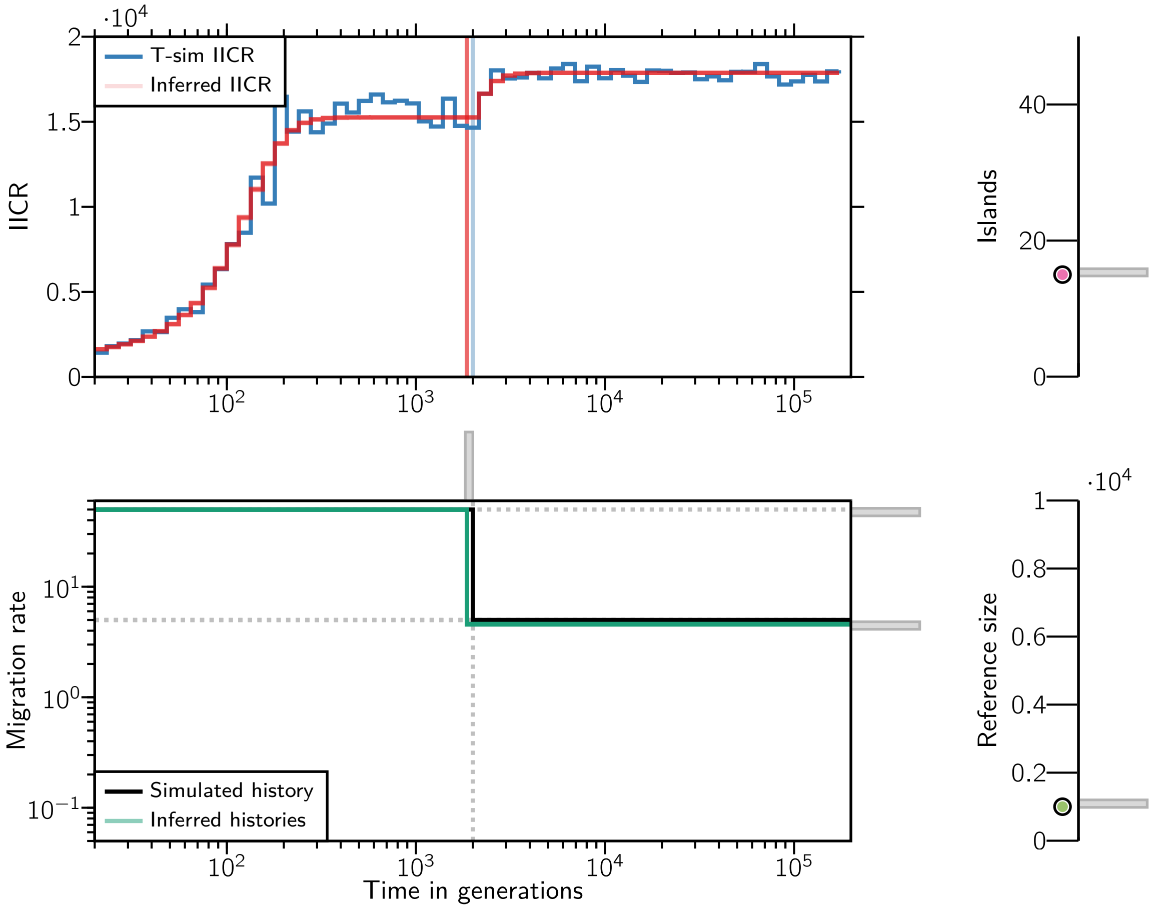

Figure S26: IICRs, connectivity graph, number of islands and reference size for one of the 100 simulated scenarios with $c = 2$ components and 10 independent inferences. The inferred scenario corresponds to $n = 15$, $t = 1$, $M = (50, 5)$ and $N = 1000$.

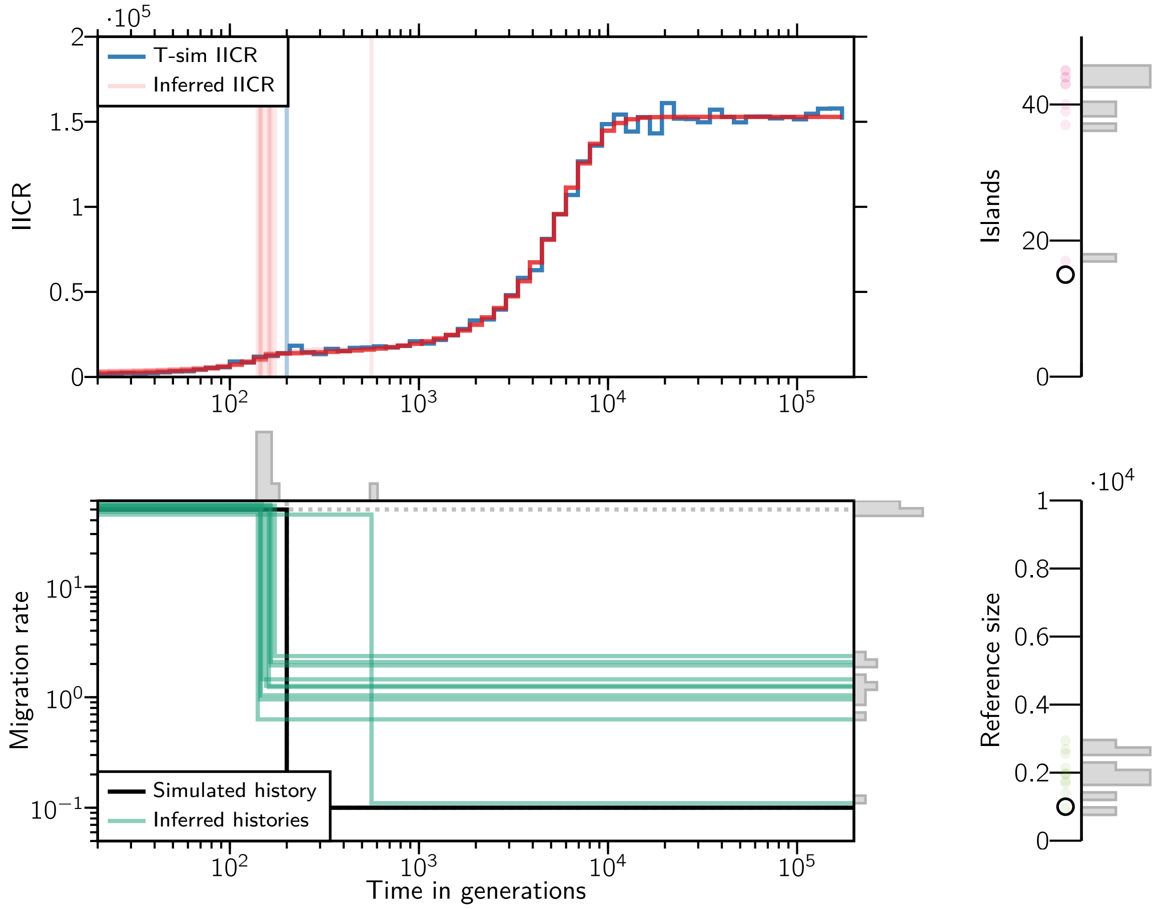

Figure S27: IICRs, connectivity graph, number of islands and reference size for one of the 100 simulated scenarios with $c = 2$ components and 10 independent inferences. The inferred scenario corresponds to $n = 15$, $t = 0.1$, $M = (50, 0.1)$ and $N = 1000$.

S4.3. Scenarios with 3 components

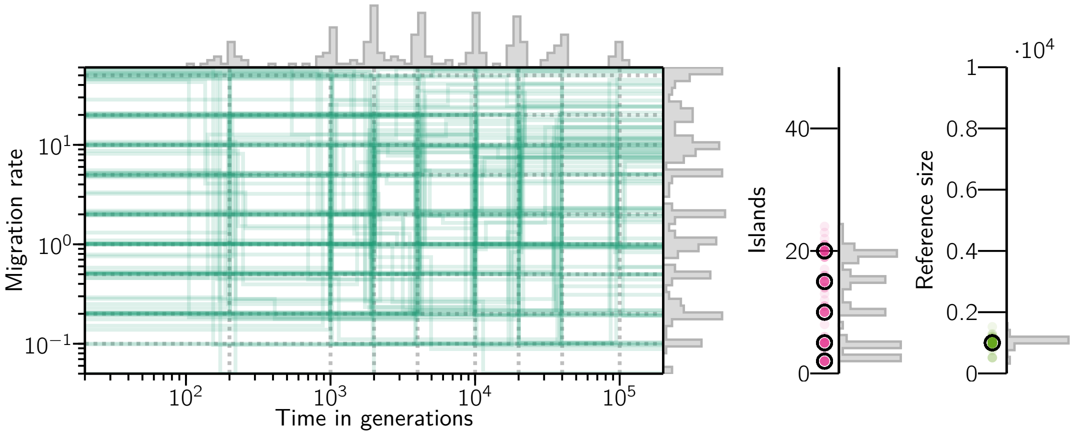

Figure S28: Connectivity graph of 100 inferred demographic histories using simulated T-sim IICRs of $c = 3$ components with simulated parameters randomly drawn from (7) and represented here by the dashed gray lines in the connectivity graph and the bold black circles in the islands and reference size plots.

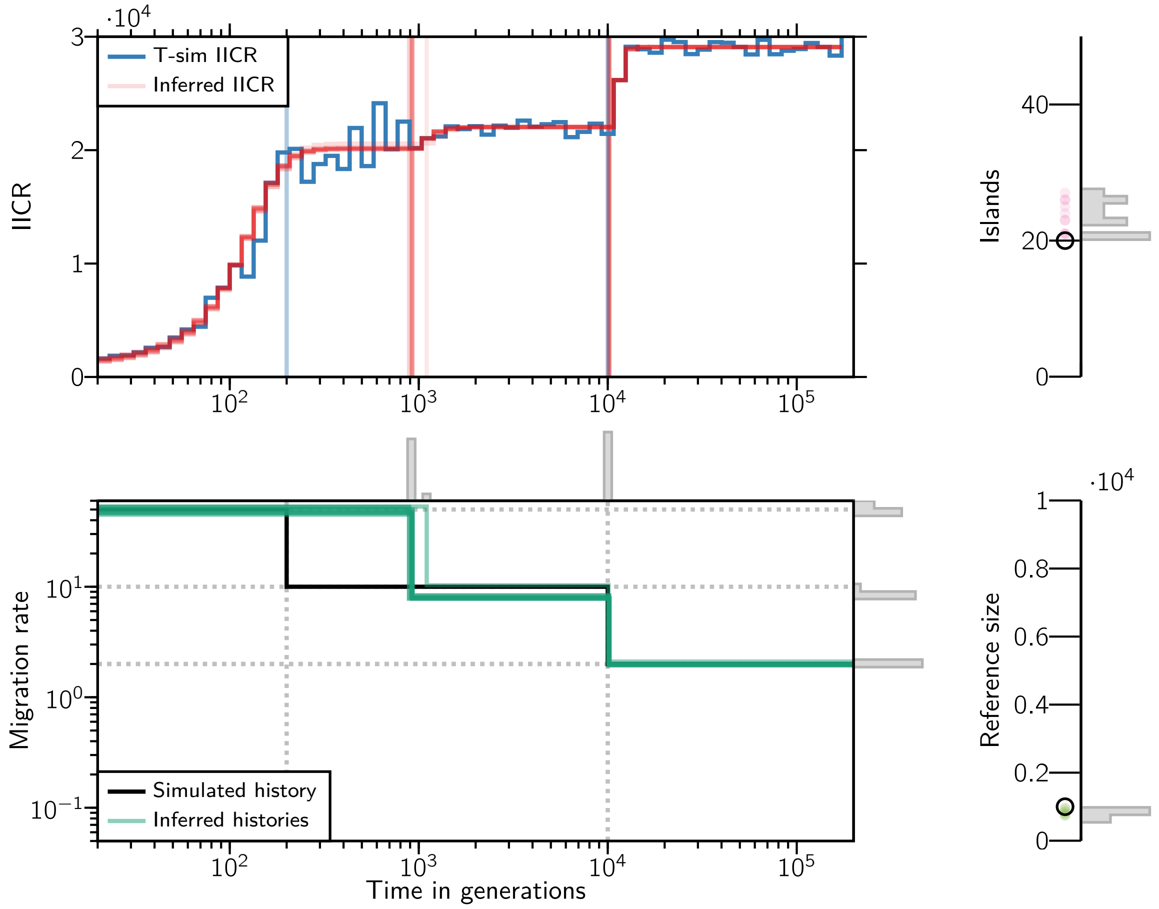

Figure S29: IICRs, connectivity graph, number of islands and reference size for one of the 100 simulated scenarios with $c = 3$ components and 10 independent inferences. The inferred scenario corresponds to $n = 20$, $t = (0.1, 5)$, $M = (50, 10, 2)$ and $N = 1000$.

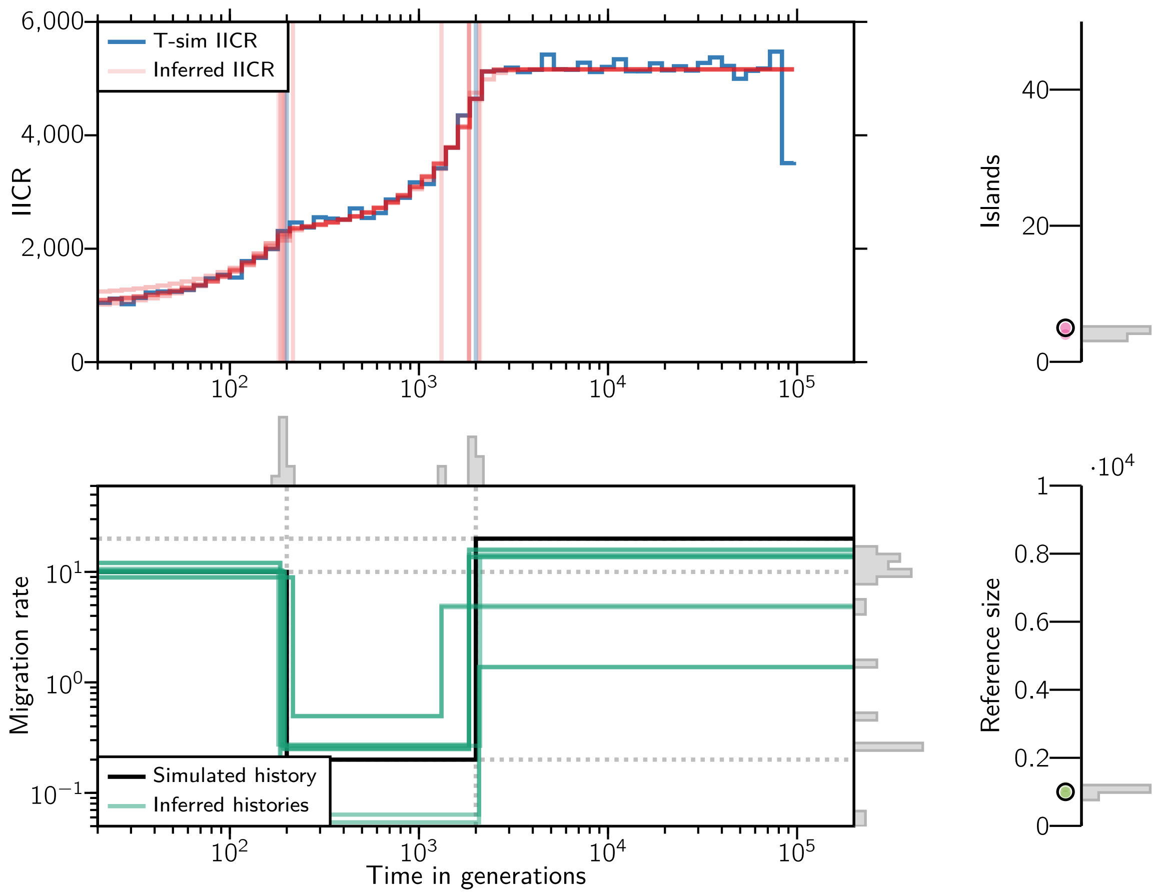

Figure S30: IICRs, connectivity graph, number of islands and reference size for one of the 100 simulated scenarios with $c = 3$ components and 10 independent inferences. The inferred scenario corresponds to $n = 5$, $t = (0.1, 1)$, $M = (10, 0.2, 20)$ and $N = 1000$.

S4.4. Scenarios with 4 components

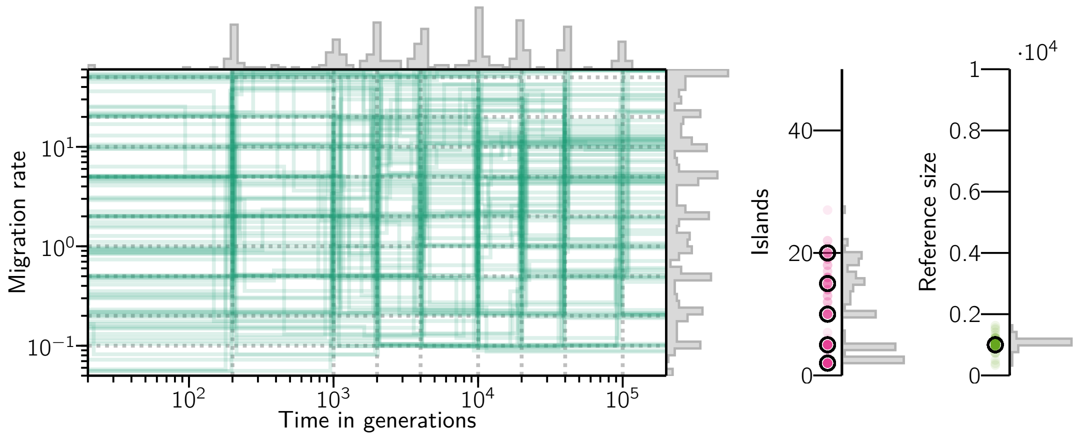

Figure S31: Connectivity graph of 100 inferred demographic histories using simulated T-sim IICRs of $c = 4$ components with simulated parameters randomly drawn from (7) and represented here by the dashed gray lines in the connectivity graph and the bold black circles in the islands and reference size plots.

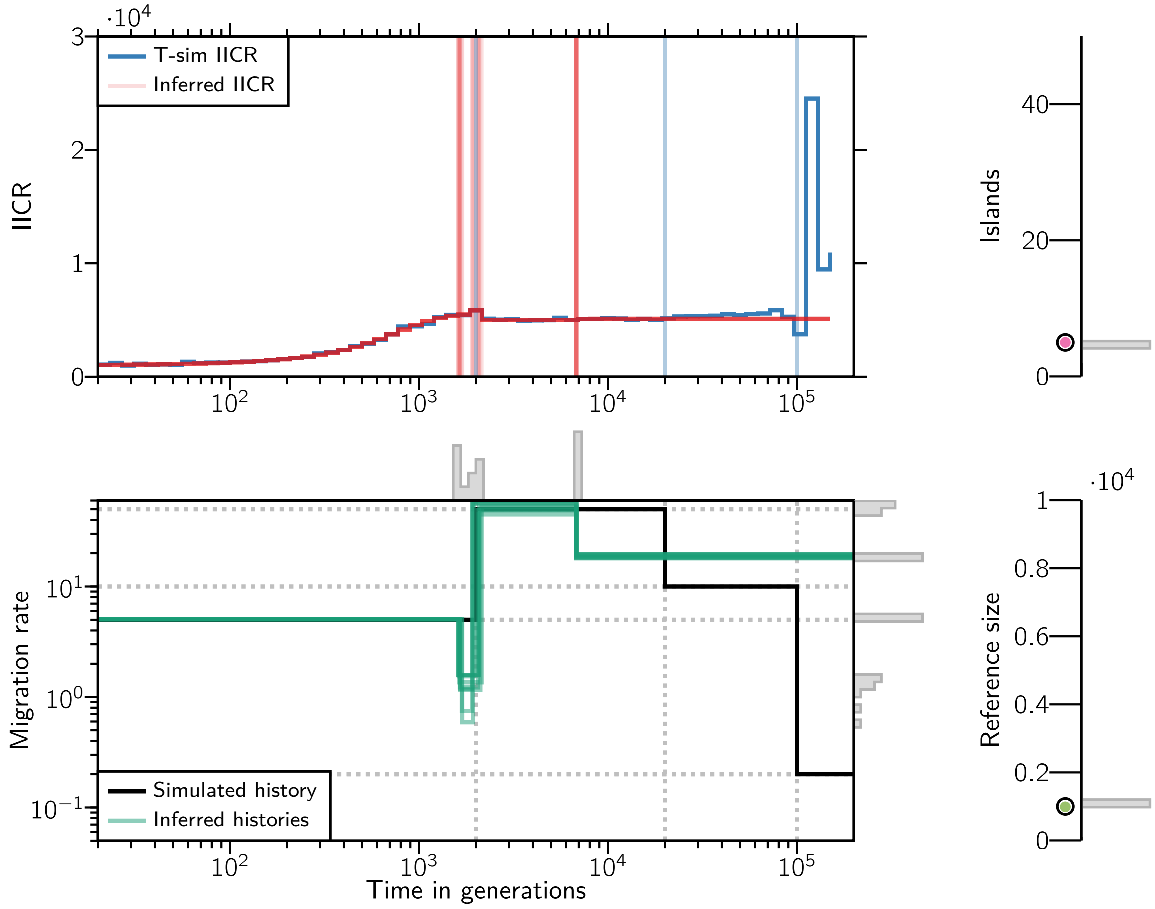

Figure S32: IICRs, connectivity graph, number of islands and reference size for one of the 100 simulated scenarios with $c = 4$ components and 10 independent inferences. The inferred scenario corresponds to $n = 5$, $t = (1, 10, 50)$, $M = (5, 50, 10, 0.2)$ and $N = 1000$.

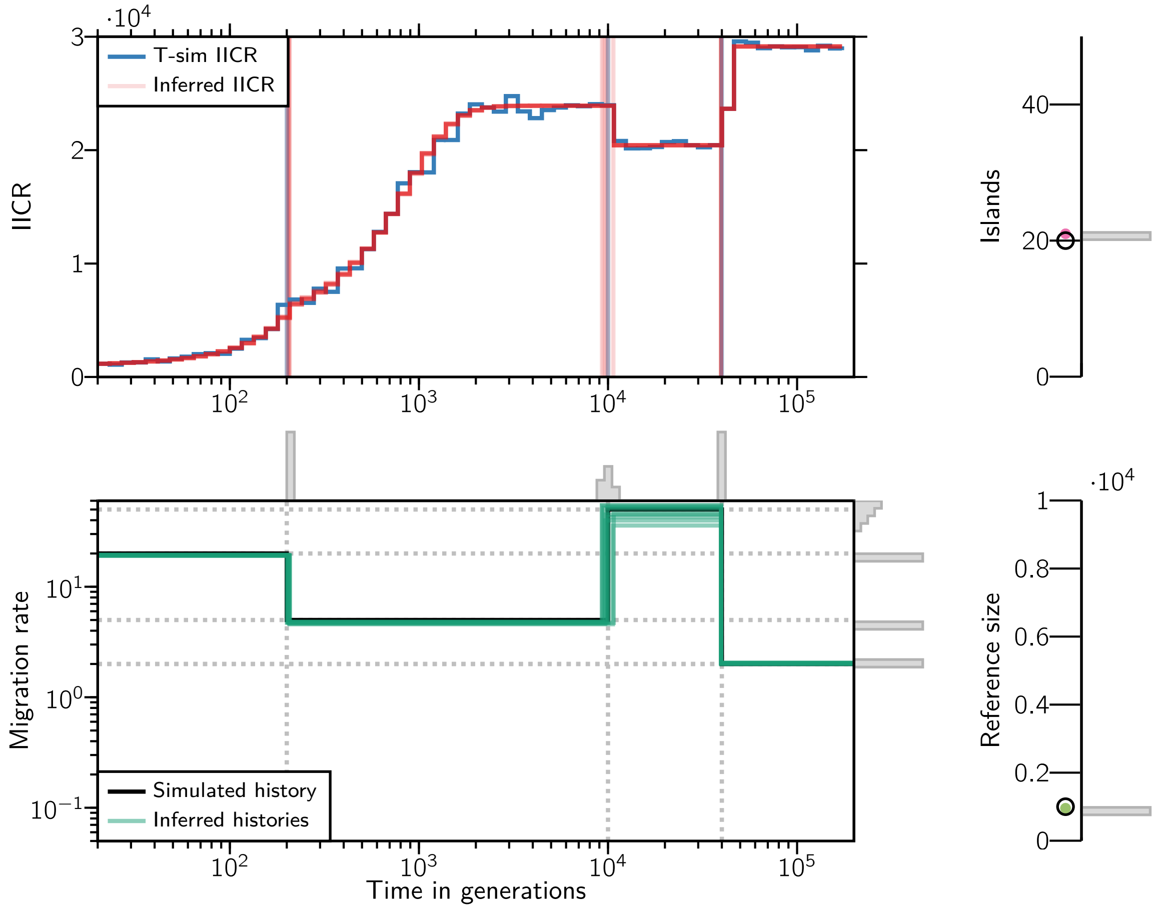

Figure S33: IICRs, connectivity graph, number of islands and reference size for one of the 100 simulated scenarios with $c = 4$ components and 10 independent inferences. The inferred scenario corresponds to $n = 20$, $t = (0.1, 5, 20)$, $M = (20, 5, 50, 2)$ and $N = 1000$.

S4.5. Scenarios with 5 components

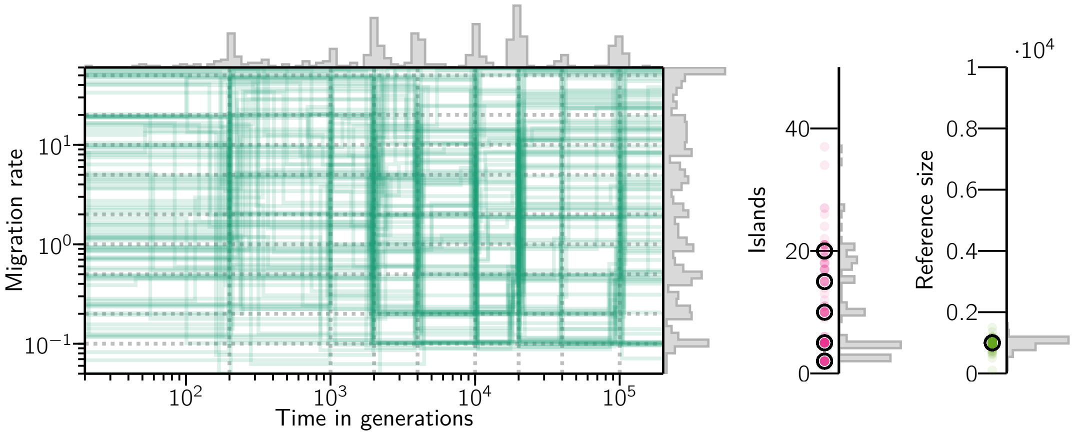

Figure S34: Connectivity graph of 100 inferred demographic histories using simulated T-sim IICRs of $c = 5$ components with simulated parameters randomly drawn from (7) and represented here by the dashed gray lines in the connectivity graph and the bold black circles in the islands and reference size plots.

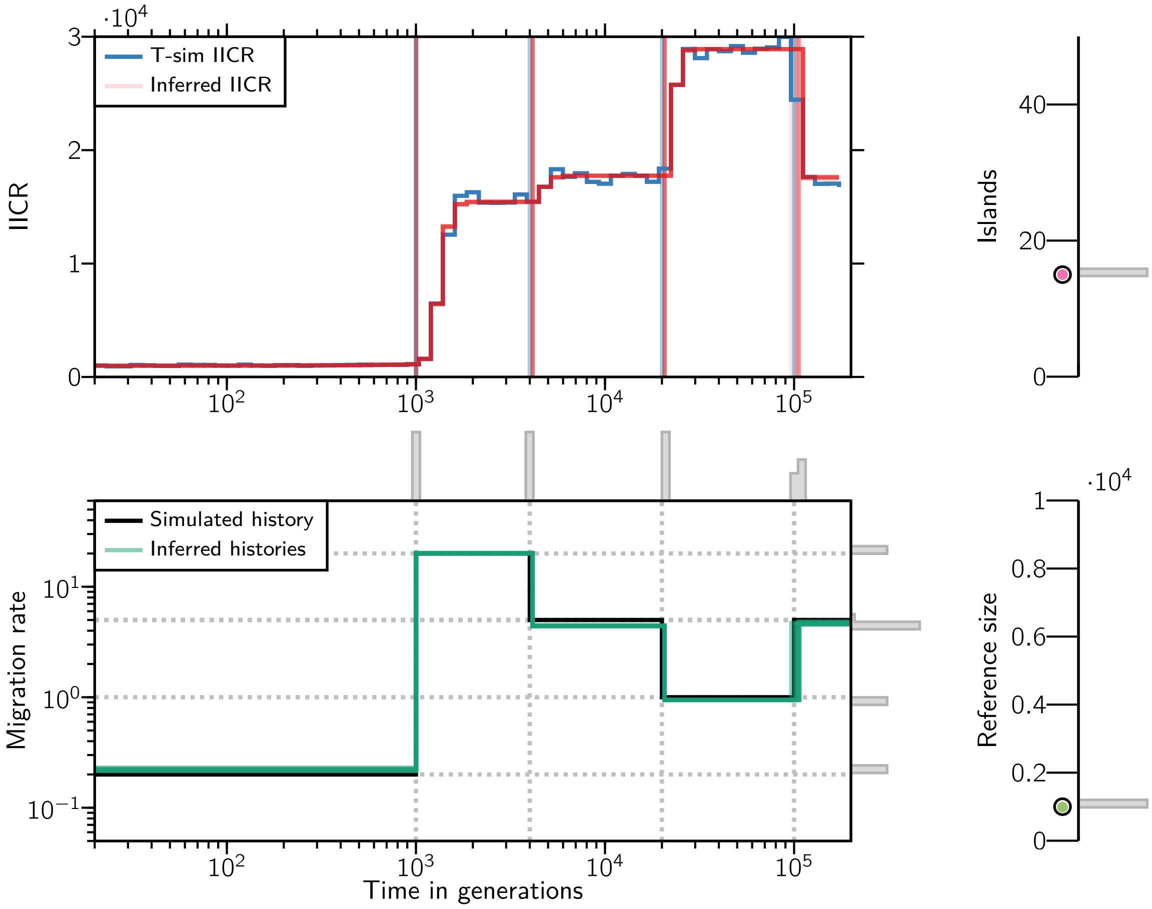

Figure S35: IICRs, connectivity graph, number of islands and reference size for one of the 100 simulated scenarios with $c = 5$ components and 10 independent inferences. The inferred scenario corresponds to $n = 15$, $t = (0.5, 2, 10, 50)$, $M = (0.2, 20, 5, 1, 5)$ and $N = 1000$.

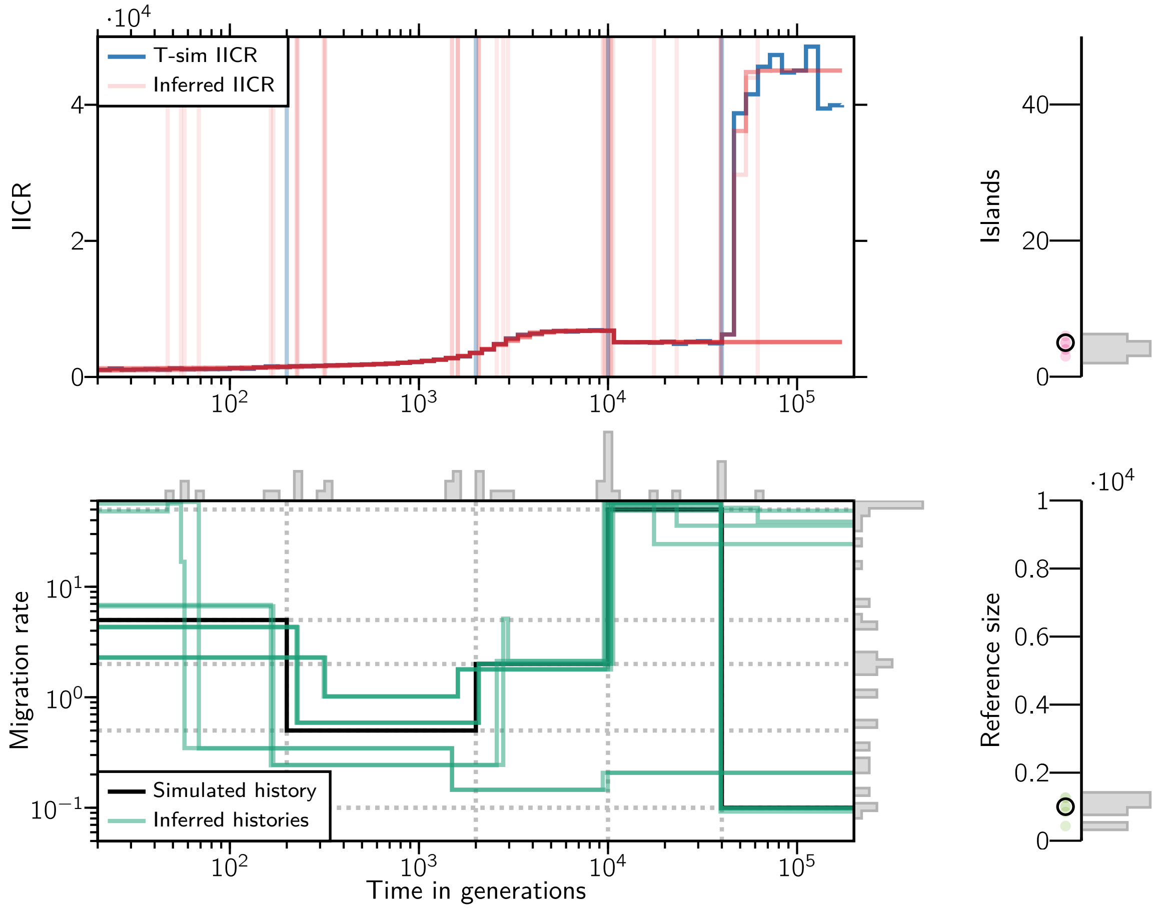

Figure S36: IICRs, connectivity graph, number of islands and reference size for one of the 100 simulated scenarios with $c = 5$ components and 10 independent inferences. The inferred scenario corresponds to $n = 5$, $t = (0.1, 1, 5, 20)$, $M = (5, 0.5, 2, 50, 0.1)$ and $N = 1000$.

S5. Results of application to human data

Figure S37 shows the IICR curves of the five human samples, scaled using a mutation rate of $\mu = 1.25 \times 10^{-8}$ and a generation time of 25 years (see section Application to humans in the main text).

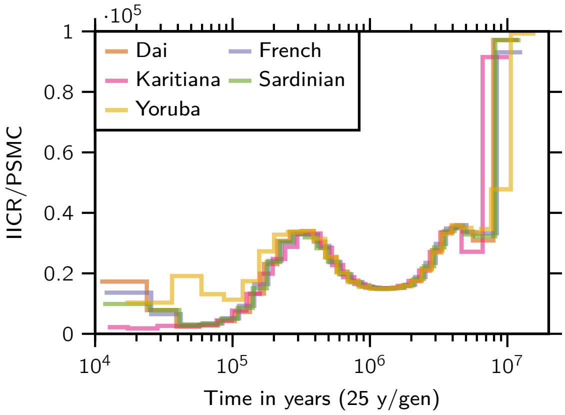

Figure S37: Five human PSMC plots. All five PSMC curves were obtained from the study of Prado-Martinez et al. (2013). The scaling was done as recommended by Li and Durbin (2011) using a value of $\mu = 1.25 \times 10^{-8}$ and a generation time of 25 years. No further processing was necessary since *.psmc* files contain the final result of the effective size or IICR estimate.

S5.1. Seq-Sim validations

Figure S39 shows the results of the validations using seq-sim IICRs. For the three chosen representative human populations (French, Karitiana and Yoruba), we selected the SNIF inferences obtained with the parameter values $c = 5$ components and $\omega = 0.2$. The selection of these values as the preferred ones was supported by the fact that the d_{visual} distance (see Figure S46) was best for them. For each of these inferred models we generated two independent genomic sequences of length 3×10^9 base pairs and applied the PSMC method to obtain two independently estimated target IICRs. These seq-sim IICRs are shown in blue on the left panels of Figure S39. The connectivity graphs associated with these inferred models are represented in the middle panels by the red curves. The corresponding inferred values of n and N are marked as the black reference circles in the right panels of the figure.

After obtaining the seq-sim IICRs, we applied again our inference method. The goal is to validate whether it would be able to consistently infer the same parameter values of the demographic history regardless of the origin of the source IICR curve. To this end we performed 10 independent inferences from each of the two target IICRs. The inferred IICRs are superimposed on the left panels of Figure S39 (transparent red curves, 20 per population). The inferred connectivity graphs are shown in the middle panels (transparent green curves) and the inferred values of n and N are presented in the right panels. We observe an agreement with the previously inferred histories, which suggests that *if* the real history of human evolution

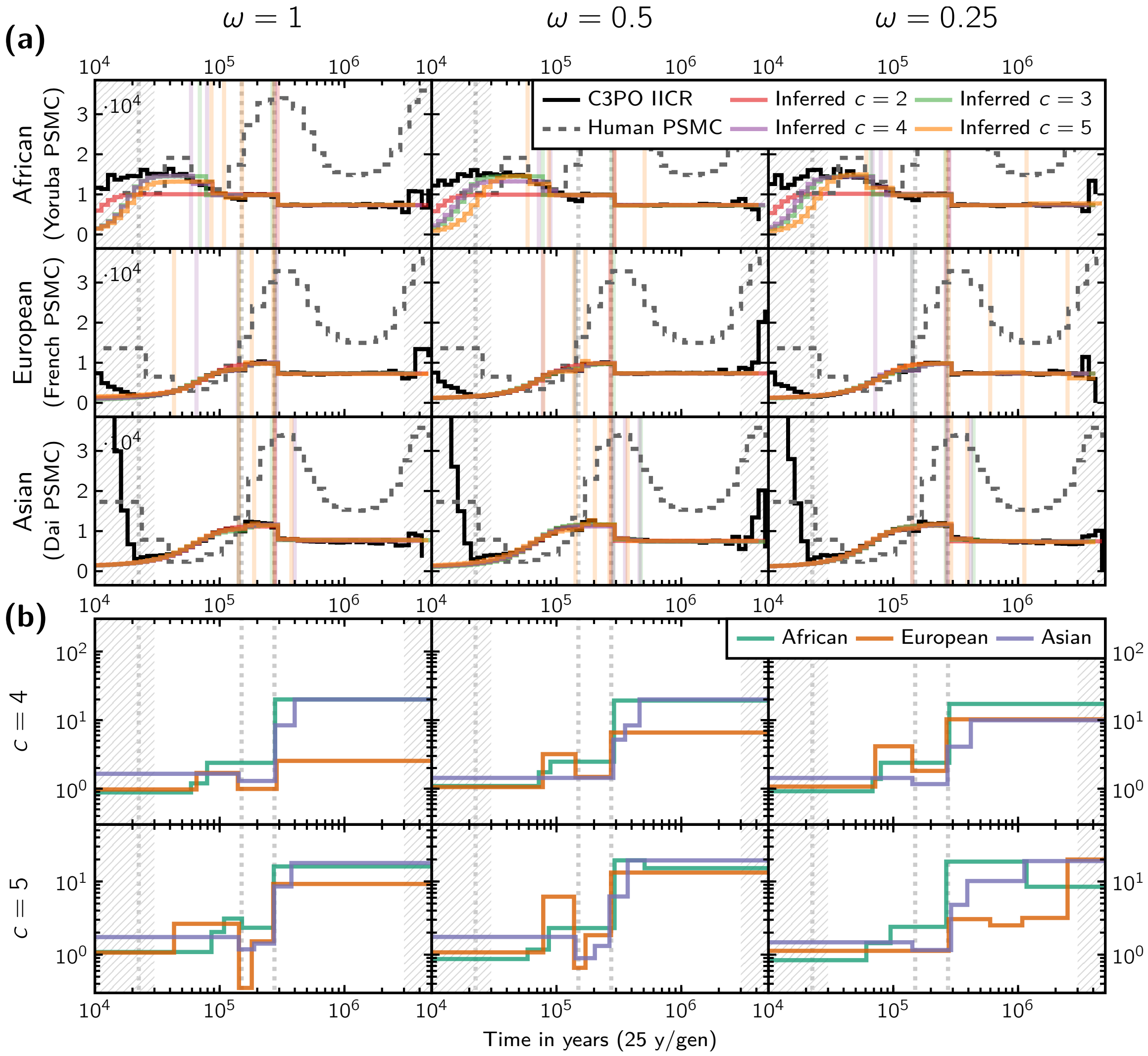

Figure S38: Application of our inference method to a tree-like human demographic scenario with three modern populations. **(a)** IICR plots showing the resulting IICR curve of the European population under this model and the inferred IICR curve obtained with our method (where the recent period of human expansion was ignored) for $c = 5$ components and a weight parameter of $\omega = 0.25$. For reference purposes, we also show the real PSMC curve of the French individual. The grey vertical lines indicate the inferred event times in the C3PO model, and the colored vertical lines the inferred event times by SNIF **(b)** Connectivity graph of the inferred scenario. For reference, we show the inferred event times in the C3PO model as grey vertical lines.

were close to piecewise n-island models like those used in this work, our method would be able to infer the parameters properly, and they would be similar to those shown in Figures 5 and 6 of the main text.

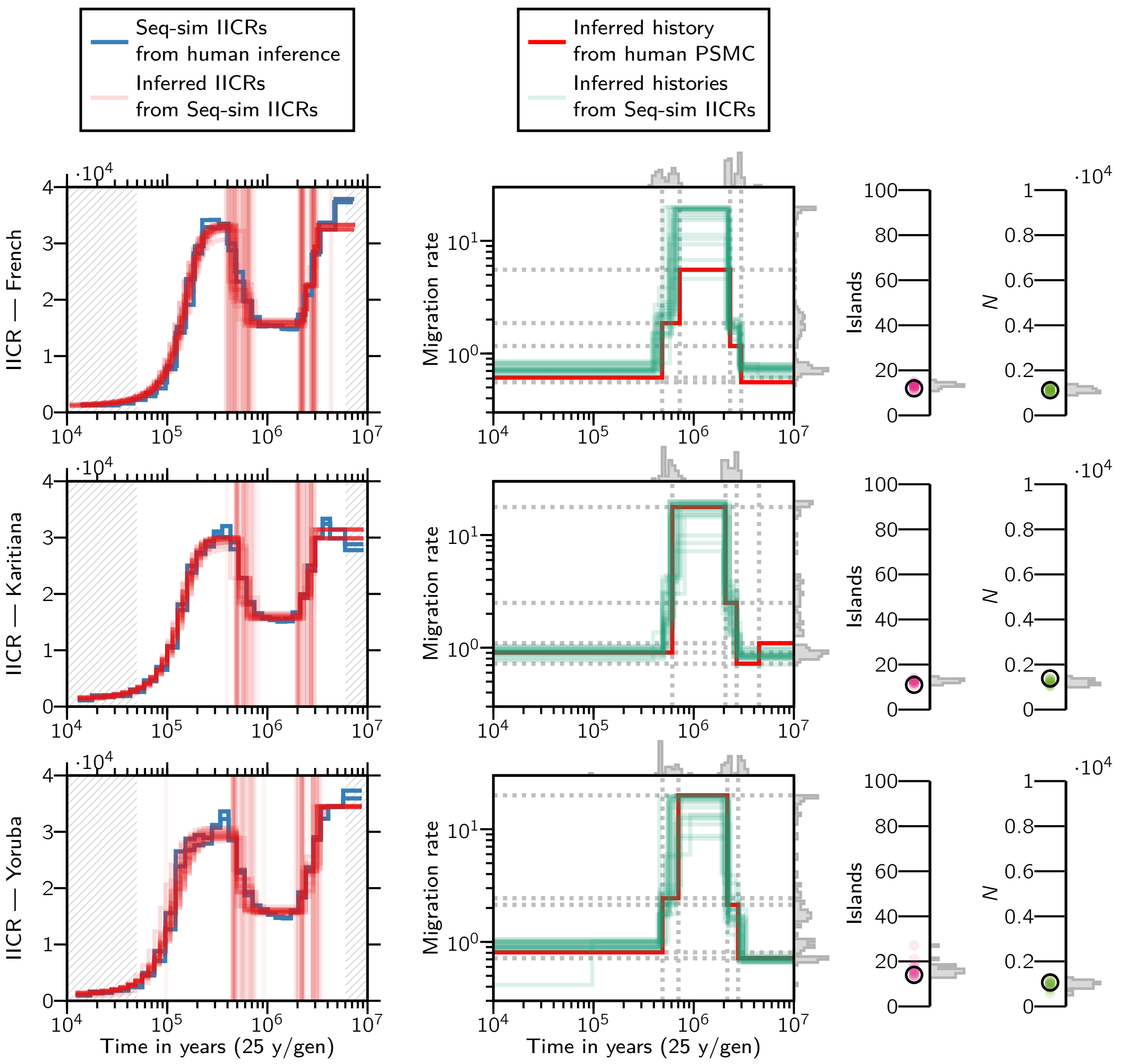

Figure S39: Results of the validation using Seq-sim IICRs. The left panels show two independently simulated seq-sim IICRs (obtained using the demographic scenario inferred with $c = 5$ and $\omega = 0.2$ for each of the indicated human population) alongside 10 independent inferred IICRs. The rest of the panels show the connectivity graphs, number of islands, and local deme size of these seq-sim IICRs and their corresponding inferences.

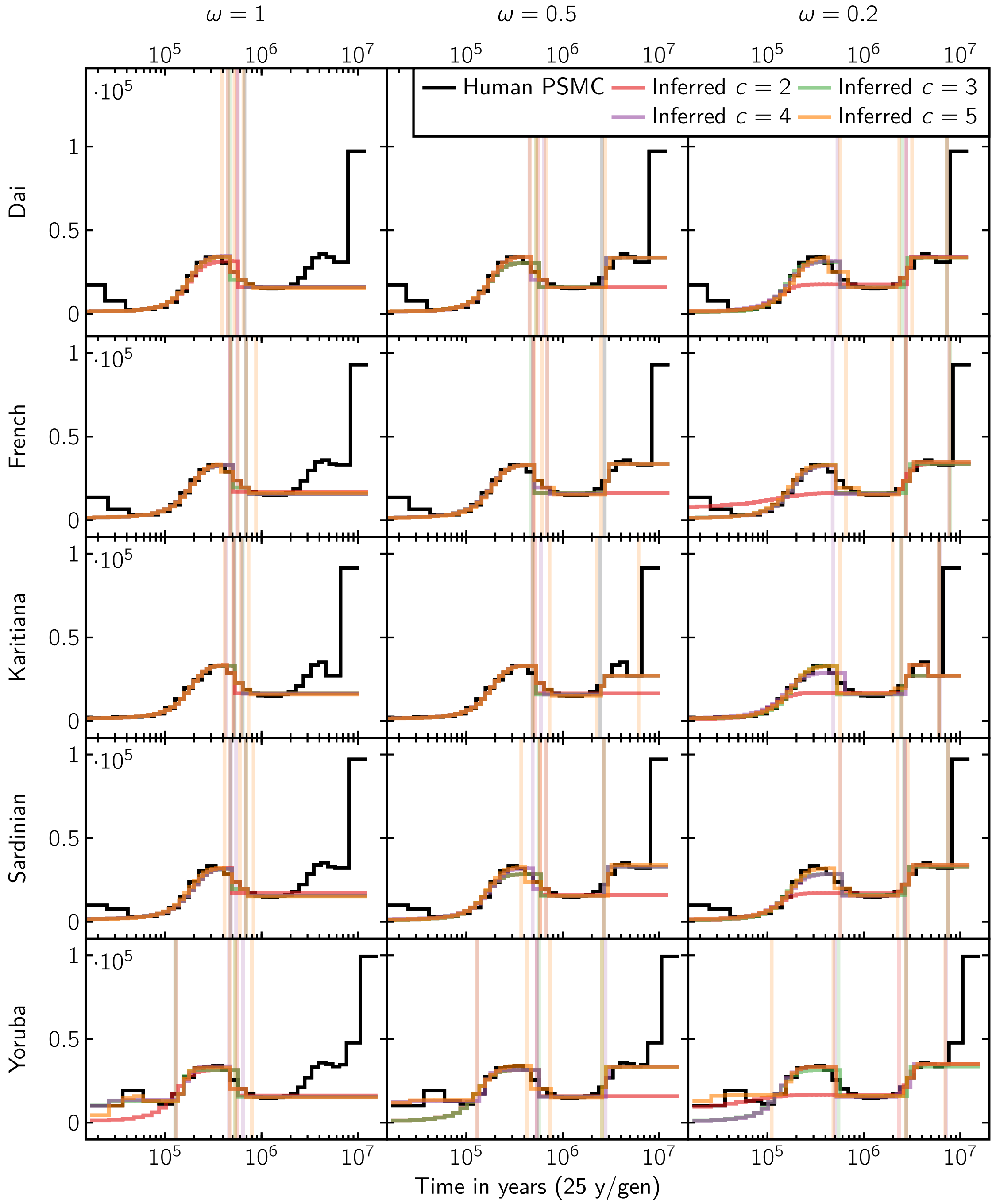

Figure S40: IICRs of the inferred human scenarios.

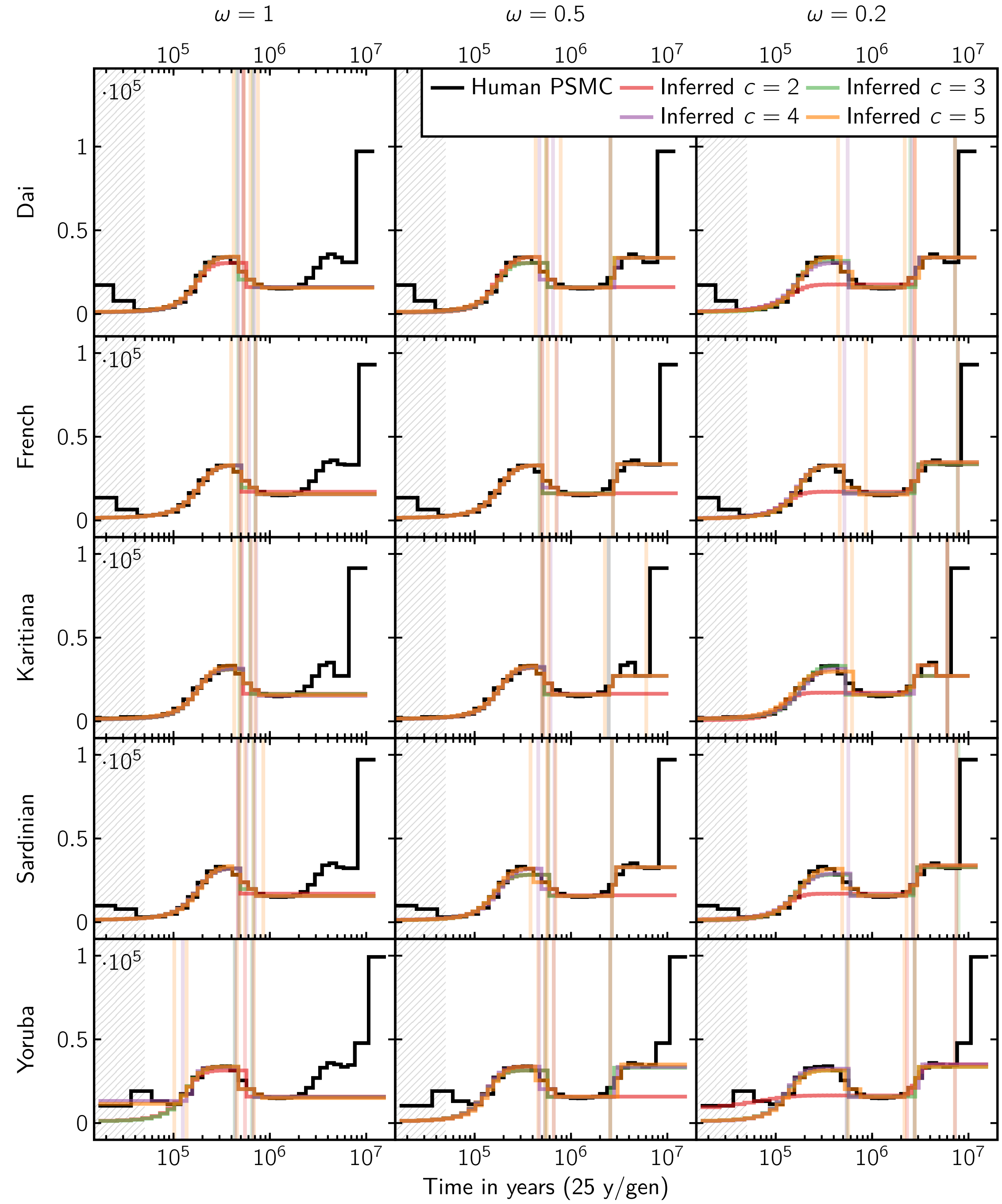

Figure S41: IICRs of the inferred human scenarios with restricted inference range.

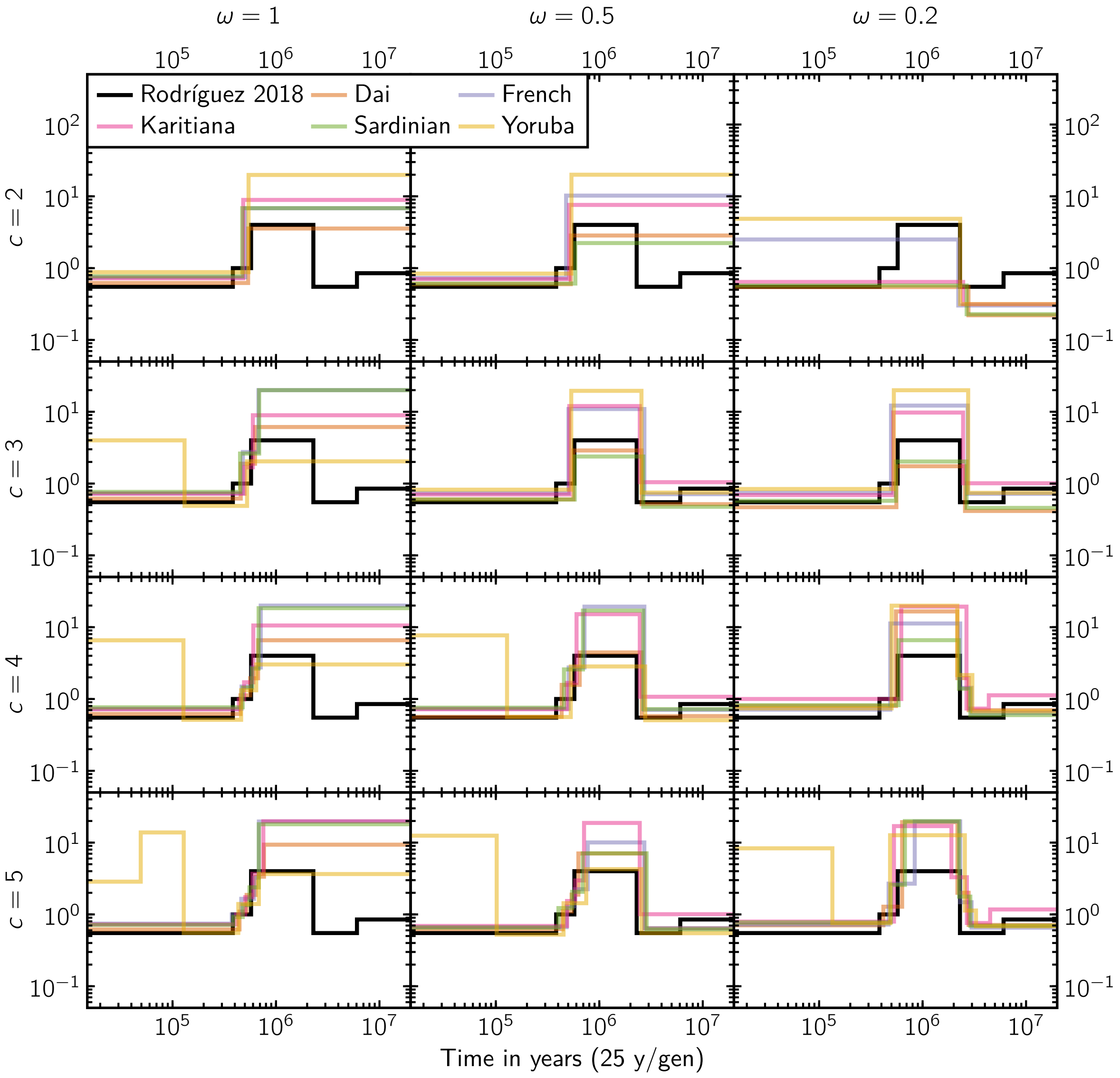

Figure S42: Connectivity graphs of the inferred human scenarios.

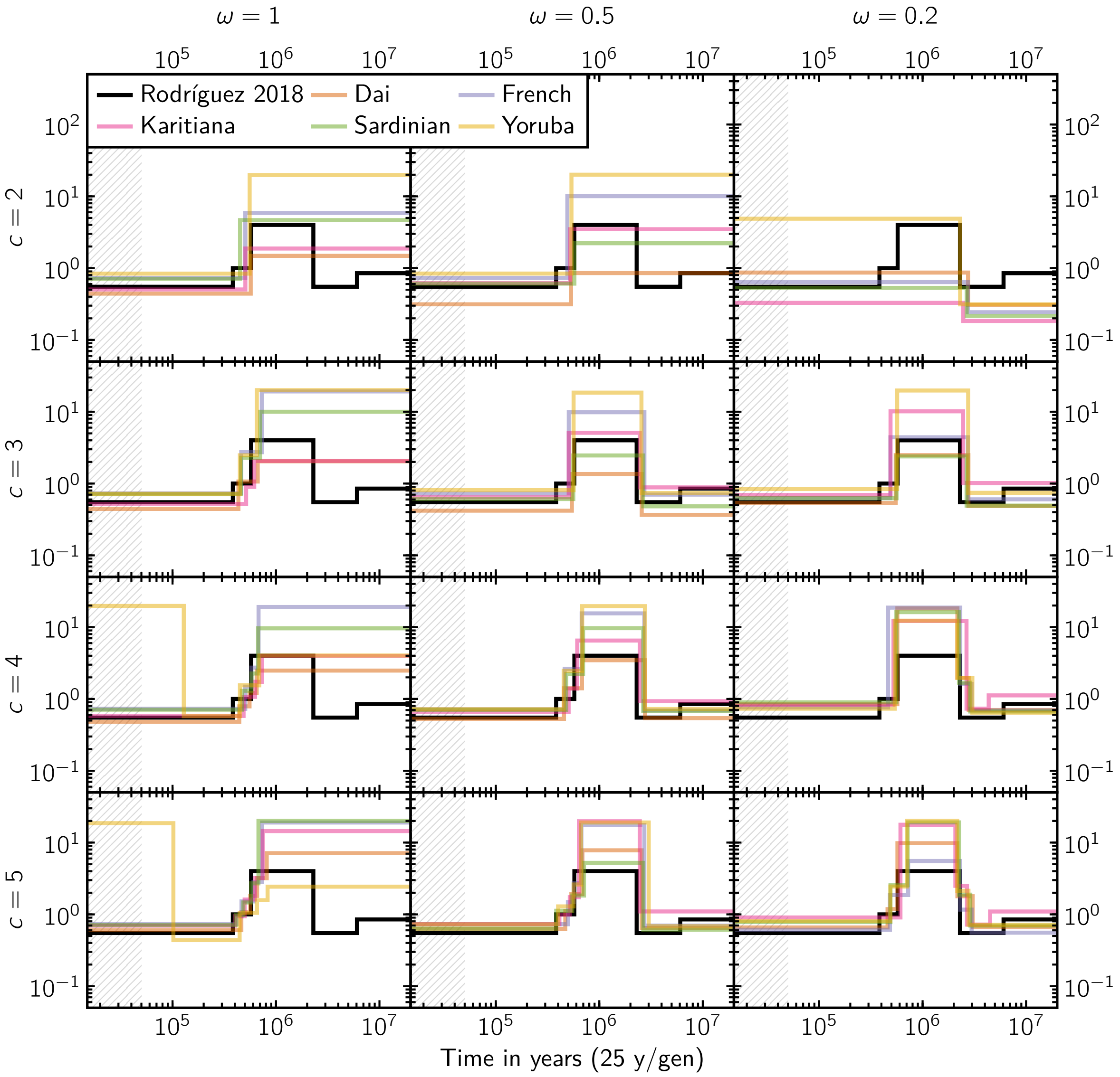

Figure S43: Connectivity graphs of the inferred human scenarios with restricted inference range.

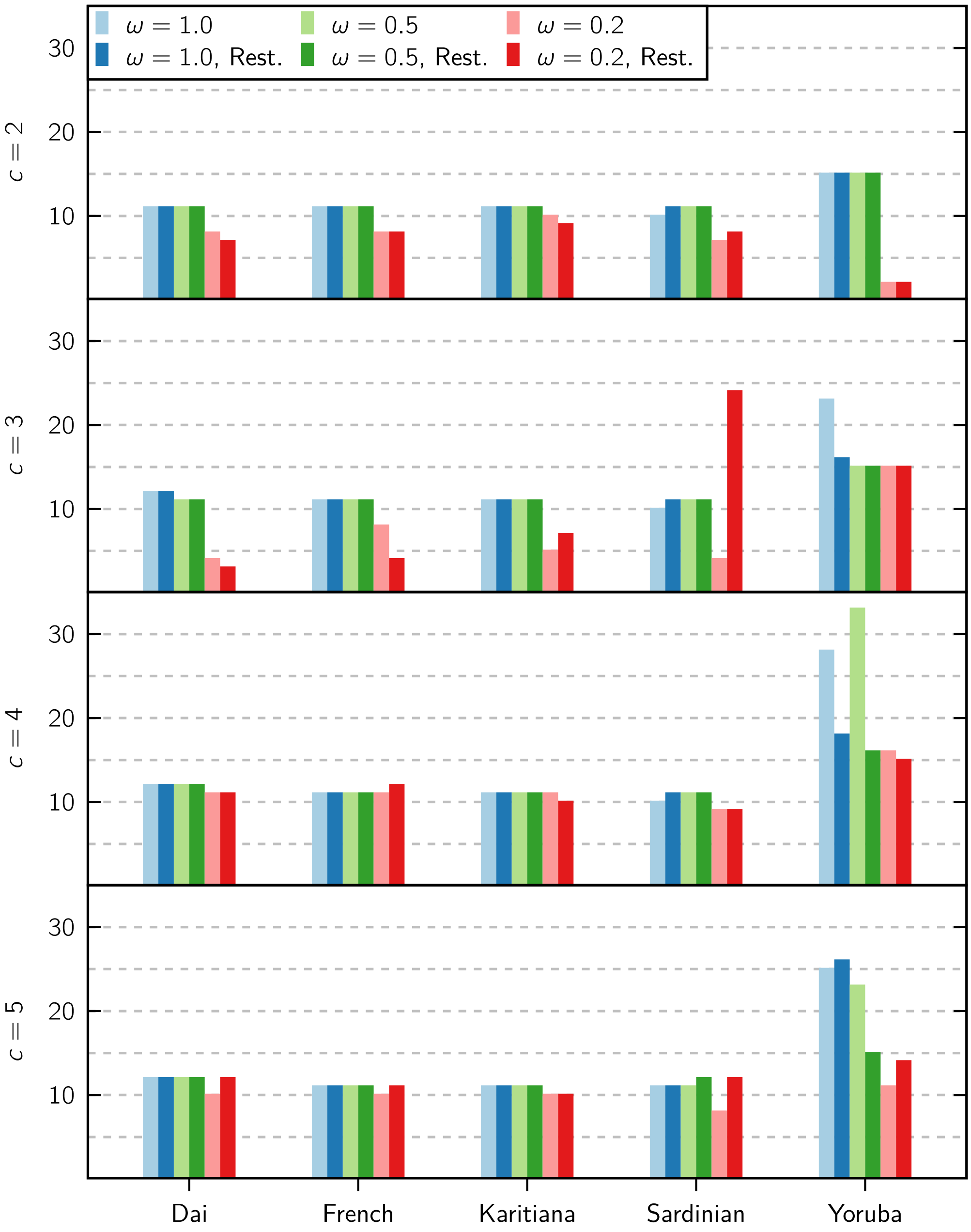

Figure S44: Inferred number of islands for the human populations.

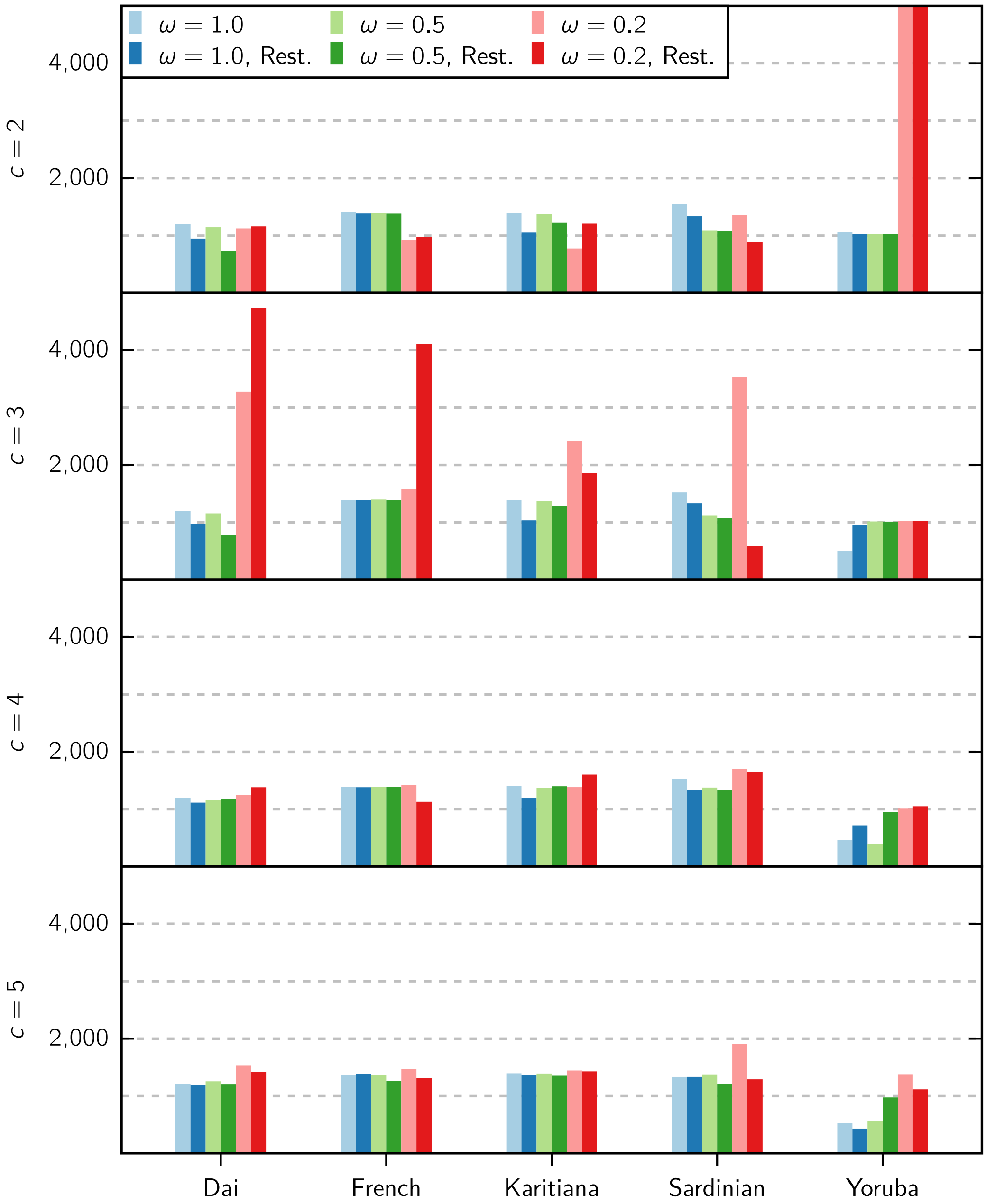

Figure S45: Inferred reference sizes for the human populations.

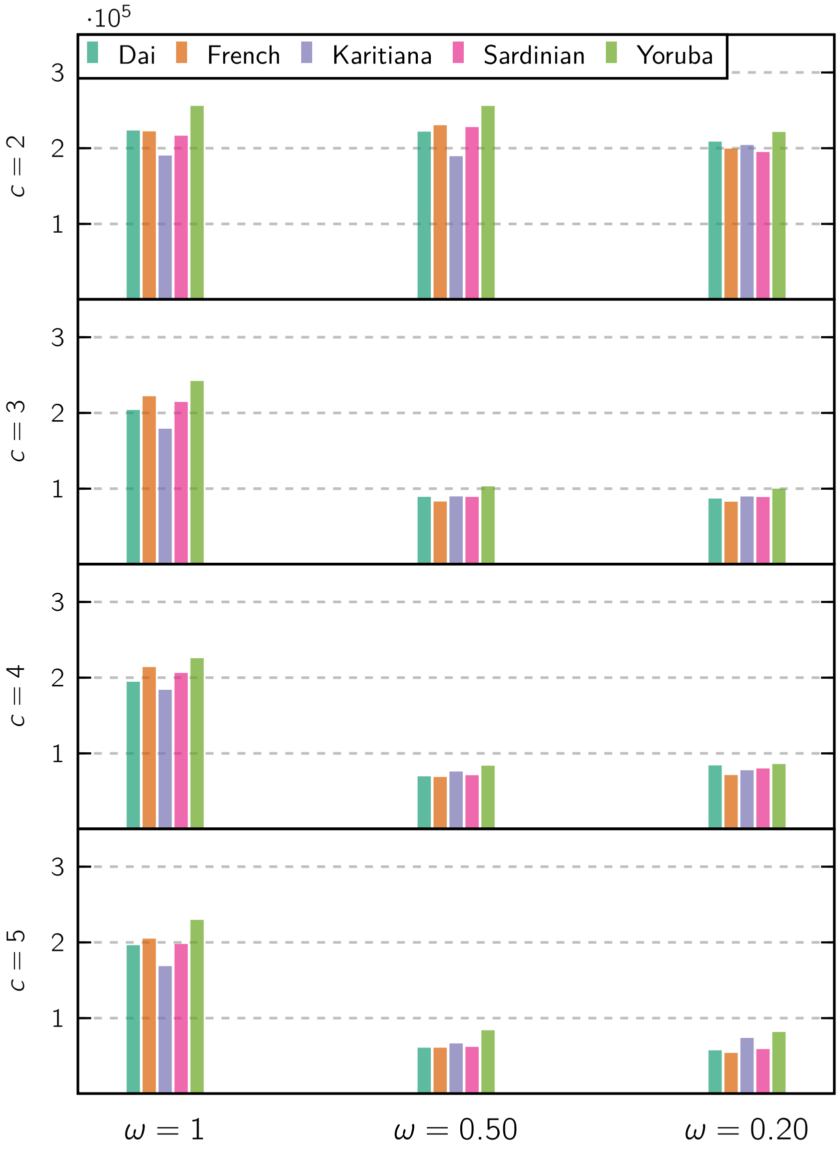

Figure S46: Visual distance (d_{visual}) of the best fitting scenario as a function of ω for various number of components.

S5.2. AFS comparisons

In this section we aim to make a basic comparison of the Allele Frequency Spectrum (AFS) corresponding to the demographic scenarios inferred by SNIF from human PSMC plots to the folded AFS of a sample of 108 humans from the Yoruba "population" (Lapierre et al. 2017), and to the AFS produced by the GADMA method (Noskova et al. 2019) for similar samples from the three populations of the C3PO model. This comparison is intentionally limited in scope because a full AFS study requires a separate study.

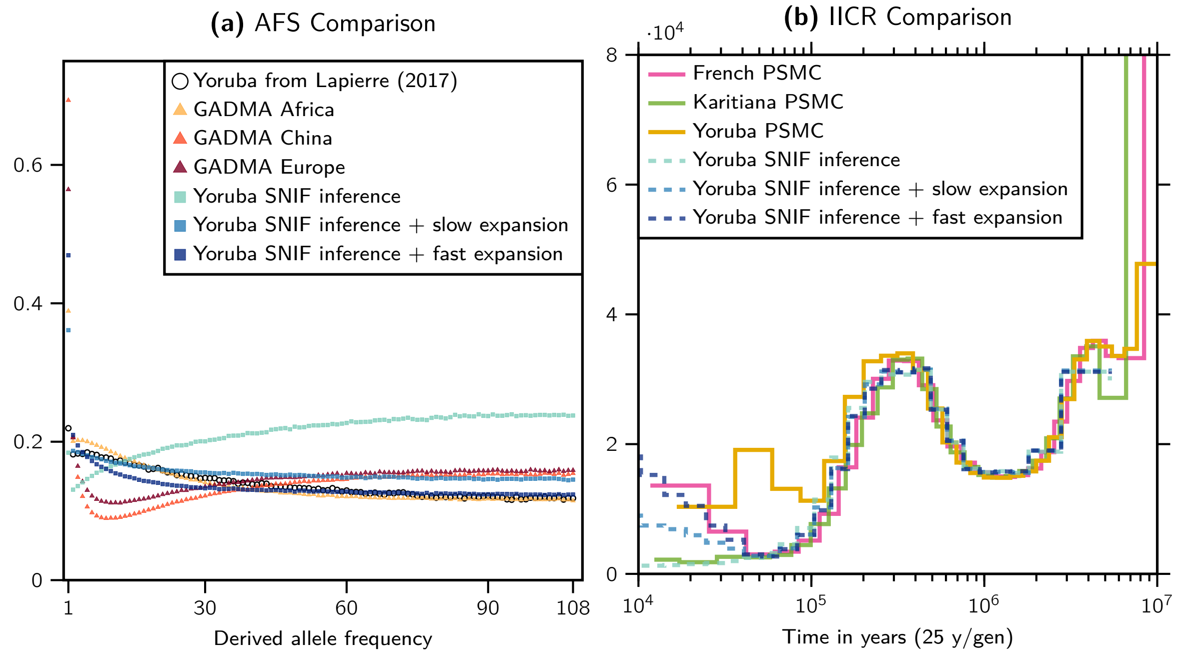

Figure S47: Comparison of **(a)** the folded AFSs and **(b)** the IICRs of various real, inferred and simulated human populations. The AFSs In panel **(a)** are transformed according to Lapierre et al. (2017) in order facilitate the visual comparison. The transformation consists in multiplying the ith frequency by i *before* folding (the details can be found in Lapierre et al. (2017) pages 440–441).

We simulated the AFS under two extreme models and an in-between one: one with constant size (i.e., the model inferred by SNIF from the Yoruba PSMC), another one with a recent exponential growth in the last 50,000 years (fast expansion), and another where the population growth was half as fast (slow expansion). The expansion parameters were chosen such that in the case of the fast expansion, the IICR matches in the recent past the human PSMCs that clearly exhibit a signal compatible with recent population expansion (French, for instance). The slow expansion is half as fast in the sense that in the present, the effective size is half as big. In Figure S47 panel (b) we plotted the corresponding three PSMCs to show how they look like and in panel (a) predicted the three corresponding AFSs which we compared with the AFSs from Lapierre et al. (2017) and Noskova et al. (2019). We find that this simple change in the recent history is enough to make a significant change of the predicted AFS. We do not try to fit any of the AFSs (Yoruba, European and Chinese). This is just a "proof of concept" simulation which suggests that existing AFSs could be easily fitted with a structured

model similar to those inferred by SNIF, but in which we would allow for a recent population size change which would incorporate geography.

S6. A note on implementation and use cases

Our method is implemented in a program named SNIF (Structured Non-stationary Inference Framework) which can be found at `github.com/arredondos/snif`. The method produces parameter estimates and connectivity graphs in order to determine whether there is consistency across individuals or species. We focused on models in which the population size was maintained constant. The method can in principle infer changes in population size but this should be part of an extension of the present work. At this stage we stress that more work is needed before changes in connectivity and population size can be estimated together with confidence.

The intended use case for our method consists of several inference cycles, each preceded by adjustments to the many available parameters which include: the bounds B for the estimated variables, the number of components c, the weight distance parameter ω, the time interval where the distance is to be computed, the number of inference rounds along with the tolerance ε, some parameters of the search algorithm, and other minor options. This cycle emerges naturally from the fact that the inference process itself runs fairly quickly (a few seconds per round), so it is feasible to prepare a script that generates inferences under several combinations of these parameters and later do a general assessment of what are the most plausible scenarios for the data depending on the visual fit, the consistency of the inferred demographic histories, the distribution of the distances, etc. The SNIF software already includes a number of auxiliary scripts that may be used in this later analysis stage, including for instance the automatic generation of figures similar to the sub-panels of figures 5 and 6 in the main text, where the target and inferred IICRs can be compared, and the nature of the best fitting scenarios can be understood using the connectivity graphs or the n and N plots.

As for the number of components c, specifying a higher value will generally (but not always) result in a better fit and a lower distance, however it also incurs in longer analysis times and diminishing returns on the new information present in the resulting inferred histories. These aspects can be balanced by increasing the value of c up to the point where the inferred demographic histories start converging (for instance, as more components are added, the connectivity graphs stop revealing new major events and any additional new degrees of freedom are only used to refine already existing details).

As part of the user inference cycles mentioned above, the bounds system can be configured to infer parameters for more strict or specialized symmetrical island models. For instance, it is possible to fix the number of inferred islands to be exactly 3 by setting $n_{\text{min}} = n_{\text{max}} = 3$ in the inference bounds B. Likewise, given an independent estimate of the reference size N for the data, this information may be used to process the corresponding scaled target IICR as an unscaled one using matching inference bounds for the parameter N.

An important point concerning scaling is that the supplied value of the mutation rate μ must be accurately specified when inferring demographic parameters from a PSMC curve.

This value is not used during the inference, but it is used in order to properly scale the PSMC curve and convert it into a target IICR, therefore it is important to provide the same value of μ that was used during the PSMC analysis. Otherwise, the only parameter that can be correctly inferred is n, since the rest of the parameters would only be accurate up to a scaling factor. This follows from a similar logic to that of Lemma 1.

We also suggest to use the hand-fitting Python script developed in Chikhi et al. (2018) as a complement to the automated inference process proposed in this work. You may want to compare the results obtained this way with the output of SNIF. Doing this will help make sense of the data, or help set the bounds for the many SNIF parameters.

References

Chikhi, L., Rodríguez, W., Grusea, S., Santos, P., Boitard, S., and Mazet, O. (2018). The IICR (inverse instantaneous coalescence rate) as a summary of genomic diversity: insights into demographic inference and model choice. *Heredity*, 120:13–24.

Herbots, H. M. J. D. (1994). *Stochastic models in population genetics: genealogy and genetic differentiation in structured populations*. PhD thesis.

Hobolth, A., Siri-Jegousse, A., and Bladt, M. (2019). Phase-type distributions in population genetics. *Theoretical Population Biology*, 127:16–32.

Lapierre, M., Lambert, A., and Achaz, G. (2017). Accuracy of demographic inferences from the site frequency spectrum: the case of the yoruba population. *Genetics*, 206(1):439–449.

Li, H. and Durbin, R. (2011). Inference of human population history from individual whole-genome sequences. *Nature*, 475(7357):493–496.

Mazet, O., Rodríguez, W., Grusea, S., Boitard, S., and Chikhi, L. (2016). On the importance of being structured: instantaneous coalescence rates and human evolution—lessons for ancestral population size inference? *Heredity*, 116(4):362.

Noskova, E., Ulyantsev, V., Koepfli, K.-P., O'Brien, S. J., and Dobrynin, P. (2019). GADMA: Genetic algorithm for inferring demographic history of multiple populations from allele frequency spectrum data. *bioRxiv*.

Prado-Martinez, J., Sudmant, P. H., Kidd, J. M., Li, H., Kelley, J. L., Lorente-Galdos, B., Veeramah, K. R., Woerner, A. E., O'Connor, T. D., Santpere, G., et al. (2013). Great ape genetic diversity and population history. *Nature*, 499(7459):471–475.

Rodríguez, W., Mazet, O., Grusea, S., Arredondo, A., Corujo, J. M., Boitard, S., and Chikhi, L. (2018). The IICR and the non-stationary structured coalescent: towards demographic inference with arbitrary changes in population structure. *Heredity*, 121(6):663–678.
